# An Acidic Tubulointerstitial Microenvironment Delays Cell‐Cycle Progression in Proximal Tubule Cells and Fibroblasts and Synergizes With Lactate to Drive Phenotypic Alterations

**DOI:** 10.1111/apha.70302

**Published:** 2026-09-07

**Authors:** Marie‐Christin Schulz, Virginie Dubourg, Lea Schaude, Natalie Schröder, Eva Janßen, Isabelle Görlich, Stefanie Ruhs, Michael Kopf, Michael Gekle

**Affiliations:** ^1^ Julius‐Bernstein Institute of Physiology, Martin Luther University Halle‐Wittenberg Halle Germany; ^2^ Department of Anesthesiology and Operative Intensive Care Medicine University Hospital Halle (Saale) Halle Germany

**Keywords:** cell cycle arrest, cell dedifferentiation, chronic kidney disease, fibrosis, inflammation, RNA sequencing, tubulo‐interstitial acidosis

## Abstract

**Background:**

Chronic kidney disease (CKD) is characterized by progressive tubulointerstitial inflammation and fibrosis, particularly affecting the proximal tubule. Both proximal tubular epithelial cells and interstitial fibroblasts contribute to CKD progression through cell‐cycle arrest and phenotypic transitions. The aim of the present study was to address whether lactic acidosis contributes to these processes.

**Methods:**

Human proximal tubular cells (HK‐2 cells) and fibroblasts (CCDSk cells) were exposed to lactic acid, hydrochloric acid, or sodium lactate. Transcriptomic changes were assessed by RNA sequencing followed by GO term enrichment as well as canonical pathway and upstream‐regulator analyses. Predicted effects on the cell cycle were validated experimentally using BrdU incorporation and digital microscopy‐based cell‐cycle analysis. Protein expression of IL‐6, c‐FOS, phospho‐JAK1 and JAK2, STAT3, SRF, and p38 was assessed by Western blot.

**Results:**

Transcriptomic analysis revealed synergistic effects of lactate and acidification on gene expression, which were associated with cell type‐specific phenotypic changes. Biological assays confirmed these predictions: HK‐2 cells underwent a sustained G1 arrest with reduced DNA synthesis, while CCDSk cells displayed only a transient arrest resolving after 48 h. Predictions further indicated that lactic acidosis amplifies these effects, driving fibrotic reprogramming in HK‐2 cells and promoting a phenotypic switch of CCDSk cells toward an inflammatory state.

**Conclusions:**

Our findings suggest that extracellular lactic acidosis primes tubular cells for fibrotic remodeling and CCDSk cells for inflammatory activation, and may therefore represent an initiating microenvironmental trigger for maladaptive changes in CKD progression.

## Practitioner Points

- Local tubulointerstitial acidosis slows cell‐cycle progression in proximal tubular cells persistently and fibroblasts transiently, thereby increasing the vulnerability of both cell types to further pathophysiological alterations.
- Extracellular acidosis and simultaneously elevated extracellular lactate concentrations in the interstitial space are characteristic of lactic acidosis and have synergistic effects on both proximal tubule cells and fibroblasts. Lactic acidosis promotes a fibrotic response in proximal tubular cells and an inflammatory response in fibroblasts.

**Abbreviations**
AFC: 7‐amino‐4‐trifluoromethylcoumarin
AKI: acute kidney injury
AP‐1: activator protein‐1
BCA: bicinchoninic acid
BrdU: Bromodeoxyuridine
BSA: bovine serum albumin
CCD‐1092Sk: human dermal fibroblast cell line
CDK: cyclin dependent kinase
CDKN2A: cyclin dependent kinase inhibbitor 2A
CKD: chronic kidney disease
CO_2_
: carbon dioxide
CP: canonical pathway
DAPI: 4′,6‐diamidino‐2‐phenylindole
DEG: differentially expressed gene
DMEM: Dulbecco's Modified Eagle Medium
DNA: deoxyribonucleic acid
EGFR: epidermal growth factor receptor
EIF2AK4: Eukaryotic Translation Initiation Factor 2 Alpha Kinase 4
ELISA: enzyme‐linked immunosorbent assay
ERK: extracellular signal‐regulated kinase
FCS: fetal calf serum
FDR: false discovery rate
FGF: fibroblast growth factor
FPM: fragments per million
GO: gene ontology
HCl: hydrochloric acid
HK‐2: human proximal tubular epithelial cell line
IL: interleukin
IPA: ingenuity pathway analysis
JAK: janus kinase
MAPK: mitogen‐activated protein kinase
NRF2: nuclear factor erythroid 2‐related factor 2
PBS: phosphate buffered saline
PT: proximal tubule cells
RB: retinoblastoma protein
RIN: RNA integrity number
RIPA: radioimmunoprecipitation assay buffer
RNA: ribonucleic acid
RRAS: ras‐related protein
RXRA: retinoid X receptor alpha
SCAP: SREBP cleavage activating protein
SDS‐PAGE: sodium dodecyl sulfate‐polyacrylamide gel electrophoresis
SREBF1: sterol regulatory element‐binding transcription factors
SRF: serum response factor
STAT: signal transducers and activators of transcription
STRING: search tool for the retrieval of interacting genes/proteins
TGF‐ß: transforming growth factor ß
TMM: trimmed mean of M values
TRIS: Tris(hydroxymethyl)aminomethan
URA: upstream regulator analysis

## 1 Introduction

Chronic kidney disease (CKD) affects approximately one in 10 individuals worldwide and ranks among the top 10 causes of global mortality [1]. Despite its clinical impact, causal therapies remain unavailable, partly because the mechanisms driving CKD progression are not yet fully understood. A central feature of disease progression is remodeling of the tubulointerstitial compartment, involving proximal tubular epithelial cells and fibroblasts [2]. Tubulointerstitial remodeling is accompanied by disturbed epithelial–fibroblast communication and the development of a local extracellular lactic acidosis [3].

Sources of local lactic acidosis are injured proximal tubules that undergo a metabolic shift from fatty acid oxidation to anaerobic glycolysis, increased lactate dehydrogenase activity, mitochondrial dysfunction, and impaired bicarbonate handling [4, 5, 6]. Moreover, resident fibroblasts and infiltrating immune cells may also contribute to local interstitial acidification [3]. Progression of CKD is closely linked to phenotypic changes in resident cells. In proximal tubular cells, acute kidney injury (AKI) often leads to cell loss, with repair depending on the proliferative capacity of surviving cells [7]. Cell cycle arrest in this context has been identified as a maladaptive mechanism that promotes fibrotic transformation and represents a key risk factor for AKI‐to‐CKD transition [8, 9]. Fibroblasts, by contrast, display remarkable plasticity [10]. In early CKD stages, fibroblasts adopt an inflammatory phenotype characterized by enhanced cytokine secretion and reduced matrix synthesis, whereas in later stages they shift toward a profibrotic state that drives interstitial fibrosis [11].

Although acidosis is a common feature of CKD and is known to induce profound pathophysiological changes in multiple cell types, the impact of lactic acidosis on the CKD‐associated phenotypic responses of human proximal tubular cells and fibroblasts has not yet been systematically investigated.

This study therefore aimed to explore the role of lactic acidosis as a microenvironmental driver of tubulointerstitial remodeling. Using RNA sequencing of human proximal tubular cells (HK‐2) and human fibroblasts (CCDSk cells), we examined whether the observed effects were attributable to reduced extracellular pH, elevated extracellular lactate concentration, or the combination of both. Our findings indicate that reduced extracellular pH delays the cell‐cycle progression in both cell types, thereby increasing their susceptibility to subsequent microenvironmental cues. When combined with elevated extracellular lactate, acidosis is predicted to promote fibrotic responses in HK‐2 cells and an inflammatory phenotype in CCDSk cells—alterations characteristic of CKD progression.

## 2 Results

### 2.1 Hydrochloric Acid‐ and Lactic Acid‐Induced Extracellular Acidosis Triggers Gene Expression Changes Associated With Cell Cycle Arrest in Proximal Tubule Cells

**FIGURE 1:**
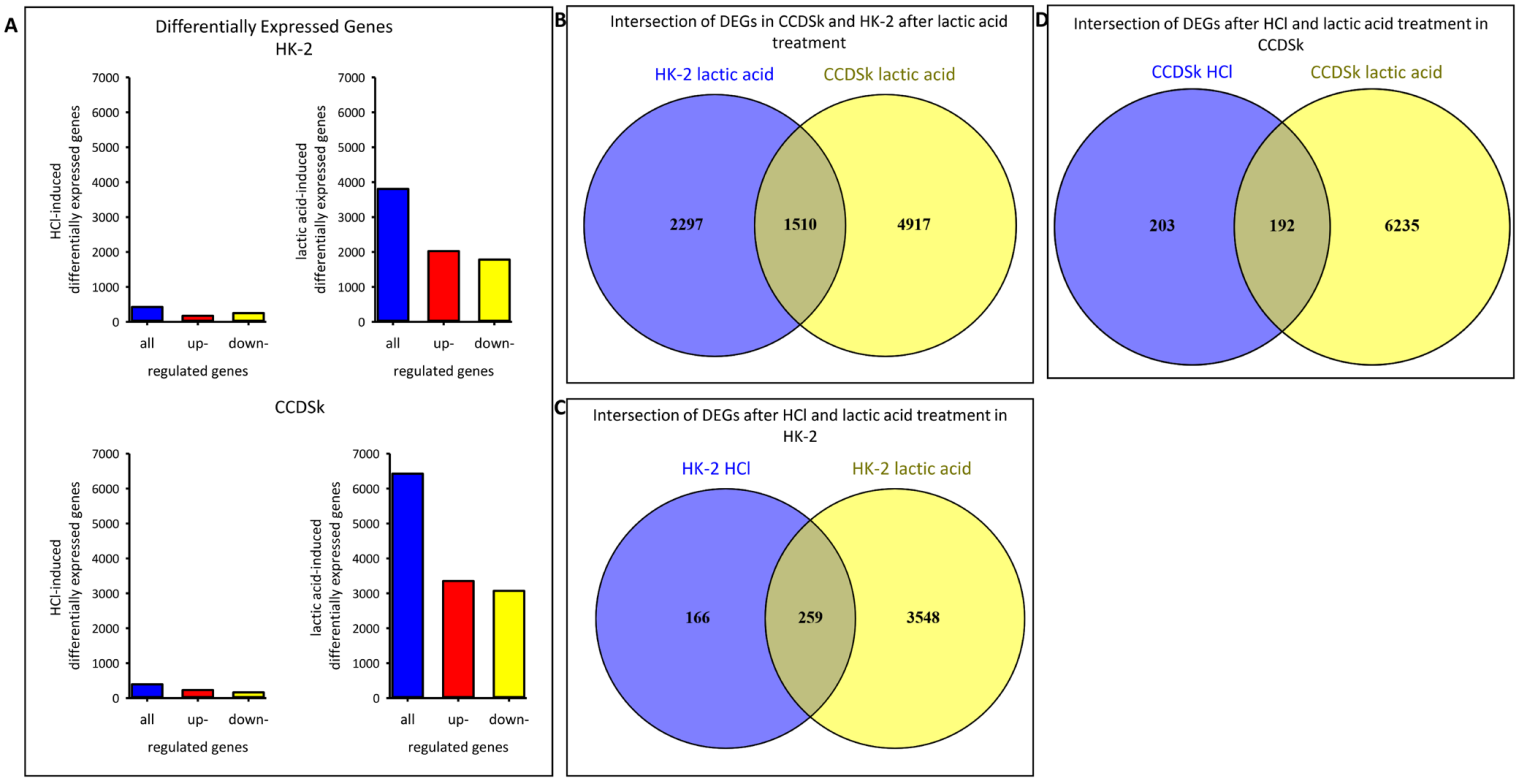
Lactic acidosis‐induced gene expression. (A) Number of differentially expressed genes (DEGs) after incubation with hydrochloric acid and lactic acid period (B) Intersection of HK‐2 cells and CCDSk cells after exposure to lactic acid medium and (C) intersection of DEGs after incubation with hydrochloric acid and lactic acid in HK‐2 cells. (D) Intersection of DEGs after incubation with hydrochloric acid and lactic acid in CCDSk cells. Number of independent replicates = 5–6.

As shown in Table 1, the extracellular pH remained stable over the 48‐h incubation period under control conditions in both cell types and showed only minor changes under acidic conditions, confirming the stability of the experimental conditions. Lactic acidosis combines a reduced extracellular pH, an increased lactate concentration, as well as an increased osmotic pressure. Therefore, cells were incubated for 48 h in medium containing lactic acid, hydrochloric acid (to investigate the impact of acidosis), sodium lactate, or mannitol (to investigate the impact of increased osmolality). RNA sequencing followed by differential expression analysis showed that, after incubation with lactic acid, 3807 genes were regulated in HK‐2 cells (2025 up, 1782 down) and 6427 in CCDSk cells (3355 up, 3072 down) (Figure 1A), of which 1510 overlapped (Figure 1B). Hydrochloric acidosis altered the expression of 425 genes (173 upregulated and 252 downregulated) in HK‐2 cells, of which 259 overlapped with those altered by lactic acidosis (Figure 1C). In CCDSk cells, hydrochloric acidosis altered the expression of 395 genes (231 upregulated and 164 downregulated), of which 192 overlapped with those altered by lactic acidosis (Figure 1D). An overlay histogram illustrating the distribution of DEGs in both treatment groups is provided in Figure S8 and the complete list of differentially expressed protein‐coding genes is available in Table S4. Neither sodium lactate nor mannitol affected the number of DEGs, suggesting that extracellular acidification is the primary driver of the observed transcriptional changes.

**TABLE 1:** Alterations of extracellular pH values after 48 h.

Mean ± SD			
Group	Control media (without cells)	HK‐2	CCDSk
Control	7.45 ± 0.01	7.45 ± 0.02	7.44 ± 0.04
L‐Lactic acid	6.40 ± 0.05	6.35 ± 0.04	6.38 ± 0.08

*Note:* The impact of extracellular lactic acidosis on HK‐2 cells and CCDSk cells was investigated.

**FIGURE 2:**
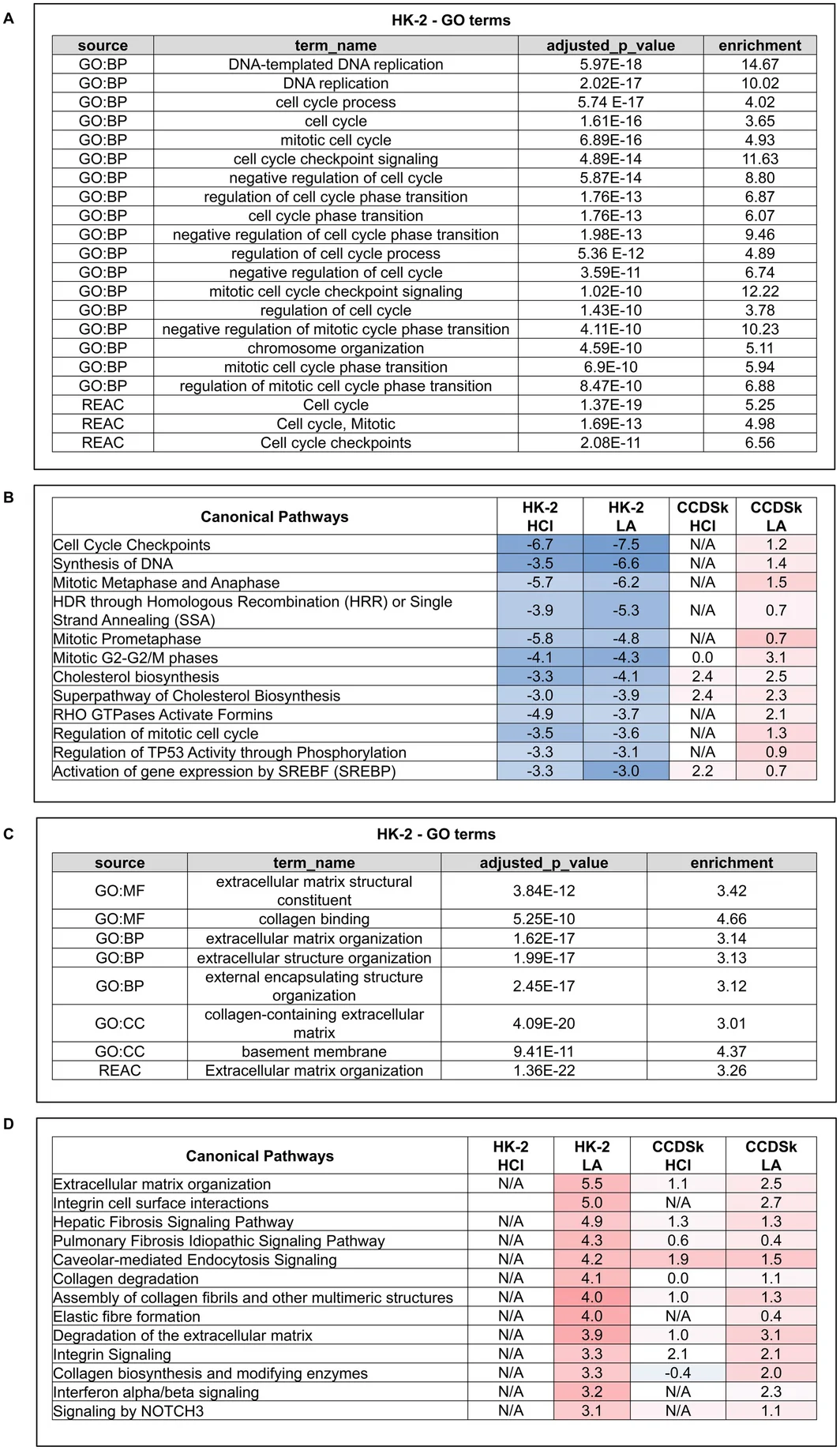
IPA or g:Profiler analysis of acidosis‐induced gene expression changes (DEGs) predicts cell‐cycle arrest and fibrosis in HK‐2 cells. (A) GO terms that were significantly enriched in downregulated DEGs in HK‐2 cells after incubation with lactic acid. (B) Comparative analysis of canonical pathway analysis by IPA with a focus on cell‐cycle arrest. (C) GO terms that were significantly enriched in upregulated DEGs in HK‐2 cells after incubation with lactic acid. (D) Comparative analysis of canonical pathway analysis by IPA with a focus on fibrotic response.

To assess the functional relevance of these acidosis‐induced genes in HK‐2 cells and CCDSK cells, we performed Gene Ontology (GO) term enrichment analysis. Among the downregulated genes in HK‐2 and after filtering as described in section 4.6, 19 GO terms were enriched and associated with cell cycle or its regulation (Figure 2A). In contrast, Gene Ontology (GO) enrichment analysis of CCDSk cells identified only a single significantly enriched term in the hydrochloric acid group (“muscle structure development”; adjusted *p* = 2.8 × 10^−9^, enrichment score = 3.7). Moreover, for the intersecting DEGs, no enriched GO terms passed the significance threshold. The complete list of enriched terms is provided in Table S5. To predict the direction of acidosis‐induced cell cycle regulation in HK‐2 cells, we performed IPA Canonical Pathway Analysis. Acidosis‐induced DEGs were enriched in canonical pathways mostly linked to cell cycle control, including “Cell Cycle Checkpoints,” “DNA Synthesis,” and “DNA Replication Pre‐Initiation” (Figure 2B). Inhibition of these pathways was predicted. These findings suggested that extracellular acidosis delays cell cycle progression in HK‐2 cells, a hypothesis tested in subsequent experiments. In contrast, the same cell cycle pathways were predicted to be activated (e.g., “DNA Replication Pre‐Initiation”) or remained unchanged in CCDSk cells exposed for 48 h to lactic acid (Figure 2B). Notably, no canonical pathways remained in the CCDSk cells hydrochloric acid group after applying the filtering criteria. Overall, these predictions indicate that the acidosis‐induced cell cycle arrest may be cell type specific.

### 2.2 Hydrochloric‐ and Lactic Acidosis Induce a Long‐Lasting Cell‐Cycle Arrest in HK‐2 Cells

**FIGURE 3:**
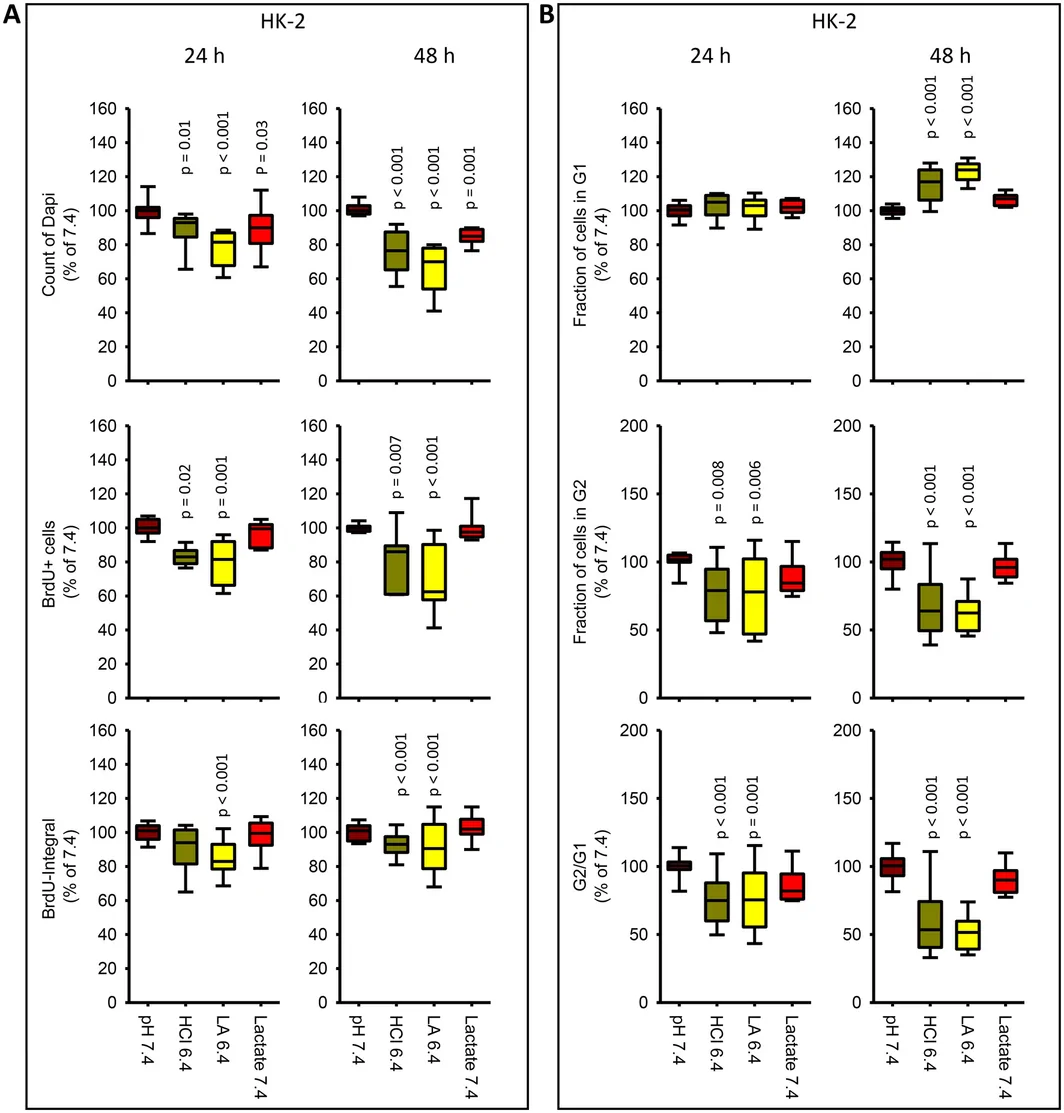
(A) Number of nuclei, BrdU+ cells, and BrdU incorporation (BrdU‐Integral) in HK‐2 cells after 24 and 48 h. (B) Fractions of HK‐2 cells in the G1 and G2 cell‐cycle phases, and the corresponding ratios. Number of independent biological replicates *n* = 12–24. Differences are statistically significant when *p* < 0.05.

To validate the predicted acidosis‐induced cell‐cycle arrest, we measured DNA synthesis (BrdU incorporation) and analyzed cell cycle distribution for HK‐2 cells. After 24 h and 48 h in acidic medium, BrdU incorporation was significantly decreased, both in total signal intensity and in the fraction of BrdU‐positive (BrdU^+^) cells. This was accompanied by a reduction in cell number (Figure 3A). This decrease was also reflected in the total cellular protein content. In contrast, neither caspase‐3 activity, a marker of apoptosis, nor caspase‐3 release, a marker of necrosis, was altered in HK‐2 cells after 48 h of incubation with lactic acid (Figure S2A). These findings indicate that apoptosis or necrosis is unlikely to account for the acidosis‐induced reduction in cell number. Cell cycle analysis revealed fewer cells in G2 after 24 h and an additional accumulation in G1 after 48 h under acidic conditions (Figure 3B). In contrast, sodium lactate exposure did not alter BrdU incorporation nor cell cycle distribution (Figure 3A,B). Notably, sodium lactate also induced a significant reduction in the number of nuclei, although this effect was less pronounced than that induced by extracellular acidosis (Figure 3A).

### 2.3 Hydrochloric‐ and Lactic Acidosis Induce a Transient Cell‐Cycle Arrest in CCDSk Cells

**FIGURE 4:**
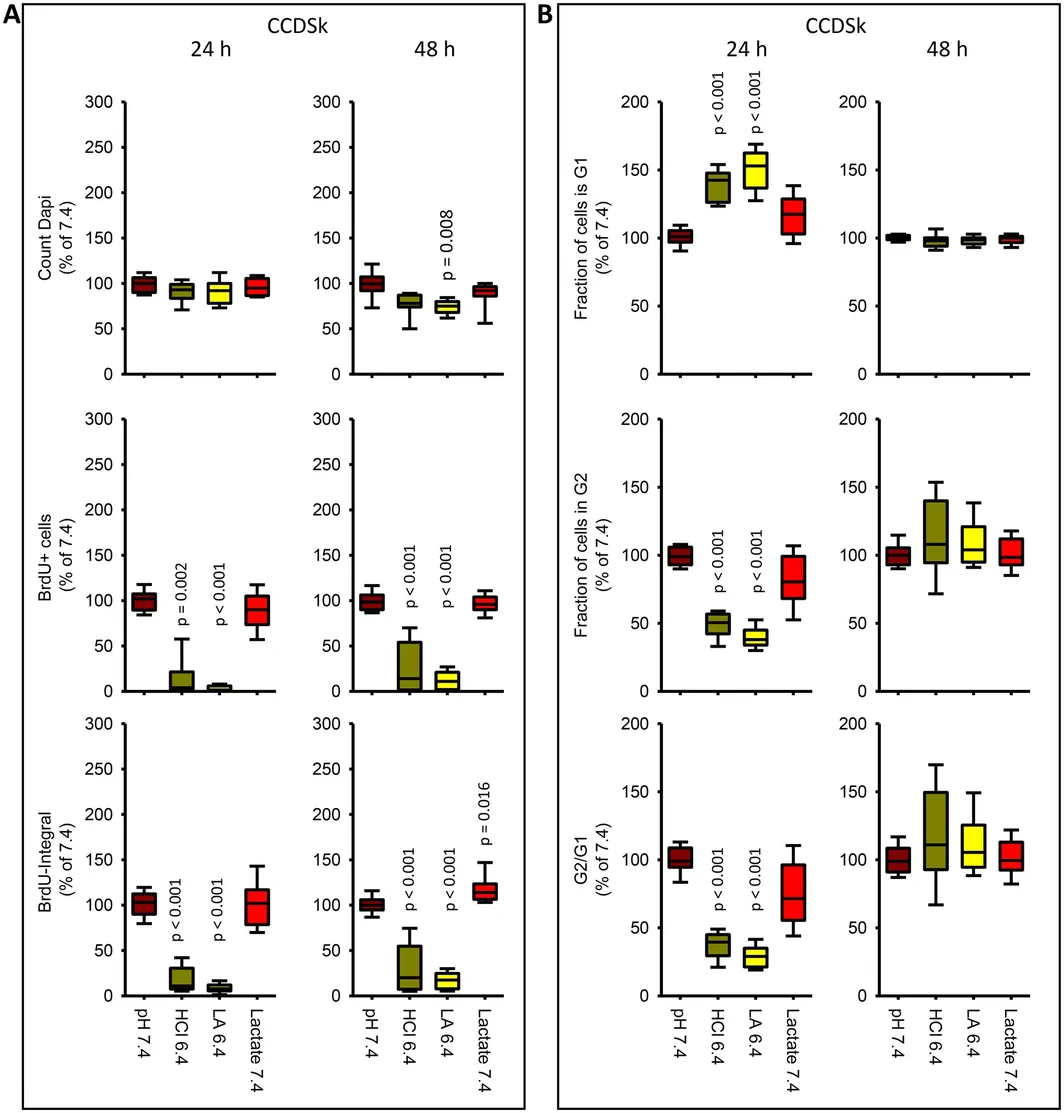
(A) Number of nuclei, BrdU^+^ cells, and BrdU incorporation (BrdU‐Integral) in CCDSk cells after 24 and 48 h. (B) Fractions of CCDSk cells in the G1 and G2 cell‐cycle phases and the corresponding ratios. Number of independent biological replicates, *n* = 12–24. Differences are statistically significant when *p* < 0.05.

To test whether acidosis‐induced cell‐cycle arrest is cell type–specific, as predicted by RNA sequencing data analysis, the cell cycle analysis was also performed in CCDSk cells. Incubation with acidic medium reduced DNA synthesis after 24 h, reflected by lower BrdU incorporation and fewer BrdU+ cells, and with a weaker effect after 48 h (Figure 4A). Lactic acidosis reduced BrdU incorporation to 10% at 24 h and 25% at 48 h, with fractions of BrdU+‐positive cells of 3% and 14%, respectively (Figure 4B). Cell cycle analysis showed increased G1 and decreased G2 fractions after 24 h in acidic medium. These effects were no longer detectable at 48 h (Figure 4B). In CCDSk cells, sodium lactate neither impaired DNA synthesis nor altered cell cycle distribution (Figure 4A,B).

Additionally, none of the treatments affected total protein amount or caspase‐3 activity, and the caspase‐3 release was below the detection limit (Figure S2).

### 2.4 Predicted Involvement of the CDKN–RB–E2F Axis in Hydrochloric Acid‐ and Lactic Acid‐Induced Cell‐Cycle Arrest in HK‐2 Cells

**FIGURE 5:**
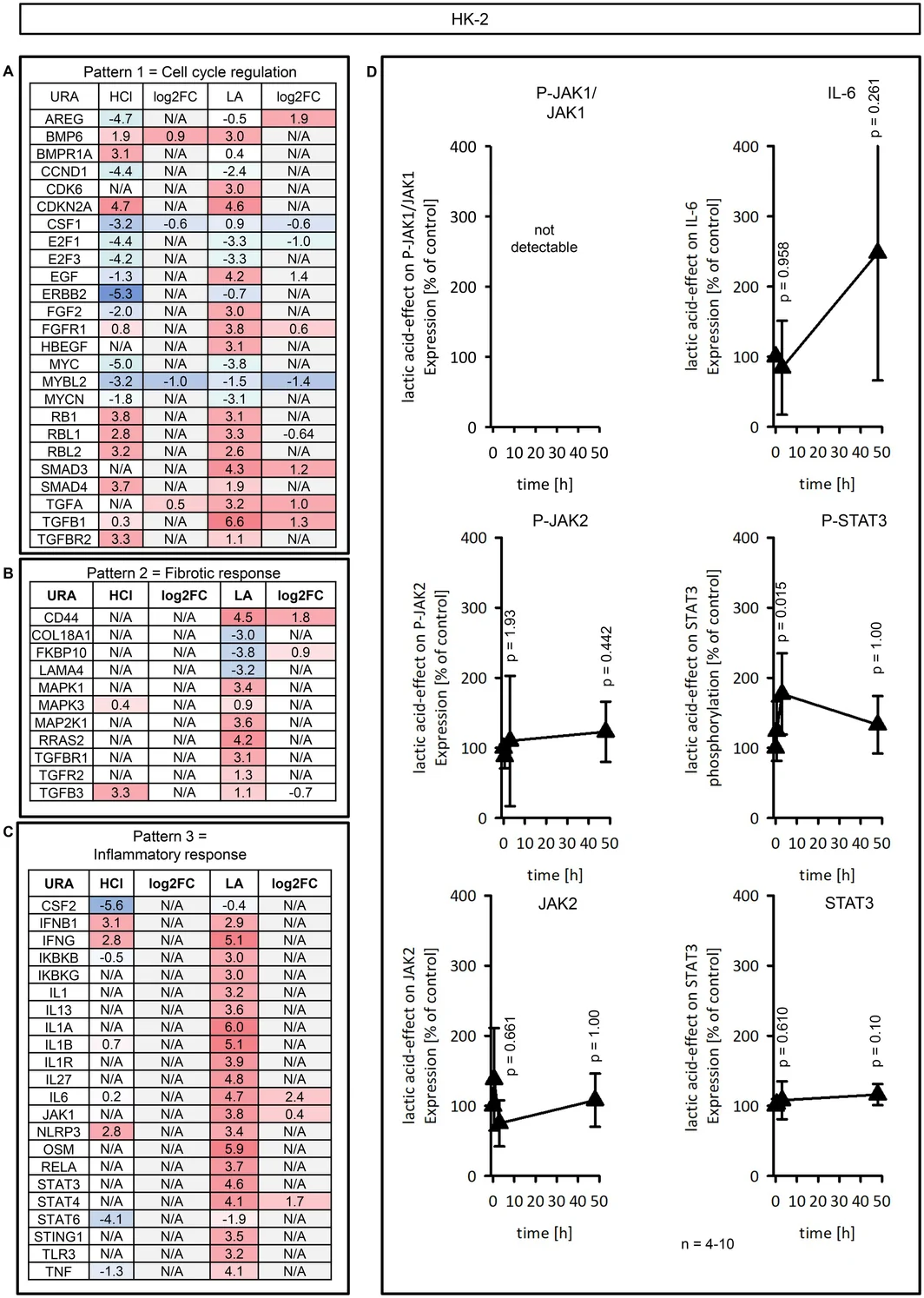
Predicted upstream regulators in HK‐2 cells for (A) cell cycle arrest, (B) fibrotic response, (C) inflammatory response, and (D) Western blot results for IL‐6, JAK1, JAK2, and STAT3 as well as P‐JAK1, P‐JAK2, and P‐STAT3 after 30 min, 3 h, and 48 h. Number of independent biological replicates, *n* = 4–10. Data are shown as mean ±95% CI. Differences are statistically significant when *p* < 0.05. All Western blot data are given as a percentage of control (pH 7.4).

To identify possible mediators of acidosis‐induced cell‐cycle arrest in HK‐2 cells, we used the “Upstream Regulator Analysis” tool of IPA (See Table S11). Due to the high number of upstream regulators, an extended analysis pipeline was applied as described in the Methods section 4.7.2. Using this approach, 25 upstream regulators associated with cell cycle regulation were identified (Figure 5A). Among these, regulators predicted to be altered in the same direction following both hydrochloric acid‐ and lactic acid‐induced acidification were selected for further analysis, as cell‐cycle arrest was observed under both conditions. This filtering resulted in the identification of CDKN2A (activated), E2F1 (inhibited), E2F3 (inhibited), MYC (inhibited), and RB (activated) as concordantly predicted upstream regulators (Figure 5A). Within the classical biological hierarchy, CDKN2A acts at the top of the causal cascade. Increased CDKN2A activity inhibits CDK4/6, activating RB family proteins that repress E2F1 and E2F3, resulting in cell‐cycle arrest. This effect is further reinforced by concomitant MYC inhibition. Accordingly, we hypothesize that acidification induces a CDKN2A‐mediated CDK4/6 brake, activating RB1/RBL1/RBL2 and suppressing E2F1/E2F3‐dependent S‐phase gene expression, with MYC inhibition further strengthening this antiproliferative state.

### 2.5 Lactic Acidosis Potentially Induces a Fibrotic Response in HK‐2 Cells

The analysis of canonical signaling pathways by IPA predicted a relevant impact only for lactic acidosis. Taken together, predicted pathways induced by lactic acidosis, including “Assembly of collagen fibrils and other multimeric structures”, “Collagen degradation” or “Hepatic fibrosis signaling pathway” suggest that lactic acidosis induces a fibrotic response in HK‐2 cells (Figure 2D). This finding is further supported by the enrichment analysis of g:Profiler, since the corresponding GO terms (in total, 6) contain “collagen binding” or “extracellular matrix organization” (Figure 2C).

### 2.6 The Predicted Lactic Acid‐Induced Fibrotic Response Is Possibly Mediated by CD44 Induced MAPK Signaling

To identify mediators of the predicted lactic acidosis‐induced fibrotic response, we focused on upstream regulators predicted predominantly in the lactic acid group. This analysis revealed SMAD3 and TGFB1, classified within pattern 1 (“cell‐cycle analysis”) CD44, MAPK1 (ERK2), MAP2K1, RRAS2, and TGFBR1, from pattern 2 (“fibrotic response”) and several cytokines classified within pattern 3 (“inflammatory response”) (Figure 5A–C). Both CD44 and TGFBR1 can be activated in response to extracellular stimuli and initiate intracellular signaling. CD44 is classically linked to activation of RAS‐family signaling (e.g., RRAS) and thereby the MEK/ERK pathway, and it can interact with TGFBR1, thereby promoting SMAD3/4‐dependent transcription. Activation of this signaling cascade is commonly associated with a profibrotic response. Accordingly, we propose as a working hypothesis that extracellular lactic acidosis initiates a fibrotic program in HK‐2 cells via activation of a CD44/TGFBR1 axis. Given that fibrosis often develops in the context of chronic inflammation, pattern 3 (“inflammatory response”) was subsequently examined as a potential source of mediators contributing to the fibrotic response. This pattern predicted multiple cytokines as upstream regulators; because cytokines are typically regulated at the transcriptional level, evidence level III (concordant directions of log_2_FC and *Z*‐score) was applied for these cytokines. Thus, IL‐6 emerged as the only potential mediator (*Z*‐score = 4.7, log_2_FC = 2.4). To test whether IL‐6 signaling contributes to lactic acidosis‐induced fibrosis, IL‐6 and members of its canonical pathway (JAK/STAT3) were analyzed by Western blot. Lactic acid treatment in HK‐2 cells did not increase IL‐6 and JAK2 protein levels, nor JAK2 phosphorylation, and JAK1 was not detectable (Figure 5D). In contrast, phosphorylated STAT3 was elevated transiently after 3 h. These results reveal an IL‐6‐independent STAT3 phosphorylation as a potential mediator of the lactic acidosis‐induced fibrotic response in HK‐2 cells.

### 2.7 Lactic Acidosis‐Induced Gene Expression Changes Indicate a Phenotypic Switch in Fibroblasts

GO term enrichment analysis of upregulated genes in CCDSk cells after incubation with lactic acid revealed a strong enrichment of terms related to translation (in total, 8 terms), such as “translation factor activity”, “cytoplasmic translation”, and “cytosolic ribosome” (Table S9). In contrast, no enriched terms remained for the downregulated genes after filtering. This pattern was also reflected in the canonical pathway analysis by IPA, which predicted the activation of pathways related to translation.

**FIGURE 6:**
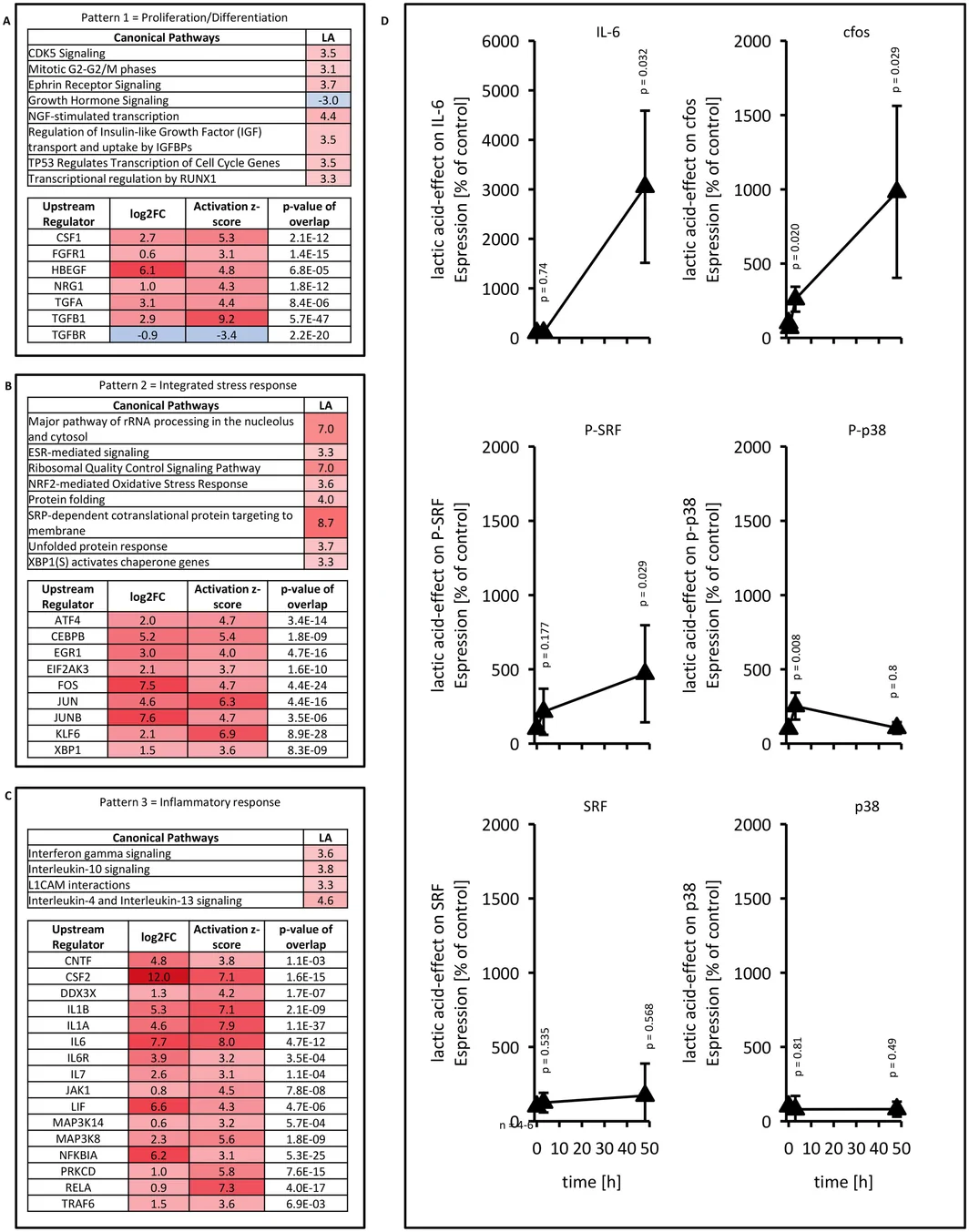
Analysis of canonical pathways in CCDSk cells after incubation with lactic acid. Red fields mark predicted activation (*Z*‐score > 3) and blue fieldS mark predicted inhibition (*Z*‐score < −3). Predicted canonical pathways and upstream regulators for (A) inflammatory response, (B) integrated stress response, and (C) proliferation/differentiation in CCDSk cells. (D) Western blot results for IL‐6, cFOS, SRF, and p38 as well as P‐SRF, and P‐p38, after 3 h and 48 h. Number of independent biological replicates, *n* = 4–6. Data are shown as mean ±95% CI_,_ Differences are statistically significant when *p* < 0.05. All Western blot data are given as a percentage of control (pH 7.4).

Overall, the results predict the activation of pathways associated with differentiation/proliferation, including “CDK5”, “Growth factor signaling”, “TP53”, and “RUNX1” (Figure 6A).

Figure 6B shows a list of pathways associated with the integrated stress response which were predicted to be activated, including “NRF2‐mediated stress response”, and “SRP‐dependent cotranslational protein targeting to membrane”. Furthermore, several predictions indicated activation of cytokine signaling, consistent with an inflammatory response (Figure 6C). In summary, the activation of these canonical pathways suggests lactic acidosis‐induced activation of CCDSk cells toward an inflammatory phenotype in response to stress.

### 2.8 EGFR‐Signaling as a Candidate Pathway Mediating Lactic Acidosis‐Induced Differentiation of Fibroblasts

**FIGURE 7:**
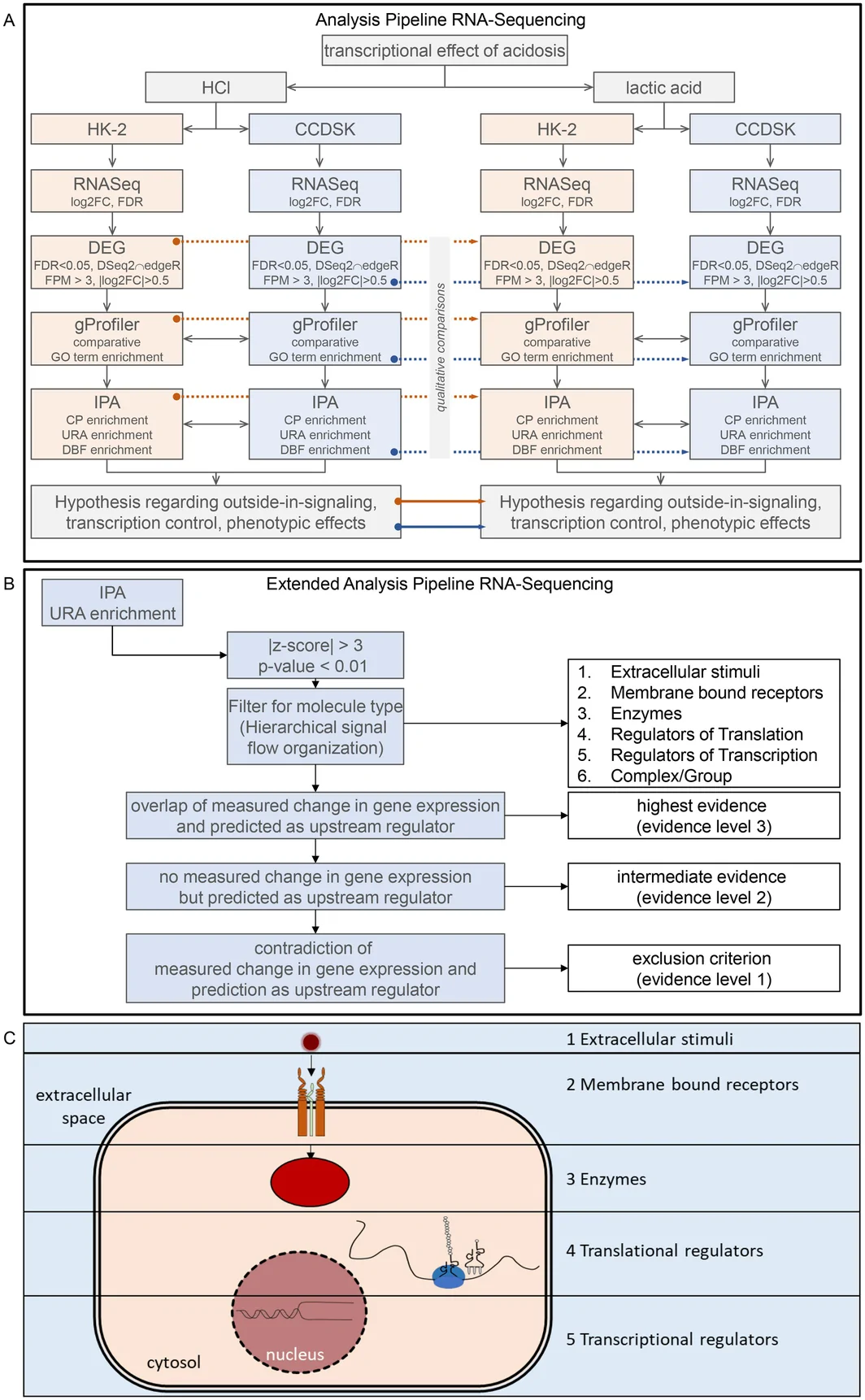
(A) Work flow for RNA‐sequencing data analysis. (B) Extended analysis pipeline for the categorization of potential upstream regulators (i.e., cellular elements putatively involved in the changes in gene expression measured by RNA sequencing) predicted by IPA (Upstream regulator analysis). (C) Scheme of classical cellular signal flow from extracellular signaling to cellular response.

Even after application of the stringent analysis scheme shown in Figure 7B, a large number of upstream regulators (*n* = 305) were predicted for CCDSk cells following lactic acid incubation (Table S12).

To identify the most promising candidates, only upstream regulators assigned to evidence level III (concordant directions of log_2_FC and *Z*‐score) were considered for further analysis. Thus, the number was reduced to 116. Using subsequent STRING analysis, three functional patterns were identified (See Tables S13 and S14). Pattern 1 was defined as “Proliferation/Differentiation” (Figure 6A), pattern 2 as “Integrated stress response” (Figure 6B), and pattern 3 as “Inflammatory response” (Figure 6C).

The prediction of canonical pathways, together with the experimentally observed transient cell cycle arrest, suggests that lactic acidosis exposure may induce a differentiation process in CCDSk cells. In support of this notion, the upstream regulator analysis of pattern 1 indicated that lactic acidosis may mediate this differentiation via activation of EGFR signaling. This interpretation is based on the prediction of FGF, HBEGF, TGFA, and TGFB1 as upstream regulators, all of which share the ability to function as ligands for the EGFR. Based on these observations, we hypothesize that incubation with lactic acidosis induces differentiation in CCDSk cells through activation of the EGFR signaling pathway.

### 2.9 P‐SRF and cFOS Possibly Mediate Lactic Acidosis‐Induced Integrated Stress Response in CCDSk Cells

Canonical pathway analysis predicted activation of the integrated stress response following incubation with lactic acid. This is consistent with the concept that differentiation of fibroblasts is often accompanied by a switch in the transcriptional program and that pathophysiological changes in the cellular microenvironment frequently trigger activation of the integrated stress response. Figure 6C illustrates potential upstream regulators through which lactic acidosis may mediate this response. Notably, several members of the AP‐1 transcription factor complex, including FOS, JUN, and JUNB, were prominently represented.

To assess the validity of this prediction, we examined whether lactic acidosis alters the protein expression of cFOS and its upstream regulator phosphorylated SRF (p‐SRF). The results demonstrated that lactic acidosis induces the protein expression of both cFOS and p‐SRF (Figure 6D). Based on these findings, we hypothesize that incubation with lactic acidosis induces the integrated stress response in CCDSk cells via activation of the SRF‐cFOS axis.

### 2.10 Potential Role of IL‐6 and p38‐Signaling in Lactic Acidosis‐Induced Inflammatory Response of Fibroblasts

Canonical pathway analysis predicted lactic acidosis‐induced cytokine signaling, characteristic of an inflammatory response. This is consistent with the notion that fibroblast differentiation can involve a phenotypic switch toward an inflammatory state. Moreover, Figure 6C illustrates the third pattern derived from the upstream regulator analysis. This pattern comprises upstream regulators that may mediate the lactic acidosis‐induced inflammatory response. Notably, several components of the IL‐6 signaling pathway, including IL‐6, IL‐6R, and JAK1, were enriched within this pattern. In addition, MAPK14 (p38) was predicted as an upstream regulator. To assess whether IL‐6 and p38 represent promising mediators, changes in their protein expression following treatment with lactic acidosis were analyzed by Western blot. Our results showed that lactic acidosis induced a transient increase in phosphorylated p38 after 3 h, as well as an increase in IL‐6 protein levels after 48 h (Figure 6D). These findings suggest that lactic acidosis promotes an IL‐6‐associated inflammatory response involving p38 signaling. However, a direct mechanistic connection between these pathways remains to be investigated in future studies.

## 3 Discussion

Chronic kidney disease (CKD) represents a major global health burden, and causal therapies to halt or reverse disease progression remain unavailable, highlighting the need to better understand the mechanisms driving CKD pathogenesis [1]. The present study investigated how extracellular lactic acidosis modulates the transcriptomes of human proximal tubular cells (HK‐2) and fibroblasts (CCDSk cells) to generate hypotheses regarding its potential phenotypic consequences. Although immortalized cell lines have inherent limitations, HK‐2 cells retain key features of proximal tubular epithelium, including a polarized morphology and the expression of characteristic transporters.

Whereas CCDSk cells exhibit a typical fibroblast morphology [12]. Nonetheless, validation in primary cells and in vivo models will be essential. To model pathophysiological conditions, pH 6.4 was selected, reflecting a transition into the pathological range while preserving cell viability [12]. Because lactic acidosis simultaneously lowers extracellular pH, increases lactate concentration, and raises osmotic pressure, we included distinct control groups: acidosis (hydrochloric acid, pH 6.4), lactate (20 mM, pH 7.4), osmotic control (mannitol, 20 mM, pH 7.4), lactic acidosis (20 mM, pH 6.4), and untreated control (pH 7.4). Notably, while previous studies have suggested that sodium lactate may act as a mediator of fibrotic changes during CKD, sodium lactate did not elicit transcriptomic alterations in either cell type [13]. Similarly, osmotic stress, despite being reported as a driver of proximal tubular dedifferentiation, did not induce detectable transcriptomic changes in our experiments [14]. The virtual absence of transcriptomic alterations following sodium lactate or mannitol treatment does not exclude the possibility that these treatments induce biologically relevant post‐transcriptional or post‐translational changes, including alterations in protein phosphorylation, ubiquitination, proteasomal degradation, or protein stability. Consequently, biologically relevant effects of sodium lactate or osmotic stress on intracellular signaling or cellular phenotype cannot be excluded. Consistent with this notion, sodium lactate significantly reduced the number of nuclei in proximal tubular cells, indicating that elevated lactate alone can exert biological effects despite the absence of detectable transcriptomic alterations. Indeed, both lactate and osmotic stress have previously been reported to modulate cellular function through signaling pathways that do not necessarily require de novo gene transcription. Nevertheless, it is remarkable that lactic acidosis, but not sodium lactate alone, induced extensive transcriptomic remodeling in both proximal tubular cells and fibroblasts.

The presented results demonstrate that the combination of extracellular acidosis and elevated lactate exerts a profound impact on the transcriptome of both CCDSk cells and HK‐2 cells, although the consequences of these changes are highly cell‐type‐specific.

Figure 1A demonstrates that extracellular lactate and acidosis synergistically alter the transcriptome. Whereas hydrochloric acid induced only 425 differentially expressed genes (DEGs) in proximal tubule cells, lactic acid increased this number to 3807, indicating that elevated lactate markedly amplifies the transcriptional response to extracellular acidification. This synergism was also shown for vascular smooth muscle cells by Terpe et al. [15]. Several mechanisms may account for this synergism. First, at least during the initial phase of the incubation period, an acidic extracellular milieu may enhance lactate uptake via monocarboxylate transporters (MCTs). Second, increased intracellular lactate may alter gene expression through protein lactylation, particularly histone lactylation. Third, extracellular lactate may directly activate lactate‐sensing receptors. These potential mechanisms are discussed below.

1. MCTs mediate the electroneutral cotransport of lactate and protons, and their transport direction is determined by the combined lactate and proton gradients:

$${\left[{\text{Lactate}}^{-}+H^{+}\right]}_{\mathrm{ec}}↔{\left[{\text{Lactate}}^{-}+H^{+}\right]}_{\mathrm{ic}}$$

As summarized in Table 2, MCT1 and MCT2 exhibit high affinity for lactate and are expected to be largely saturated at the extracellular lactate concentration used in this study, whereas MCT4, owing to its lower affinity, remains more dependent on extracellular lactate concentration. For all MCT isoforms, the proton gradient becomes a major determinant of the driving force under acidic conditions by shifting the equilibrium concentration of lactate and thereby governing the net direction of transport.

**TABLE 2:** Characteristics of MCTs.

Gene name	Corresponding protein	Function	Km	Transport capacity
SLC16A1	MCT1	cotransport of protons and monocarboxylates through the cell membrane	~3–5 mM	moderate
SLC16A7	MCT2		~0.5–1 mM	low
SLC16A3	MCT4		~20–35 mM	high
BSG	Basigin	Chaperone required for MCT1/MCT4 plasma membrane localization and activity		

As shown previously [12, 16], exposure to extracellular pH 6.4 decreased the intracellular pH to 6.66 in HK‐2 cells and to 6.92 in CCDSk cells. These values correspond to extracellular and intracellular free proton concentrations of 398 nM and 219 nM in HK‐2 cells, and 398 nM and 120 nM in CCDSk cells, respectively. Consequently, both cell types maintain an inward‐directed proton gradient, with extracellular proton concentrations approximately 1.8‐fold higher than intracellular concentrations in HK‐2 cells and 3.3‐fold higher in CCDSk cells.

Based on thermodynamic equilibrium, intracellular lactate concentrations of approximately 36.4 mM in HK‐2 cells and 66.2 mM in CCDSk cells would be required to abolish the inward driving force at an extracellular lactate concentration of 20 mM. Because such intracellular lactate concentrations are unlikely to be reached under our experimental conditions, the proton gradient is expected to favor net lactate influx via MCT1, MCT2, and MCT4.

Consistent with this hypothesis, Table S7 shows that lactic acid increased the expression of SLC16A1 (MCT1), SLC16A3 (MCT4), and BSG (Basigin), the essential membrane chaperone for MCT1 and MCT4, in CCDSk cells. Together, these changes may further facilitate lactate uptake in CCDSk cells. For HK‐2 cells, no lactic acid‐induced alteration of MCTs or BSG was measureable. (Table S6).

2 Increasing evidence indicates that intracellular lactate functions not only as a metabolic intermediate but also as an epigenetic regulator. Lactate can modify lysine residues of proteins, particularly histones, through lysine lactylation, thereby altering transcriptional programs. Several studies have linked histone lactylation to profibrotic signaling in renal epithelial cells [17]. Furthermore, histone lactylation has been shown to promote IL‐6‐dependent inflammatory responses [18] and to regulate both inflammatory signaling and metabolic reprogramming during kidney repair following acute kidney injury [19, 20].

Together, these findings support the hypothesis that enhanced intracellular lactate contributes to disease progression through lactylation‐dependent transcriptional reprogramming. Future studies should therefore determine whether lactic acidosis increases histone lactylation in HK‐2 cells and CCDSk cells. Moreover, the potential involvement of histone lactylation in the lactic acid‐induced alterations could be investigated by pharmacological inhibition of p300/CBP using A‐485. As p300/CBP can catalyze the transfer of lactyl groups to histones, inhibition with A‐485 may help determine whether p300/CBP‐dependent histone lactylation contributes to the observed cellular responses. However, because p300/CBP also regulates histone acetylation and transcriptional activity, complementary analyses of histone lactylation would be required to confirm a lactylation‐dependent mechanism.

3 In addition to intracellular lactylation, extracellular lactate may regulate gene expression by activating cell surface lactate receptors. The best‐characterized receptor is hydroxycarboxylic acid receptor 1 (HCAR1/GPR81), a G protein‐coupled receptor that modulates fibrosis, inflammation, and immune responses through cAMP/PKA‐ and β‐arrestin‐dependent signaling pathways [21]. Although HCAR1 transcripts were detected at low levels in both cell types, its contribution to the observed transcriptional responses cannot be excluded. Future studies should therefore investigate the signaling pathways activated during prolonged lactic acidosis and identify the upstream sensors responsible for these responses.

HK‐2 cells displayed a subset of DEGs directly triggered by acidosis. Further analysis of this subgroup generated the hypothesis that acidosis induces cell cycle arrest in HK‐2 cells. This was experimentally supported by BrdU incorporation assays and cell cycle analyses, which consistently indicated a sustained G1 arrest accompanied by reduced DNA synthesis. Such a response is consistent with the established concept that acute injury often provokes a transient cell cycle arrest to allow DNA repair [22, 23]. Although, cell death can be induced when repair mechanisms fail [24], viability markers provided no evidence for acidosis‐induced necrosis or apoptosis we assessed apoptosis, and necrosis under acidic conditions. Neither was detected, indicating preserved cell viability (Figure S2). Given that proximal tubular cells generally display low proliferative activity under physiological conditions but accelerate proliferation in response to injury [1, 25], disruption of this repair mechanism may represent a key event in the transition from acute kidney injury (AKI) to CKD [9]. Considering that up to one‐third of AKI patients develop CKD within one year, this process is of major clinical relevance [26]. While the link between acidosis and cell cycle arrest has been well documented in tumor cells [27, 28], our study provides, to our knowledge, the first evidence for such a relationship in proximal tubular cells. Based on our RNA‐seq data, upstream regulator analysis enabled the formulation of promising hypotheses about how acidosis mediates the cell‐cycle arrest. Our upstream regulator analysis suggests that acidosis may induce cell‐cycle arrest via activation of CDKN2A. This hypothesis could be addressed by pharmacologically inhibiting CDKN2A and examining whether such interventions modulate acidosis‐induced cell cycle arrest. As shown in Figure S9A additional to cell cycle arrest, canonical pathway analysis predicted that acidosis inhibits lipid and cholesterol metabolism. This finding was supported by upstream regulator analysis, which predicted inhibition of RXRA, SCAP, SREBF1, and SREBF2 (Figure S9B). In proximal tubular cells, this pattern indicates a coordinated suppression of lipid and cholesterol homeostasis, which may compromise membrane integrity, metabolic flexibility, and adaptive stress responses [29, 30, 31]. Cell‐cycle arrest as well as disturbed lipid metabolism following acute injury have been described as a maladaptive repair process, rendering tubular cells more vulnerable to subsequent insults [32]. It is therefore conceivable that the acidosis‐induced cell cycle arrest measured in our study and a possible disturbance in lipid metabolism provide the foundation for the synergistic profibrotic effect of lactic acidosis. Fibrosis is a hallmark of progressive CKD and is charactarized by remodeling and accumulation of the extracellular matrix and thereby leading to loss of organ function [33]. Our upstream regulator analyses predicted CD44 and TGFBR2 as potential mediators of the lactic acidosis‐induced fibrotic response in HK‐2 cells. Both proteins are membrane‐bound receptors that can interact and transduce extracellular stimuli, such as acidosis, into profibrotic signaling responses [34, 35]. In addition, the analysis predicted an increased activity of RRAS2, MAP2K1 (MEK1), and MAPK3 (ERK2), which are classical downstream components of CD44 signaling, as well as SMAD3 and SMAD4, canonical downstream effectors of TGFBR1 signaling [36, 37]. In our study, we experimentally tested the prediction that the IL‐6‐JAK1/STAT3 axis contributes to this response. Our results showed that lactic acidosis induced increased STAT3 phosphorylation independently of IL‐6 expression. Notably, STAT3 is also a known downstream target of CD44, and its activation in this context has been implicated in the development of fibrosis [38]. Future studies will need to address (i) whether lactic acidosis indeed induces a fibrotic response in HK‐2 cells and (ii) whether such a response depends on the CD44‐STAT3 axis and TGFBR1 signaling. In contrast to HK‐2 cells, canonical pathway analysis in CCDSk cells predicted an induction of cell‐cycle‐related pathways (actin signaling, CDK5 signaling, G2/M‐phase, ephrin receptor signaling, growth factor signaling, TP53‐ and RUNX1‐signaling). Additionally, the cell‐cycle analysis revealed that acidosis induced a transient G1 arrest at 24 h, which was resolved by 48 h. Upstream regulator analysis predicted that lactic acid‐induced changes are mediated by growth factors, example EGFR. Additional experiments confirmed that lactic acid‐induced EGFR phosphorylation in CCDSk cells after 48 h (Figure S4). Fibroblast differentiation is associated with transcriptional reprogramming that enables adaptation to changes in the microenvironment. In the present study, enrichment of AP‐1 complex members among the predicted upstream regulators, together with the measured lactic acid‐induced protein expression of cFos and phosphorylation of its upstream regulator SRF, supports activation of the SRF‐cFos axis. To investigate how lactic acidosis induces the P‐SRF and cFos expression, well‐known upstream regulators—EGFR, ERK1/2, and Rho/ROCK signaling—were pharmacologically inhibited as described in the Supplementary Methods. The results revealed that lactic acid‐induced phospho‐SRF expression depended, at least in part, on ERK1/2 and Rho/ROCK signaling, whereas the effects of EGFR inhibition were inconsistent (Figure S6). Notably, ERK1/2 phosphorylation itself was not altered by lactic acid, suggesting that ERK1/2 plays a permissive rather than an inducible role (Figure S3). In contrast, lactic acid‐induced cFos expression required both EGFR and ERK1/2 signaling, whereas inhibition of the Rho/ROCK pathway had no effect (Figure S7). Following kidney injury, resident fibroblasts migrate toward the site of injury and support tissue repair [39]. In contrast, during long‐lasting stress, fibroblasts undergo a phenotypic switch characterized by a transient cell‐cycle re‐entry and activation of the integrated stress response [40, 41]. In fibroblasts, this process is frequently associated with differentiation whereby, fibroblasts can adopt multiple functional phenotypes, and in the context of CKD they initially shift toward an inflammatory phenotype characterized by enhanced proliferation, cytokine secretion, and reduced extracellular matrix synthesis [42, 43]. Consistent with this concept, canonical pathway analysis predicted activation of lactic acidosis‐induced cytokine signaling. Moreover, the upstream regulator analysis combined with the following Western blot analysis suggested that lactic acidosis mediates an IL‐6‐driven inflammatory response in CCDSk cells. Figure S5 provides initial evidence that lactic acid induces the IL‐6 expression via ERK1/2 signaling, whereas EGFR as well as Rho/Rock signaling were not involved.

## 4 Material and Methods

### 4.1 Cell Culture

Normal human kidney epithelial cells (HK‐2, ATCC CRL‐2190) and normal human fibroblasts (CCD‐1092Sk, ATCC CRL‐2114) were grown in DMEM medium (P04‐01504, Pan Biotech, Aidenbach, Germany) supplemented with 10% fetal calf serum (FCS, P30‐3031, Pan Biotech, Aidenbach, Germany) and 24 mM NaHCO_3_. Cells were kept at 37°C under a humidified 5% CO_2_ atmosphere and subcultured once per week before confluence.

### 4.2 Experimental Setup

#### 4.2.1 Cell Culture for RNA Sequencing and Western Blot Analysis

Epithelial cells, such as HK‐2 cells, attach to the substrate via their basolateral membrane, whereas the apical membrane faces toward the tubular lumen. Thus, in vivo, tubular epithelial cells are separated from the interstitial space that harbors fibroblasts. When using 6‐well plates, HK‐2 cells were grown on permeable filter inserts (pore size 0.4 μm, 83.3930.101 Sarstedt, Nürmbrecht, Germany), and CCDSk cells were grown in 6‐well plates (83.3920 Sarstedt, Nürmbrecht, Germany). As shown in Figure S1, after 24 h of serum starvation, the cells were incubated for 48 h without serum for RNA‐Seq or Western blot (A) or with 10% FCS for cell‐cycle analysis (B).

Both groups were incubated with control medium (pH 7.4), medium containing 20 mM hydrochloric acid (pH 6.4) (1.09057.1000, Sigma‐Aldrich, Darmstadt, Germany), 20 mM lactic acid (pH 6.4) (L1750, Sigma‐Aldrich, Darmstadt, Germany), 20 mM sodium lactate (pH 7.4) (L‐7022, Sigma‐Aldrich, Darmstadt, Germany), or 20 mM mannitol (pH 7.4) (M‐4125, Sigma‐Aldrich, Darmstadt, Germany). The nominal bicarbonate concentration of the culture medium at pH 7.4 was 24 mM, whereas according to the Henderson–Hasselbalch equation at pH 6.4 it decreased to 2.5 mM. Because lactic acid is a weak acid (pK_a_ ≈3.86), it exists in equilibrium between its protonated (lactic acid) and deprotonated (lactate) forms, with their relative abundance determined by the extracellular pH according to the Henderson–Hasselbalch equation. Consequently, at both pH 6.4 and pH 7.4, > 99% of the 20 mM total lactate is present as the lactate anion, whereas only a small fraction remains protonated (approximately 0.06 mM at pH 6.4 and 0.006 mM at pH 7.4). Thus, the lactic acid condition combines extracellular acidification with an elevated lactate concentration, whereas the sodium lactate condition provides the same lactate concentration under physiological pH. This experimental design allows the effects of reduced pH, elevated lactate concentration, and their combination to be distinguished.

#### 4.2.2 Measurement of Extracellular pH (pHe)

To determine the basal pH of the culture medium, cell‐free medium was incubated for 48 h in a 12‐well plate under standard culture conditions (37°C, 5% CO_2_). HK‐2 cells and CCDSk cells were incubated after 24 h of serum starvation for 48 h with either control medium (pH 7.4) or lactic acid‐containing medium adjusted to pH 6.4 (*n* = 8–12 wells per condition). Following incubation, 400 μL of culture medium was collected from each well and transferred into 2‐mL reaction tubes for pH measurement. The pH was measured using a calibrated pH microelectrode (InLab Solids Go‐ISM Mettler Toledo, Gießen, Germany). Prior to each experiment, the electrode was calibrated using standard buffer solutions at pH 7.0 and pH 4.0 according to the manufacturer's instructions. To minimize changes in pH caused by equilibration with atmospheric CO_2_, the pH electrode was immersed in the sample, the incubator was immediately closed, and the pH was measured under a controlled atmosphere of 5% CO_2_ at 37°C.

### 4.3 RNA‐Isolation

The total RNA was isolated with BlueZol Reagent (003980801, Serva, Heidelberg, Germany) as described in the user manual. To remove possible genomic DNA contamination, the “rigorous DNase treatment” protocol of the “Turbo DNase‐free kit” (Invitrogen, Life Technologies, Schwerte, Germany) was used. Afterwards, the samples were cleaned by ethanol precipitation (with 3 M sodium acetate, glycogen, and 100% ethanol). Alternative purification columns were used to according to the manufacturer's protocol (Monarch RNA Purification Columns, T2007L, New England Biolabs, Frankfurt am Main, Germany). The RNA concentration as well as the quality was measured using a 2100 Bioanalyzer System (Agilent Technologies, Germany). The threshold for RNA Integrity Number (RIN) was ≥ 7 (with 10 as the maximal possible value).

### 4.4 RNA‐Sequencing

In preparation for RNA sequencing HK‐2 cells and CCDSk cells had a period of serum starvation (24 h) and were incubated for a further 48 h with control medium (pH 7.4), medium containing hydrochloric acid (pH 6.4), lactic acid (pH 6.4), sodium lactate (pH 7.4) and mannitol (pH 7.4). RNA sequencing was performed for five to six biological replicates per cell type and culture condition (see Table S2 for details). Novogene Co. Ltd. (Cambridge, United Kingdom) performed the sequencing library preparation (pol(A) enrichment) and the paired‐end sequencing (2 × 150 bp) with a NovaSeq6000 Illumina system. Adaptor clipping and data quality control were provided by the service company. Read mapping to the human genome hg38 was done with HISAT2 [44] (version 2.1.0), and featureCounts [45] (version 2.0.0) was used to count mapped reads. BiomaRt [46] was used for gene annotation.

### 4.5 Differential Expression Analysis

Figure 7 summarizes the analysis pipeline applied for RNA sequencing data analysis. The analysis was performed using edgeR [47] (version 3.30.3) and DESeq2 [48] (version 1.28.1). Genes with sufficient read counts for inclusion in the statistical analyses were filtered using the filterByExpr edgeR function and the independent filtering parameter (*α* = 0.05) in DESeq2. Normalization factors were calculated with the “trimmed mean of M value” (TMM) method in edgeR analysis. Significantly “differentially expressed genes” (DEG) were defined as genes with a false discovery rate (FDR) below 0.05 in both DESeq2 and edgeR outputs (overlap of the respective results), with at least 3 FPM on average in one of the sample groups considered for a given comparison and with |log_2_FC| ≥ 0.5 (threshold based on the inherent variation in control samples: threshold = 3 × [average coefficient of variation] from all RNAs).

### 4.6 Gene Ontology Enrichment Analysis

Gene Ontology (GO) term enrichment was performed with the webserver g:Profiler [49] (version: e113‐eg59_p19_f6a03c19). The lists of genes differentially expressed by (i) lactic acid incubation or (ii) hydrochloric acid and lactic acid (overlap of the results obtained for the single incubations) were used as input. The first list contained all decreased DEGs and the second list contained all increased DEGs. For subsequent interpretation, only Gene Ontology (GO) terms belonging to the categories Biological Process (BP), Molecular Function (MF), and Cellular Component (CC) were considered, as these ontologies provide a structured and well‐established framework for the biological interpretation of the identified genes. GO terms were defined as significantly enriched if the following criteria were met: adjusted *p*‐value < 1E‐09 and enrichment score E ≥ 3, (with E = (intersection size/query)/(term size/effective domain size)).

### 4.7 Ingenuity Pathway Analysis

#### 4.7.1 Standard Procedure

QIAGEN Ingenuity Pathway Analysis [50] (IPA—https://digitalinsights.qiagen.com/IPA) was used for further investigation. Especially the “Canonical pathway analysis” (Identification of most significantly affected pathways and those that are predicted to be activated or inhibited by changes in gene regulation) and “Upstream Regulator” (prediction of regulators of the observed gene regulation) tools were used. The Ensembl identifiers of the identified DEGs were incorporated into the software database. Moreover, the log_2_FC calculated by DESeq2 was included for each gene. IPA used these values to predict whether enriched canonical pathways or upstream regulators were rather activated or inactivated (see Tables S8 and S10). This information is returned as a *Z*‐score = ($\frac{\left(N^{+}-N^{-}\right)}{N^{0.5}}$), *N* ^+^ and *N* ^−^, the numbers of genes with direction of expression changes consistent with or not consistent with the knowledge base, respectively, and the total number of considered genes. A positive *Z*‐score suggests an activation, while a negative *Z*‐score suggests an inactivation of, example a signaling pathway. The predicted canonical pathways were filtered for adjusted (Benjamini–Hochberg) *p*‐value < 0.01 and |*Z*‐score| ≥ 3. For the comparative analysis between hydrochloric acid and lactic acid in HK‐2 cells, an absolute *Z*‐score of 3 or greater should be reached in at least one group (see Table S8).

#### 4.7.2 Refined Analysis of Predicted Upstream Regulators

The upstream regulator analysis initially yielded a large number of predicted regulators (124 for HK‐2 cells and 584 for CCDSk cells), necessitating further refinement. Therefore, a stringent multistep analysis pipeline was applied (Figure 7B). Predicted upstream regulators were first filtered for statistical robustness using an adjusted (Benjamini–Hochberg) *p*‐value < 0.01 and an absolute *Z*‐score ≥ 3. The remaining regulators were then hierarchically classified according to their position within the canonical cellular signal transduction cascade, from extracellular stimuli to transcriptional regulation (Figure 7C), based on molecule type assignments provided by Ingenuity Pathway Analysis (IPA).

Regulators corresponding to biological drugs, chemical drugs, or chemical reagents were excluded, as they do not represent physiologically plausible mediators of acid‐induced effects.

To prioritize biologically relevant candidates, an evidence‐based classification was applied by comparing measured gene expression changes (log_2_FC) with predicted activity states (*Z*‐scores). Upstream regulators with concordant directions of log_2_FC and *Z*‐score were assigned the highest confidence (evidence level III). Regulators with strong predicted activity (|*Z*‐score| > 3) but no detectable expression change were classified as evidence level II, whereas regulators with discordant log_2_FC and *Z*‐score directions were excluded (evidence level I).

The final lists of filtered upstream regulators for HK‐2 cells and CCDSk cells are provided in Tables S8 and S9. These regulators were subsequently analyzed using the STRING database (version 12.0) to construct protein–protein interaction networks and identify densely connected functional modules via built‐in clustering algorithms. Functionally related clusters were extracted for downstream analyses.

### 4.8 Cell Cycle Analysis

#### 4.8.1 DAPI Staining

After the incubation duration, cells were fixed with 4% formaldehyde for 60 min at room temperature. All nuclei were stained with 2 mg/L DAPI (4′,6‐diamidino‐2‐phenylindole, DAPI, D1306, Invitrogen, Schwerte, Germany) for 10 min at room temperature. After each step, three washing steps were performed using 1 × PBS (Table S1). Subsequently, DAPI fluorescence (Ex = 358 nm, Em = 461 nm; blue channel) was measured using an imaging multimode reader (Cytation 3, Agilent, Waldbronn, Germany) and analyzed with Gen5 image analysis software (version 3.08).

#### 4.8.2 Cell Cycle Analysis

For cell cycle analysis, the number and area of nuclei as well as the DAPI intensity per nucleus were quantified using Gen5 software (BioTek). For DAPI intensity measurements, a background signal (~1000 arbitrary units) was estimated and subtracted. In addition, thresholds for cell size and nuclear area were applied (object size: 10–40 μm; nuclear area > 150 μm^2^). Based on these parameters, the DAPI integral per cell was calculated (nuclear area × [DAPI intensity − background]) as an estimate of the DNA content per cell.

**FIGURE 8:**
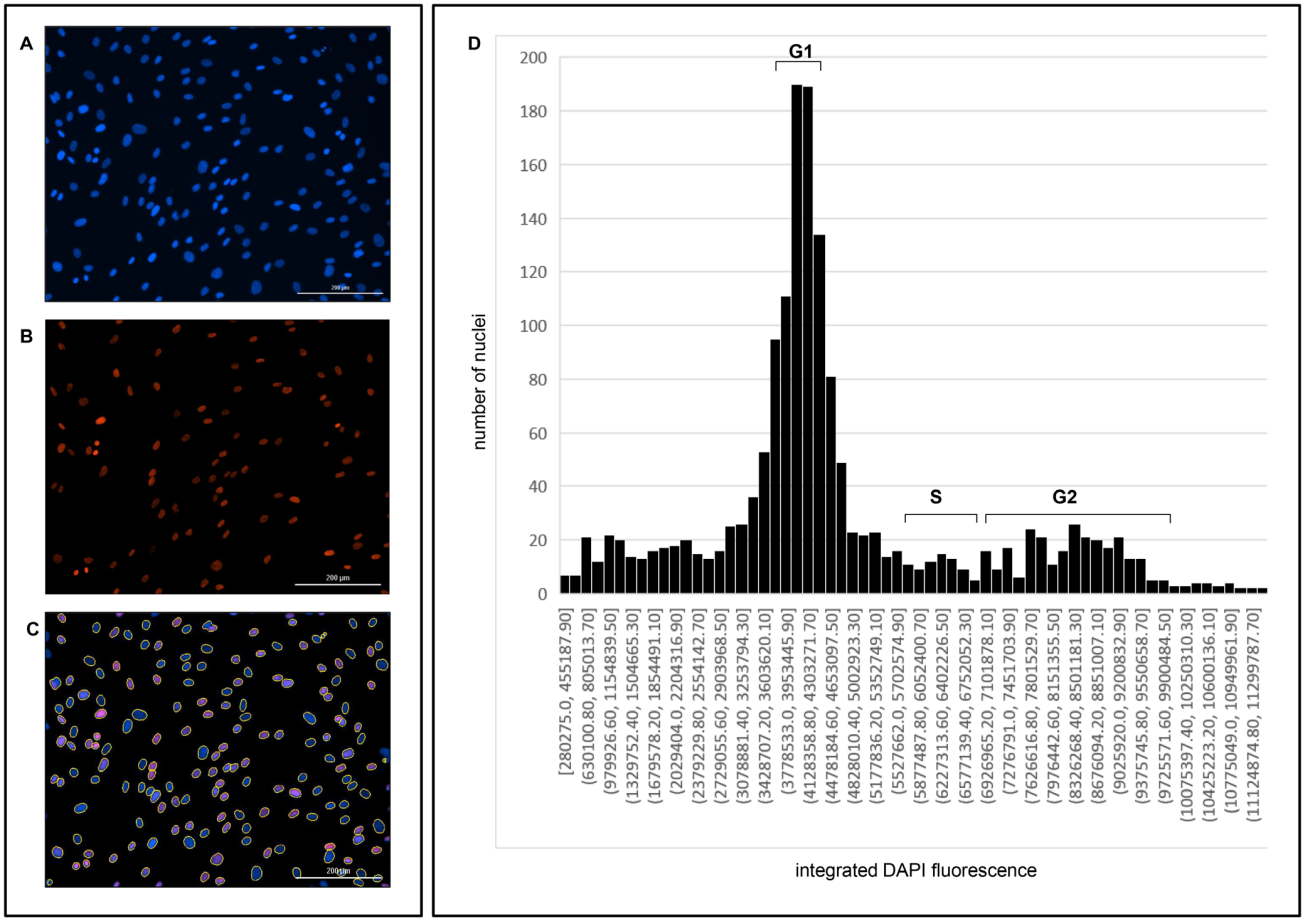
Example of blue‐ (A) and red‐stained nuclei (B). Overlap of the blue and red channels with an identification line in yellow (C). (D) Histogram showing the distribution of the DAPI integral, which was used as a surrogate marker for DNA content. Cells in G2 phase exhibit approximately twice the DNA content of G1 phase cells.

As shown in Figure 8D, the distribution of per‐cell DAPI integrals was plotted as a histogram to determine the allocation of cells to different cell cycle phases. Lower and upper thresholds were defined to identify cells in G1 phase. Assuming a doubling of DNA content in G2 phase compared to G1 phase, the G1 thresholds were multiplied by two to define G2‐phase cells. Cells with intermediate DAPI integrals were classified as being in S phase. The relative proportion of cells in G1 and G2 phases was quantified by calculating the G2/G1 ratio.

### 4.9 BrdU‐ELISA

#### 4.9.1 Bromodeoxyuridine Incorporation

To estimate DNA synthesis over the entire incubation period, a BrdU ELISA was performed. Cells were incubated with 10 μM bromodeoxyuridine (BrdU; B5002, Sigma‐Aldrich, Darmstadt, Germany), a thymidine analogue, throughout the whole incubation period. During DNA synthesis (S phase and G2 phase), BrdU is incorporated into newly synthesized DNA. After the incubation period, cells were fixed with 4% formaldehyde as stated in 4.8.1. The DNA was denatured with 2 N hydrochloric acid for 30 min, and cells were blocked with permeabilization buffer (Table S1) containing 10% FCS for 1 h. BrdU antibody (Table 3) was applied overnight, followed by a fluorescence‐conjugated secondary antibody (Table 3). The second column of the plate (wells B2–G2) contained cells but was not incubated with the BrdU primary antibody; instead, cells were incubated only with the fluorescent secondary antibody. This column was used to determine the background fluorescence of the secondary antibody. All nuclei were counterstained with 2 mg/L DAPI for 10 min at room temperature. After each step, 3 washing steps were performed using 1 × PBS (Table S3). Subsequently, the DAPI (wavelength: Ex = 358 nm, Em = 461 nm) (blue channel) and BrdU (Ex = 586 nm, Em = 605 nm) signals were measured by an imaging multimode reader (Cytation 3, Agilent, Waldbronn, Germany).

**TABLE 3:** Antibodies.

Target	Company	Order number	Host	Dilution
Actin, ß—	Cell Signaling, Danvers, USA	3700	Mouse	1:1000
BrdU (Bromodeoxyuridine)	Becton Dickinson, Heidelberg, Germany	347 580	Mouse	1:200
cFOS	Cell Signaling, Danvers, USA	2250	Rabbit	1:1000
IL‐6	Cell Signaling, Danvers, USA	12 153	Rabbit	1:500
Jak1	Cell Signaling, Danvers, USA	3332	Rabbit	1:1000
Phospho‐JAK1 (Tyr1034/1035)	Cell Signaling, Danvers, USA	74 129	Rabbit	1:1000
JAK2	Cell Signaling, Danvers, USA	74 987	Rabbit	1:1000
Phospho‐JAK2 (Tyr1007/1008)	Cell Signaling, Danvers, USA	3776	Rabbit	1:1000
MAPK, p44/42‐ (ERK1/2)	Cell Signaling, Danvers, USA	4696	Mouse	1:1000
MAPK, Phospho‐p44/p42‐, (Thr202/Tyr204)	Cell Signaling, Danvers, USA	9101	Rabbit	1:1000
SRF	Cell Signaling, Danvers, USA	5147	Rabbit	1:1000
Phospho‐SRF (Ser103)	Cell Signaling, Danvers, USA	4261	Rabbit	1:1000
STAT3	Cell Signaling, Danvers, USA	9139	Rabbit	1:1000
Phospho‐STAT3 (Tyr705)	Cell Signaling, Danvers, USA	9145	Rabbit	1:1000
Anti‐Rabbit IgG HRP	Cell Signaling, Danvers, USA	7074	Goat	1:2000
Anti‐Mouse Alexa Fluor 568	Invitrogen, Schwerte, Germany	A10037	Donkey	1:200

#### 4.9.2 Quantification of DNA Synthesis

To quantify DNA synthesis per well, the mean BrdU intensity was measured and used to calculate the BrdU integral per well (DAPI area × [mean BrdU—blank]). In addition, the number of BrdU‐positive cells was determined. For this purpose, a BrdU intensity threshold was applied, and the number of red‐stained nuclei was counted (See Figure 8C).

### 4.10 Western Blot

Western blot was performed using cell lysates. Cells were lysed in 50 μL of ice‐cold RIPA buffer (Table S1). Afterwards, cells were centrifuged at 14,000 g for 10 min (4°C) and filled up with 16.6% of the total volume of 6 × Laemmli buffer (Table S1). Before loading the gel, samples were heated to 95°C for 5 min. Proteins were separated by 10% (SRF, JAK1/2, STAT3, cFOS) or 14% (IL‐6) sodium dodecyl sulfate–polyacrylamide gel electrophoresis (SDS‐PAGE) and transferred onto nitrocellulose membranes. All proteins on the nitrocellulose membranes were stained with Ponceau S solution (AppliChem GmbH, Darmstadt, Germany) and detected with the Bio‐Rad ChemiDoc XRS gel documentation system (Bio‐Rad Laboratories GmbH, Feldkirchen, Germany). After staining, the membrane was blocked with 5% nonfat dry milk powder in TRIS‐buffered saline (TBS) (Table S1) and incubated with primary antibodies (Table 3) diluted in 5% bovine serum albumin (BSA) in TBS + 0.1% Tween 20 overnight at 4°C. After removing excess primary antibody and washing the membranes, a secondary antibody coupled to horseradish peroxidase (Table 3) was diluted 1:2000 in 5% nonfat dry milk powder in TBS with Tween 20 and added to the membranes. After removal of the secondary antibody solution, three wash steps in TBS with Tween 20 were performed for 5 min/wash step. Finally, the membrane was incubated for 5 min with Clarity Western ECL Substrate (Bio‐Rad, Munich, Germany), and the peroxidase activity‐based light emission was recorded by an imaging system (Image Quant LAS4000, GE Healthcare, Buckinghamshire, UK). The density of the total protein, made visible with Ponceau S solution, and protein target bands were quantified using Image Lab Software 6.1 and used for normalization (Bio‐Rad, Munich, Germany, 2020).

### 4.11 Measurement of Total Cellular Protein by BCA

Cells were lysed in 100 μL MOPS–Triton buffer (for enzyme measurement) or 50 μl RIPA (for Western blot) (Table S1) and centrifuged at 13000 × g for 10 min. The supernatant was used for protein quantification. For each sample, 5 μL of lysate was incubated for 30 min with 200 μL reaction solution (50 parts BCA reagent (23 222, Pierce, Darmstadt, Germany) and 1 part copper sulfate). Absorbance was measured at 562 nm. A calibration curve was prepared in parallel using defined bovine serum albumin (BSA) standards (100, 300, 500, and 700 mg/L) as well as lysis buffer and solvent blanks. Protein concentrations in the samples were calculated according to the Lambert–Beer law.

### 4.12 Data Analysis

Differential expression analysis for results from RNA‐sequencing was performed using the gold standard tools DESeq2 and edgeR. Statistical significance was determined by an unpaired Student's *t*‐test or one‐way ANOVA with Holm–Sidak as a post hoc test for normally distributed data. For data without a normal distribution, rank sum or ANOVA on ranks with Dunn's as a post hoc test were applied. The comparison for the ANOVA‐tests was performed with the associated control group. Differences were considered statistically significant when *p* < 0.05. To generate Venn diagrams, the interactive tool “Venny” 2.1 was used (https://bioinfogp.cnb.csic.es/tools/venny/index.html).

### 4.13 Language and Writing Assistance

The language, phrasing, and wording of this manuscript were reviewed and refined with the assistance of an AI‐based language model (OpenAI, ChatGPT 5). It was utilized to check the grammar, clarity, and consistency of the text, as well as to enhance the overall readability. The final version of the manuscript was reviewed and approved by the authors to ensure that it accurately reflects their intended meaning and scholarly standards.

## 5 Conclusion

In summary, our data predict that extracellular lactic acidosis is accompanied by profound phenotypic changes in HK‐2 cells (proximal tubular cells) and CCDSk cells (fibroblasts). These alterations appear to be rooted in an acid‐induced cell‐cycle arrest common to both cell types. While the combination of acidosis and lactate promoted a fibrotic response in HK‐2 cells, CCDSk cells instead adopted an inflammatory phenotype. Our findings provide the first evidence that local extracellular acidosis can act as an initiator of a microenvironment‐driven cellular response. Extracellular acidification may serve as a “door‐opener” for subsequent alterations mediated by the complex tubulo‐interstitial milieu, and if confirmed in vivo, this mechanism would offer important new insights into the pathophysiological processes driving CKD progression.

## Author Contributions

**Lea Schaude:** investigation. **Isabelle Görlich:** investigation. **Natalie Schröder:** investigation. **Marie‐Christin Schulz:** conceptualization, investigation, writing – original draft, methodology, visualization, project administration. **Virginie Dubourg:** writing – original draft, methodology, writing – review and editing, formal analysis, software. **Eva Janßen:** investigation. **Michael Kopf:** investigation, methodology. **Michael Gekle:** conceptualization, investigation, funding acquisition, writing – original draft, data curation, supervision, resources, project administration, validation. **Stefanie Ruhs:** investigation, formal analysis, validation.

## Funding

The authors have nothing to report.

## Ethics Statement

The authors have nothing to report.

## Consent

The authors have nothing to report.

## Conflicts of Interest

The authors declare no conflicts of interest.

## Supporting information


**Figure S1:** Scheme of the incubation protocol for RNA sequencing (A) and cell cycle analysis (B).
**Figure S2:** Total cellular protein amount, caspase‐3 activity and caspase‐3 release in HK‐2 cells (A) and CCDSk (B) after 48 h incubation.
**Figure S3:** Western blot analysis of phosphorylated ERK1/2 and total ERK1/2 in CCDSk after incubation with lactic acid.
**Figure S4:** Western blot analysis of phosphorylated EGFR and total EGFR in CCDSk after incubation with lactic acid.
**Figure S5:** Western blot analysis of IL‐6 in CCDSk after incubation with lactic acid, inhibition of EGFR activation (AG1478), inhibition of ERK1/2 activation (U0126) and inhibition of Rho/ROCK pathway (Y‐27632).
**Figure S6:** Western Blot analysis of phosphorylated SRF in CCDSk after incubation with lactic acid, inhibition of EGFR activation (AG1478), inhibition of ERK1/2 activation (U0126) and inhibition of Rho/ROCK pathway (Y‐27632).
**Figure S7:** Western blot analysis of c‐Fos in CCDSk after incubation with lactic acid, inhibition of EGFR activation (AG1478), inhibition of ERK1/2 activation (U0126) and inhibition of Rho/ROCK pathway (Y‐27632).
**Figure S8:** Overlay histogram illustrating the distribution of DEGs in CCDSk after incubation with HCl (red) and lactic acid (blue).
**Figure S9:** Comparative analysis of canonical pathway (A) and upstream regulators (B) analysis by IPA with focus on lipid metabolism.
**Table S1:** Composition of the buffers and solutions used.
**Table S2:** Number of replicates for RNA sequencing.
**Table S3:** Filter parameters for differentially expressed genes.
**Table S4:** List of protein coding DEGs of separately analyzed HCl‐treated CCDSk.
**Table S5:** Enriched GO terms in CCDSk after incubation with HCl.
**Table S6:** FPM of monocarboxylate transporters and Basigin in HK‐2 cells.
**Table S7:** FPM of monocarboxylate transporters and Basigin in CCDSk cells.
**Table S8:** Overview of the comparative analysis of canonical pathway analysis obtained for genes regulated in HK‐2 cells or CCDSk cells after incubation with HCl or lactic acid. The red field marks a predicted activation (Z‐score > 3) and the blue field marks a predicted inhibition (Z‐score < −3).
**Table S9:** List of enriched GO terms for upregulated DEGs in CCDSk after incubation with lactic acid.
**Table S10:** Predicted acidosis‐induced canonical pathways in CCDSk.
**Table S11:** Filtered list of predicted upstream regulators for HK‐2 cells after incubation with lactic acid for 48 h.
**Table S12:** Filtered list of predicted upstream regulators for CCDSk cells after incubation with lactic acid for 48 h.
**Table S13:** Local network clusters of upstream regulators from HK‐2 cells identified by STRING analysis.
**Table S14:** Local network clusters of upstream regulators from CCDSk cells identified by STRING analysis.

## Data Availability

Data available on request from the authorsAdditionally, raw RNA sequencing data are publicly available in the Gene Expression Omnibus (GEO) database (https://www.ncbi.nlm.nih.gov/geo). GEO accession number: GSE220788 and GSE319482.
