# Supplementary material for: An Acidic Tubulointerstitial Microenvironment Delays Cell‐Cycle Progression in Proximal Tubule Cells and Fibroblasts and Synergizes With Lactate to Drive Phenotypic Alterations
Source: Acta Physiol (Oxf). 2026 Sep 7;242(10):e70302. doi: 10.1111/apha.70302 (PMC13548072; doi:10.1111/apha.70302)
Supplement: Supplementary file 1 [file APHA-242-e70302-s001.docx]

**Table of content**

Supplementary figure 1 Scheme of the incubation protocol for RNA-Sequencing (A) and cell cycle analysis (B).

Supplementary figure 2 Total cellular protein amount, caspase 3 activity and caspase 3 release in HK-2 cells (A) and CCDSk (B) after 48 h incubation.

Supplementary figure 3 Western Blot analysis of phosphorylated ERK1/2 and total ERK1/2 in CCDSk after incubation with lactic acid.

Supplementary figure 4 Western Blot analysis of phosphorylated EGFR and total EGFR in CCDSk after incubation with lactic acid.

Supplementary figure 5 Western Blot analysis of IL-6 in CCDSk after incubation with lactic acid, inhibition of EGFR-activation (AG1478), inhibition of ERK1/2 activation (U0126) and inhibition of Rho/Rock pathway (Y-27632).

Supplementary figure 6 Western Blot analysis of phosphorylation SRF in CCDSk after incubation with lactic acid, inhibition of EGFR-activation (AG1478), inhibition of ERK1/2 activation (U0126) and inhibition of Rho/Rock pathway (Y-27632).

Supplementary figure 7 Western Blot analysis of c-Fos in CCDSk after incubation with lactic acid, inhibition of EGFR-activation (AG1478), inhibition of ERK1/2 activation (U0126) and inhibition of Rho/Rock pathway (Y-27632).

Supplementary figure 8 Overlay histogram illustrating the distribution of DEGs in CCDSk after incubation with HCl (red) and lactic acid (blue)

Supplementary figure 9 Comparative analysis of canonical pathway (A) and upstream regulators (B) analysis by IPA with focus on lipid metabolism.

Supplementary Table 1 Composition of used buffers and solutions

Supplementary Table 2 Number of replicates for RNA-Sequencing

Supplementary Table 3 Filter parameter for differentially expressed genes

Supplementary Table 4 List of protein coding DEGs of separate analysed HCl-treated CCDSk.

Supplementary Table 5 Enriched GO terms in CCDSk after incubation with HCl

Supplementary Table 6 FPM of Monocarboxylate transporters and Basigin in HK-2 cells.

Supplementary Table 7 FPM of Monocarboxylate transporters and Basigin in CCDSk cells.

Supplementary Table 8 Overview over the comparative analysis of canonical pathway analysis obtained for genes regulated in HK-2 cells or CCDSk cells after incubation with HCl or lactic acid. Red field marks a predicted activation (z-score > 3) and the blue field marks a predicted inhibition (z-score < -3).

Supplementary Table 9 List of enriched GO terms for upregulated DEGs in CCDSk after incubation with lactic acid.

Supplementary Table 10 Predicted acidosis-induced canonical pathways in CCDSk

Supplementary Table 11 Filtered list of predicted upstream regulators for HK-2, after incubation with lactic acid for 48 h.

Supplementary Table 12 Filtered list of predicted upstream regulators for CCDSk, after incubation with lactic acid for 48 h.

Supplementary Table 13 Local network clusters of upstream regulators from HK-2 cells identified by STRING analysis

Supplementary Table 14 Local network clusters of upstream regulators from CCDSk cells identified by STRING analysis

Supplementary figure 1 Scheme of the incubation protocol for RNA-Sequencing (A) and cell cycle analysis (B).

Measurement of Caspase-3 activity assay

Protein fractions of the cells were obtained after 30 min incubation on ice with 100 µl cell lysis buffer and used to determine caspase-3 activity. 60 µl of the sample were incubated with 60 µl of Caspase-reaction buffer (supplementary Table 3) and 42 µM DEVD-AFC (end concentration) for 90 min at 37°C. Fluorescence of the cleaved product AFC was measured with a plate reader (Infinite M200, Tecan) at 400 nm excitation and 505 nm emission wavelengths. Cleaved AFC was quantified using a calibration curve with known AFC concentrations and normalised to the total amount of protein contained in the sample (determined by BCA assay 4.11.1).

Measurement of Caspase-3 release

To obtain information about necrosis, the release of caspase-3 was determined. Therefore, the caspase-3 activity was measured in the cell culture medium. To get an impression of the caspase-3 release, a ratio between caspase-3 activity obtained in medium and total caspase-3 activity obtained in medium and cell lysate was calculated.

Supplementary figure 2 Total cellular protein amount, caspase 3 activity and caspase 3 release in HK-2 cells (A) and CCDSk (B) after 48 h incubation.

**Methods – Inhibition experiments**

CCDSk cells were cultured in 6-well plates. After reaching confluence, the cells were serum-starved for 24 h. Subsequently, the cells were incubated for an additional 48 h with control medium + DMSO, lactic acid + DMSO, or lactic acid and control media in combination with either an inhibitor of EGFR (1 µM AG1478), an inhibitor of ERK1/2 (10 µM U0126), or an inhibitor of Rho/ROCK signaling (1 µM Y-27632).

Afterwards, the cells were washed once with 1 mL of 1× PBS and lysed in 50 µL of RIPA buffer. The lysates were homogenized by 10 cycles of ultrasonication. The lysates were centrifuged at 14,000 × g for 10 min at 4°C, and the supernatants were transferred to new reaction tubes, mixed with 6× Laemmli buffer, and heated to 95°C for 5 min.

Proteins were separated by 14% sodium dodecyl sulfate–polyacrylamide gel electrophoresis (SDS-PAGE) and transferred onto nitrocellulose membranes. Total protein on the membranes was stained with Ponceau S solution (AppliChem GmbH, Darmstadt, Germany) and documented using the Bio-Rad ChemiDoc™ XRS imaging system (Bio-Rad Laboratories GmbH, Feldkirchen, Germany). When Ponceau S staining was technically inadequate or the lanes were too closely spaced to allow reliable normalization, β-actin was additionally used as a reference protein. Figure S5 shows that the lactic acid-induced IL-6 expression depends on ERK1/2 activity, but is independent of EGFR or Rho/ROCK signaling.

Supplementary figure 3 Western Blot analysis of phosphorylated ERK1/2 and total ERK1/2 in CCDSk after incubation with lactic acid.

Figure S3 shows that none of the experimental conditions caused a change of ERK1/2 phosphorylation or expression in CCDSk neither after 3 h, nor after 48 h.

Figure S4 shows that lactic acid induces an EGFR-phosphorylation after 48 h in CCDSk cells.

Figure S5-7 suggests that the ERK1/2 signaling was involved in lactic-induced IL-6, P-SRF and cFos expression. Furthermore it was shown that lactic acid induced P-SRF expression was partially mediated by the Rho/ROCK signaling and the lactic acid induced cFos expression depended partially on the EGFR-singaling.

Supplementary figure 4 Western Blot analysis of phosphorylated EGFR and total EGFR in CCDSk after incubation with lactic acid.

Supplementary figure 5 Western Blot analysis of IL-6 in CCDSk after incubation with lactic acid, inhibition of EGFR-activation (AG1478), inhibition of ERK1/2 activation (U0126) and inhibition of Rho/Rock pathway (Y-27632).

Supplementary figure 6 Western Blot analysis of phosphorylation SRF in CCDSk after incubation with lactic acid, inhibition of EGFR-activation (AG1478), inhibition of ERK1/2 activation (U0126) and inhibition of Rho/Rock pathway (Y-27632).

Supplementary figure 7 Western Blot analysis of c-Fos in CCDSk after incubation with lactic acid, inhibition of EGFR-activation (AG1478), inhibition of ERK1/2 activation (U0126) and inhibition of Rho/Rock pathway (Y-27632).

Supplementary Table 1 Composition of used buffers and solutions

| **Buffer** | **Composition** |
| --- | --- |
| Caspase-3 reaction buffer | - 10 mM PIPES - 2 mM EDTA - 0.1 % CHAPS - 1 % DTT (freshly added) - pH 7.5 |
| EDTA buffer | - 136.8 mM NaCl - 2.68 mM KCl - 8.1 mM Na_2_HPO_4_ - 2 mM H_2_PO_4_^-^ - 0,7 mM EDTA - pH 7,2 |
| MOPS Triton buffer | - 10 mM Tris base - 20 mM MOPS - 100 mM NaCl - 1 mM EDTA - 0.01% Triton X-100 - pH 7.5 |
| 1x PBS | - 1370 mM NaCl - 27 mM KCl - 81 mM Na_2_HPO_4_ * 2 H_2_O - 15 mM KH_2_PO_4_ - pH 7.4 |
| Permeabilization buffer | - 1 x PBS + 0.1 % Triton X-100 |
| Blocking solution for BrdU-ELISA | - Permeabilization buffer + 10 % FCS |
| RIPA buffer | - 150 mM NaCl - 10 mM Tris (Base), pH 7.4 - 1 % (w/w) Nonidet P-40 - 0.1 % SDS - 1 % (w/v) Na-Deoxycholate - 0.1 % Triton X-100 - protease inhibitor cocktail - 1 mM EDTA - 1 mM Na-Orthovanadate - 1 mM NaF |
| 6 x Redmix/Lämmli buffer | - 124.8 mM TRIS HCl - 6 % SDS - 1,42 mM ß-Mercaptoethanol - 4,7 mM Glycerol - 155.2 mM Bromphenol Blue - pH 6.8 |
| Running buffer for western blot | - 25 mM TRIS - 3,5 mM SDS - 192 mM glycine |
| 1 x TBS | - 3 mM TRIS base - 140 mM NaCl - 0.17 mM TRIS-HCl - pH 7.4 |
| 1×Turbo-Transferbuffer | - 20 % 5× Transferbuffer (Bio-Rad, Feldkirchen, Ger) - 20 % ethanol (100 %) - 60 % pure water |
| Trypsin solution | - 154 mM NaCl - 2.7 mM KCl - 8.2 mM Na_2_HPO_4_ - 1.5 mM KH_2_PO_4_ - 0,7 mM EDTA - 0,2 mM streptomycin - 0,18 mM penicillin - 0.02 mM trypsin - pH 7,1-7,3 |

Supplementary Table 2 Number of replicates for RNA-Sequencing

| **Number of replicates N** | **HK2** | | **CCDSK** | |
| --- | --- | --- | --- | --- |
| Condition | HCl | LA | HCl | LA |
| Control | 5 | 5 | 6 | 5 |
| Acidosis | 6 | 5 | 6 | 5 |

Supplementary Table 3 Filter parameter for differentially expressed genes

| Parameters | |
| --- | --- |
| biotype | protein coding & lncRNA |
| DE (overlap DSeq2 and EdgeR, FDR<0.05) | ±1 |
| Abundance (FPM) | >3 |
| edgeR \|logFC\|HK-2 mono | >0.5 |
| edgeR \|logFC\|CCDSK mono | >0.5 |

Supplementary figure 8 Overlay histogram illustrating the distribution of DEGs in CCDSk after incubation with HCl (red) and lactic acid (blue)

Supplementary Table 4 List of protein coding DEGs of separate analysed HCl-treated CCDSk.

|  | **mean FPM_Mono_pH74** | **mean FPM_Mono_pH64** | **DE = 1** | **log2FC** |
| --- | --- | --- | --- | --- |
| ABCA3 | 3 | 5 | 1 | 0,61 |
| ABCA5 | 30 | 46 | 1 | 0,59 |
| ABCA6 | 23 | 14 | 1 | -0,76 |
| ABCA8 | 10 | 4 | 1 | -1,42 |
| ABCA9 | 32 | 12 | 1 | -1,44 |
| ABCB4 | 2 | 3 | 1 | 0,62 |
| ABCB7 | 22 | 31 | 1 | 0,54 |
| ABHD11 | 11 | 7 | 1 | -0,55 |
| ABHD17C | 19 | 27 | 1 | 0,52 |
| ABI3BP | 67 | 39 | 1 | -0,77 |
| ABLIM3 | 52 | 36 | 1 | -0,52 |
| ACAN | 212 | 519 | 1 | 1,29 |
| ACSS1 | 16 | 24 | 1 | 0,6 |
| ACTA2 | 100 | 162 | 1 | 0,7 |
| ACTA2-AS1 | 2 | 3 | 1 | 1,09 |
| ACTB | 1913 | 3032 | 1 | 0,66 |
| ACTG1 | 2553 | 4007 | 1 | 0,65 |
| ACTG2 | 3 | 6 | 1 | 0,83 |
| ACTN1 | 259 | 376 | 1 | 0,54 |
| ACTN4 | 287 | 408 | 1 | 0,51 |
| ACYP1 | 8 | 5 | 1 | -0,61 |
| ADAMTS1 | 232 | 350 | 1 | 0,59 |
| ADAMTS15 | 48 | 25 | 1 | -0,94 |
| ADAMTS7 | 98 | 218 | 1 | 1,15 |
| ADAMTS8 | 9 | 3 | 1 | -1,7 |
| ADGRL4 | 14 | 9 | 1 | -0,69 |
| ADPRH | 3 | 6 | 1 | 0,83 |
| ADRA1D | 9 | 14 | 1 | 0,66 |
| ADRA2A | 4 | 7 | 1 | 0,81 |
| ADRA2C | 2 | 4 | 1 | 0,94 |
| AHRR | 3 | 5 | 1 | 0,76 |
| AK5 | 30 | 45 | 1 | 0,56 |
| AKAP6 | 13 | 23 | 1 | 0,86 |
| AKR1C1 | 30 | 66 | 1 | 1,15 |
| ALCAM | 117 | 168 | 1 | 0,52 |
| ALDH1B1 | 27 | 45 | 1 | 0,76 |
| ALDH3A2 | 48 | 77 | 1 | 0,69 |
| ALDH4A1 | 25 | 37 | 1 | 0,58 |
| ALDOC | 8 | 18 | 1 | 1,14 |
| ALS2CL | 4 | 8 | 1 | 0,96 |
| AMIGO2 | 24 | 35 | 1 | 0,57 |
| AMY2B | 5 | 3 | 1 | -0,55 |
| ANG | 4 | 6 | 1 | 0,61 |
| ANGPT1 | 20 | 29 | 1 | 0,54 |
| ANK2 | 31 | 17 | 1 | -0,85 |
| ANKRD13A | 100 | 162 | 1 | 0,7 |
| ANKRD34A | 3 | 4 | 1 | 0,54 |
| ANPEP | 398 | 279 | 1 | -0,51 |
| ANXA3 | 11 | 16 | 1 | 0,61 |
| APOD | 83 | 34 | 1 | -1,29 |
| ARHGAP22 | 45 | 32 | 1 | -0,51 |
| ARHGAP26 | 20 | 30 | 1 | 0,6 |
| ARHGAP39 | 7 | 4 | 1 | -0,74 |
| ARHGAP6 | 6 | 9 | 1 | 0,62 |
| ARHGEF2 | 183 | 122 | 1 | -0,59 |
| ARHGEF37 | 2 | 4 | 1 | 1 |
| ARSK | 11 | 16 | 1 | 0,5 |
| B3GALT2 | 4 | 5 | 1 | 0,53 |
| BAMBI | 22 | 33 | 1 | 0,6 |
| BBS5 | 11 | 7 | 1 | -0,63 |
| BDKRB1 | 24 | 11 | 1 | -1,14 |
| BDKRB2 | 106 | 62 | 1 | -0,79 |
| C10orf143 | 4 | 3 | 1 | -0,67 |
| C1orf115 | 3 | 5 | 1 | 0,77 |
| C1orf198 | 194 | 279 | 1 | 0,52 |
| CAND2 | 4 | 6 | 1 | 0,71 |
| CARMN | 21 | 38 | 1 | 0,84 |
| CCDC136 | 11 | 16 | 1 | 0,56 |
| CCDC14 | 83 | 134 | 1 | 0,68 |
| CCDC80 | 992 | 1409 | 1 | 0,51 |
| CD34 | 3 | 1 | 1 | -1,2 |
| CDA | 3 | 4 | 1 | 0,63 |
| CDKN2B | 23 | 13 | 1 | -0,8 |
| CEMIP | 791 | 1667 | 1 | 1,08 |
| CENPJ | 4 | 6 | 1 | 0,53 |
| CEROX1 | 7 | 3 | 1 | -1,12 |
| CFL2 | 90 | 154 | 1 | 0,77 |
| CHRM2 | 27 | 42 | 1 | 0,62 |
| CHST2 | 30 | 19 | 1 | -0,7 |
| CLDN11 | 318 | 163 | 1 | -0,96 |
| CLEC2B | 7 | 5 | 1 | -0,7 |
| CLN6 | 7 | 5 | 1 | -0,58 |
| CMKLR2 | 23 | 14 | 1 | -0,67 |
| CNN1 | 58 | 113 | 1 | 0,97 |
| CNTN3 | 27 | 48 | 1 | 0,83 |
| COBLL1 | 29 | 46 | 1 | 0,65 |
| COL11A1 | 17 | 25 | 1 | 0,55 |
| COL12A1 | 881 | 1290 | 1 | 0,55 |
| COL14A1 | 42 | 19 | 1 | -1,1 |
| COL26A1 | 12 | 4 | 1 | -1,62 |
| CORO2B | 11 | 7 | 1 | -0,76 |
| CRIM1 | 335 | 483 | 1 | 0,53 |
| CSPG4P11 | 3 | 5 | 1 | 0,66 |
| CSRP2 | 18 | 36 | 1 | 0,97 |
| CYP3A5 | 4 | 3 | 1 | -0,52 |
| CYP4V2 | 22 | 15 | 1 | -0,55 |
| DAAM2 | 52 | 77 | 1 | 0,59 |
| DAPK1 | 62 | 89 | 1 | 0,52 |
| DDIT3 | 36 | 23 | 1 | -0,65 |
| DDIT4 | 81 | 40 | 1 | -1,03 |
| DENND2D | 5 | 8 | 1 | 0,63 |
| DENND3 | 22 | 33 | 1 | 0,58 |
| DHCR7 | 22 | 46 | 1 | 1,08 |
| DHFR2 | 8 | 5 | 1 | -0,51 |
| DIPK1A | 34 | 48 | 1 | 0,51 |
| DIRAS1 | 21 | 31 | 1 | 0,59 |
| DLGAP1-AS2 | 6 | 3 | 1 | -0,88 |
| DNM3OS | 41 | 29 | 1 | -0,51 |
| DPYSL4 | 3 | 2 | 1 | -0,55 |
| DUSP6 | 22 | 9 | 1 | -1,2 |
| EBF1 | 18 | 12 | 1 | -0,67 |
| EDN1 | 9 | 14 | 1 | 0,62 |
| EEF1A1P11 | 61 | 89 | 1 | 0,55 |
| EEF1A1P6 | 188 | 280 | 1 | 0,57 |
| EFNB1 | 33 | 23 | 1 | -0,55 |
| EGR2 | 14 | 5 | 1 | -1,6 |
| ELFN2 | 8 | 4 | 1 | -0,9 |
| EPHB6 | 94 | 164 | 1 | 0,79 |
| ERMP1 | 30 | 43 | 1 | 0,52 |
| ESM1 | 5 | 2 | 1 | -1,37 |
| ETV1 | 12 | 5 | 1 | -1,28 |
| ETV4 | 20 | 12 | 1 | -0,67 |
| ETV5 | 30 | 19 | 1 | -0,68 |
| EXPH5 | 4 | 7 | 1 | 0,57 |
| FADS1 | 50 | 77 | 1 | 0,64 |
| FADS2 | 105 | 174 | 1 | 0,73 |
| FAIM2 | 4 | 2 | 1 | -1,21 |
| FAM167A | 24 | 13 | 1 | -0,86 |
| FAM20A | 43 | 30 | 1 | -0,51 |
| FAM86EP | 4 | 3 | 1 | -0,55 |
| FDFT1 | 68 | 100 | 1 | 0,55 |
| FGF1 | 19 | 40 | 1 | 1,09 |
| FHL1 | 499 | 727 | 1 | 0,54 |
| FHOD1 | 37 | 26 | 1 | -0,52 |
| FHOD3 | 15 | 23 | 1 | 0,64 |
| FIBIN | 17 | 11 | 1 | -0,6 |
| FJX1 | 27 | 18 | 1 | -0,62 |
| FNDC10 | 17 | 11 | 1 | -0,69 |
| FZD8 | 9 | 4 | 1 | -1,04 |
| GABBR2 | 10 | 18 | 1 | 0,9 |
| GALNT5 | 85 | 132 | 1 | 0,63 |
| GAS7 | 40 | 21 | 1 | -0,94 |
| GBP1 | 19 | 28 | 1 | 0,61 |
| GCNT4 | 18 | 29 | 1 | 0,7 |
| GEM | 23 | 32 | 1 | 0,5 |
| GIPC3 | 2 | 4 | 1 | 1 |
| GNG11 | 87 | 61 | 1 | -0,52 |
| GNG2 | 17 | 12 | 1 | -0,54 |
| GPC1 | 259 | 365 | 1 | 0,5 |
| GPCPD1 | 20 | 13 | 1 | -0,7 |
| GPR155 | 5 | 6 | 1 | 0,54 |
| GPR176 | 129 | 185 | 1 | 0,53 |
| GPR68 | 18 | 9 | 1 | -1,04 |
| GREM1 | 499 | 1166 | 1 | 1,22 |
| GREM2 | 39 | 20 | 1 | -0,95 |
| GRIA1 | 121 | 177 | 1 | 0,56 |
| GRIK2 | 38 | 63 | 1 | 0,73 |
| GRK3 | 4 | 6 | 1 | 0,74 |
| GSTM3 | 70 | 103 | 1 | 0,55 |
| GTF2IP20 | 8 | 5 | 1 | -0,64 |
| GYG1 | 76 | 114 | 1 | 0,57 |
| H1-10-AS1 | 4 | 3 | 1 | -0,55 |
| H19 | 61 | 14 | 1 | -2,17 |
| HACD1 | 18 | 28 | 1 | 0,61 |
| HAPLN3 | 75 | 131 | 1 | 0,81 |
| HAUS8 | 3 | 2 | 1 | -0,61 |
| HBA1 | 7 | 4 | 1 | -0,9 |
| HBA2 | 8 | 4 | 1 | -1,01 |
| HDAC9 | 37 | 57 | 1 | 0,63 |
| HES1 | 5 | 2 | 1 | -1,22 |
| HGF | 9 | 4 | 1 | -1,34 |
| HIC1 | 90 | 56 | 1 | -0,69 |
| HMGA2 | 62 | 39 | 1 | -0,68 |
| HRCT1 | 2 | 4 | 1 | 0,59 |
| HS3ST3A1 | 23 | 16 | 1 | -0,51 |
| HSD17B7 | 5 | 7 | 1 | 0,55 |
| HSPB3 | 8 | 20 | 1 | 1,35 |
| HSPB7 | 26 | 73 | 1 | 1,51 |
| HUNK | 8 | 13 | 1 | 0,73 |
| HYAL3 | 4 | 6 | 1 | 0,51 |
| HYKK | 2 | 3 | 1 | 0,55 |
| ICOSLG | 7 | 2 | 1 | -1,61 |
| IDH2 | 55 | 89 | 1 | 0,7 |
| IDI1 | 37 | 56 | 1 | 0,6 |
| IKBKE | 12 | 8 | 1 | -0,53 |
| IL16 | 4 | 2 | 1 | -0,75 |
| IL6R | 4 | 7 | 1 | 0,83 |
| INSIG1 | 41 | 81 | 1 | 0,99 |
| INSYN2B | 7 | 4 | 1 | -0,79 |
| IRAG1 | 42 | 96 | 1 | 1,18 |
| ISLR | 199 | 120 | 1 | -0,73 |
| ITGA7 | 13 | 20 | 1 | 0,59 |
| ITGA8 | 9 | 29 | 1 | 1,6 |
| ITGB3 | 17 | 10 | 1 | -0,79 |
| JPH2 | 19 | 34 | 1 | 0,88 |
| KANK1 | 56 | 90 | 1 | 0,69 |
| KCTD16 | 4 | 6 | 1 | 0,69 |
| KIAA0895L | 16 | 10 | 1 | -0,55 |
| KIAA1755 | 23 | 16 | 1 | -0,53 |
| KLF10 | 22 | 14 | 1 | -0,68 |
| KLF5 | 9 | 15 | 1 | 0,67 |
| KLHL3 | 2 | 3 | 1 | 0,77 |
| L3MBTL2-AS1 | 5 | 3 | 1 | -0,9 |
| LAMA3 | 20 | 43 | 1 | 1,09 |
| LBH | 217 | 418 | 1 | 0,95 |
| LDLR | 49 | 78 | 1 | 0,67 |
| LENG1 | 4 | 2 | 1 | -0,97 |
| LEPR | 18 | 33 | 1 | 0,86 |
| LFNG | 15 | 9 | 1 | -0,77 |
| LHFPL2 | 33 | 23 | 1 | -0,54 |
| LIF | 3 | 2 | 1 | -0,62 |
| LIMD2 | 15 | 10 | 1 | -0,52 |
| LIN7A | 17 | 26 | 1 | 0,54 |
| LINC00578 | 2 | 3 | 1 | 0,54 |
| LINC01963 | 8 | 5 | 1 | -0,74 |
| LINC02202 | 3 | 1 | 1 | -1,15 |
| LMLN | 5 | 3 | 1 | -0,51 |
| LMOD1 | 72 | 149 | 1 | 1,04 |
| LPAR6 | 13 | 9 | 1 | -0,52 |
| LRP11 | 3 | 4 | 1 | 0,57 |
| LRP8 | 5 | 8 | 1 | 0,69 |
| LRRFIP2 | 105 | 152 | 1 | 0,54 |
| MACROD2 | 2 | 4 | 1 | 0,79 |
| MAGI1 | 17 | 24 | 1 | 0,5 |
| MAMLD1 | 42 | 59 | 1 | 0,5 |
| MAP2K6 | 8 | 12 | 1 | 0,65 |
| MAP3K4-AS1 | 4 | 3 | 1 | -0,71 |
| MAP3K8 | 7 | 12 | 1 | 0,8 |
| MAPK8IP1 | 12 | 8 | 1 | -0,56 |
| MASP1 | 174 | 404 | 1 | 1,22 |
| MBOAT1 | 8 | 14 | 1 | 0,76 |
| MCTP2 | 3 | 5 | 1 | 0,55 |
| ME1 | 16 | 22 | 1 | 0,5 |
| MEDAG | 67 | 34 | 1 | -0,98 |
| MEF2A | 35 | 56 | 1 | 0,67 |
| MEG9 | 8 | 5 | 1 | -0,56 |
| MICA | 26 | 39 | 1 | 0,57 |
| MIOX | 4 | 2 | 1 | -1,02 |
| MIR222HG | 37 | 26 | 1 | -0,5 |
| MIR503HG | 35 | 18 | 1 | -0,95 |
| MIR99AHG | 3 | 2 | 1 | -0,55 |
| MLLT3 | 22 | 33 | 1 | 0,57 |
| MSMO1 | 51 | 90 | 1 | 0,81 |
| MT1E | 43 | 31 | 1 | -0,5 |
| MT2A | 167 | 86 | 1 | -0,96 |
| MTND4P12 | 2 | 3 | 1 | 0,6 |
| MTSS2 | 63 | 112 | 1 | 0,84 |
| MVK | 14 | 21 | 1 | 0,59 |
| MYH10 | 161 | 242 | 1 | 0,59 |
| MYH2 | 1 | 4 | 1 | 1,78 |
| MYLK | 248 | 357 | 1 | 0,52 |
| MYOCD | 3 | 7 | 1 | 1,12 |
| MYOM1 | 6 | 9 | 1 | 0,63 |
| N4BP2L1 | 4 | 7 | 1 | 0,61 |
| NAAA | 44 | 62 | 1 | 0,5 |
| NAP1L3 | 4 | 2 | 1 | -1,01 |
| NDUFA4L2 | 10 | 5 | 1 | -0,94 |
| NEK8 | 5 | 4 | 1 | -0,51 |
| NEXN | 48 | 69 | 1 | 0,54 |
| NFYB | 51 | 76 | 1 | 0,58 |
| NTSR1 | 8 | 2 | 1 | -1,76 |
| NXPH4 | 12 | 7 | 1 | -0,69 |
| OAF | 207 | 126 | 1 | -0,71 |
| OLFML1 | 33 | 20 | 1 | -0,69 |
| OMD | 16 | 6 | 1 | -1,34 |
| OSR1 | 19 | 13 | 1 | -0,54 |
| OXTR | 58 | 101 | 1 | 0,79 |
| PALLD | 201 | 317 | 1 | 0,66 |
| PCOLCE2 | 66 | 39 | 1 | -0,77 |
| PDE4DIPP2 | 48 | 77 | 1 | 0,67 |
| PENK | 5 | 8 | 1 | 0,56 |
| PERP | 164 | 232 | 1 | 0,5 |
| PHGDH | 385 | 273 | 1 | -0,5 |
| PHLDA1 | 48 | 32 | 1 | -0,62 |
| PITPNM3 | 9 | 12 | 1 | 0,53 |
| PLAC9 | 88 | 60 | 1 | -0,57 |
| PLAU | 74 | 38 | 1 | -0,96 |
| PLEKHG4 | 59 | 34 | 1 | -0,81 |
| PLEKHG5 | 20 | 30 | 1 | 0,59 |
| PLPPR4 | 10 | 14 | 1 | 0,55 |
| PLXNC1 | 33 | 56 | 1 | 0,76 |
| PNMA2 | 5 | 3 | 1 | -0,65 |
| PNPLA3 | 12 | 21 | 1 | 0,85 |
| POSTN | 557 | 323 | 1 | -0,79 |
| POU2F2 | 19 | 11 | 1 | -0,78 |
| PPFIBP2 | 10 | 15 | 1 | 0,56 |
| PRELID2 | 5 | 7 | 1 | 0,6 |
| PRMT6 | 15 | 10 | 1 | -0,59 |
| PROCR | 10 | 7 | 1 | -0,52 |
| PRR5L | 6 | 11 | 1 | 0,87 |
| PRSS12 | 153 | 217 | 1 | 0,51 |
| PRSS3 | 6 | 4 | 1 | -0,71 |
| PRUNE2 | 28 | 51 | 1 | 0,89 |
| PSG1 | 3 | 8 | 1 | 1,58 |
| PSG5 | 219 | 538 | 1 | 1,3 |
| PSMG3-AS1 | 7 | 5 | 1 | -0,56 |
| PTGES | 33 | 22 | 1 | -0,59 |
| PTGS2 | 9 | 5 | 1 | -0,79 |
| PYGL | 29 | 43 | 1 | 0,56 |
| QPCTL | 9 | 13 | 1 | 0,52 |
| RAB11FIP2 | 66 | 96 | 1 | 0,54 |
| RASA2 | 46 | 65 | 1 | 0,51 |
| RASD2 | 1 | 3 | 1 | 1,22 |
| RASGEF1A | 3 | 5 | 1 | 0,84 |
| RASGRP3 | 2 | 3 | 1 | 0,52 |
| RASL12 | 10 | 6 | 1 | -0,84 |
| RASSF3 | 23 | 34 | 1 | 0,58 |
| RBM24 | 7 | 11 | 1 | 0,59 |
| RCAN2 | 66 | 95 | 1 | 0,52 |
| REV3L | 82 | 134 | 1 | 0,7 |
| RFLNB | 52 | 77 | 1 | 0,55 |
| RGS9 | 4 | 6 | 1 | 0,76 |
| RNF152 | 8 | 4 | 1 | -0,86 |
| ROCK2 | 102 | 157 | 1 | 0,61 |
| ROR1 | 23 | 14 | 1 | -0,71 |
| RPSAP12 | 4 | 7 | 1 | 0,64 |
| S100A3 | 14 | 9 | 1 | -0,55 |
| SAMD12 | 4 | 7 | 1 | 0,58 |
| SCARA5 | 16 | 4 | 1 | -2,04 |
| SCN3A | 2 | 6 | 1 | 1,59 |
| SEPTIN5 | 2 | 3 | 1 | 0,57 |
| SERAC1 | 14 | 21 | 1 | 0,58 |
| SERPINB2 | 8 | 17 | 1 | 1,11 |
| SERPINB7 | 4 | 20 | 1 | 2,38 |
| SESN2 | 52 | 33 | 1 | -0,66 |
| SH2D5 | 7 | 9 | 1 | 0,52 |
| SLC12A8 | 3 | 5 | 1 | 0,61 |
| SLC18B1 | 4 | 3 | 1 | -0,75 |
| SLC1A2 | 5 | 4 | 1 | -0,63 |
| SLC20A1 | 124 | 62 | 1 | -1 |
| SLC29A1 | 14 | 21 | 1 | 0,65 |
| SLC2A1 | 73 | 108 | 1 | 0,56 |
| SLC38A11 | 4 | 9 | 1 | 1,21 |
| SLC38A4 | 7 | 12 | 1 | 0,69 |
| SLC7A14 | 4 | 2 | 1 | -1,18 |
| SLC8A1 | 29 | 54 | 1 | 0,91 |
| SLX4IP | 4 | 2 | 1 | -0,56 |
| SMTN | 69 | 98 | 1 | 0,5 |
| SNAI2 | 65 | 44 | 1 | -0,56 |
| SOCS2 | 8 | 11 | 1 | 0,55 |
| SORT1 | 31 | 66 | 1 | 1,08 |
| SOX8 | 7 | 3 | 1 | -1,19 |
| SOX9 | 8 | 4 | 1 | -1,04 |
| SPON2 | 81 | 52 | 1 | -0,64 |
| SPRY1 | 13 | 8 | 1 | -0,76 |
| SPRY4 | 18 | 12 | 1 | -0,64 |
| SRSF6 | 116 | 76 | 1 | -0,6 |
| STARD4 | 23 | 34 | 1 | 0,55 |
| STEAP2 | 15 | 22 | 1 | 0,58 |
| STMN3 | 22 | 13 | 1 | -0,77 |
| STRA6 | 13 | 6 | 1 | -1,27 |
| STXBP4 | 8 | 6 | 1 | -0,52 |
| STXBP6 | 5 | 7 | 1 | 0,54 |
| SYNC | 20 | 29 | 1 | 0,55 |
| SYNE3 | 95 | 67 | 1 | -0,5 |
| SYNPO2 | 44 | 98 | 1 | 1,16 |
| SYT7 | 4 | 2 | 1 | -0,95 |
| SYTL2 | 21 | 35 | 1 | 0,72 |
| TAFA5 | 30 | 44 | 1 | 0,55 |
| TAGLN | 270 | 465 | 1 | 0,78 |
| TANC2 | 40 | 57 | 1 | 0,51 |
| TENM2 | 2 | 5 | 1 | 0,98 |
| TENM4 | 16 | 6 | 1 | -1,4 |
| THBS2 | 461 | 695 | 1 | 0,59 |
| TIPARP | 34 | 47 | 1 | 0,5 |
| TM4SF20 | 4 | 11 | 1 | 1,3 |
| TMEM106A | 4 | 5 | 1 | 0,51 |
| TMEM145 | 7 | 5 | 1 | -0,6 |
| TMEM47 | 173 | 258 | 1 | 0,58 |
| TNXA | 112 | 67 | 1 | -0,74 |
| TNXB | 674 | 453 | 1 | -0,57 |
| TPD52L1 | 19 | 30 | 1 | 0,62 |
| TPPP3 | 5 | 2 | 1 | -1,35 |
| TRAF3IP2 | 26 | 38 | 1 | 0,52 |
| TRAF5 | 31 | 43 | 1 | 0,51 |
| TSKU | 33 | 24 | 1 | -0,5 |
| TSPAN13 | 12 | 17 | 1 | 0,55 |
| TSPAN18 | 6 | 10 | 1 | 0,79 |
| TUBA4A | 4 | 7 | 1 | 0,81 |
| TXNDC16 | 6 | 8 | 1 | 0,59 |
| TXNRD1 | 318 | 531 | 1 | 0,74 |
| TYMP | 17 | 12 | 1 | -0,52 |
| UNC13B | 21 | 30 | 1 | 0,52 |
| UNC5B | 132 | 84 | 1 | -0,65 |
| UPP1 | 41 | 26 | 1 | -0,64 |
| USP53 | 234 | 350 | 1 | 0,58 |
| VAT1L | 4 | 6 | 1 | 0,72 |
| VGLL3 | 293 | 435 | 1 | 0,57 |
| WFDC1 | 15 | 36 | 1 | 1,31 |
| ZMYND8 | 21 | 15 | 1 | -0,54 |

Supplementary Table 5 Enriched GO terms in CCDSk after incubation with HCl

| **source** | **term_name** | **term_id** | **adjusted_p_value** | **enrichment** |
| --- | --- | --- | --- | --- |
| GO:MF | cytoskeletal protein binding | GO:0008092 | 3.6E-05 | 2.5 |
| GO:MF | actin binding | GO:0003779 | 1.3E-04 | 3.3 |
| GO:MF | sulfur compound binding | GO:1901681 | 1.7E-03 | 3.8 |
| GO:MF | glycosaminoglycan binding | GO:0005539 | 2.6E-03 | 3.8 |
| GO:MF | protein binding | GO:0005515 | 3.6E-03 | 1.1 |
| GO:MF | heparin binding | GO:0008201 | 4.3E-03 | 4.4 |
| GO:MF | ABC-type transporter activity | GO:0140359 | 9.8E-03 | 8.3 |
| GO:MF | filamin binding | GO:0031005 | 2.8E-02 | 18.0 |
| GO:MF | actin filament binding | GO:0051015 | 3.2E-02 | 3.7 |
| GO:MF | lipid transporter activity | GO:0005319 | 3.3E-02 | 3.9 |
| GO:MF | alpha-adrenergic receptor activity | GO:0004936 | 5.0E-02 | 29.2 |
| GO:BP | anatomical structure development | GO:0048856 | 1.1E-12 | 1.7 |
| GO:BP | glomerulus vasculature development | GO:0072012 | 1.1E-06 | 19.2 |
| GO:BP | multicellular organismal process | GO:0032501 | 6.3E-12 | 1.6 |
| GO:BP | animal organ development | GO:0048513 | 7.3E-12 | 2.2 |
| GO:BP | multicellular organism development | GO:0007275 | 1.7E-11 | 1.8 |
| GO:BP | system development | GO:0048731 | 2.8E-11 | 1.9 |
| GO:BP | anatomical structure morphogenesis | GO:0009653 | 4.8E-11 | 2.2 |
| GO:BP | tissue development | GO:0009888 | 1.3E-10 | 2.4 |
| GO:BP | anatomical structure formation involved in morphogenesis | GO:0048646 | 3.0E-10 | 3.0 |
| GO:BP | developmental process | GO:0032502 | 3.8E-10 | 1.6 |
| GO:BP | renal system vasculature development | GO:0061437 | 2.2E-06 | 18.0 |
| GO:BP | regulation of multicellular organismal process | GO:0051239 | 1.5E-08 | 2.0 |
| GO:BP | response to stimulus | GO:0050896 | 1.9E-08 | 1.4 |
| GO:BP | positive regulation of biological process | GO:0048518 | 2.0E-07 | 1.6 |
| GO:BP | kidney vasculature development | GO:0061440 | 2.2E-06 | 18.0 |
| GO:CC | extracellular matrix | GO:0031012 | 9.0E-08 | 4.6 |
| GO:BP | positive regulation of multicellular organismal process | GO:0051240 | 5.2E-07 | 2.3 |
| GO:BP | cell differentiation | GO:0030154 | 9.3E-07 | 1.7 |
| GO:BP | cellular developmental process | GO:0048869 | 9.4E-07 | 1.7 |
| GO:CC | external encapsulating structure | GO:0030312 | 9.6E-08 | 4.6 |
| GO:BP | cell adhesion | GO:0007155 | 1.3E-06 | 2.4 |
| GO:BP | renal system development | GO:0072001 | 2.3E-06 | 4.5 |
| GO:BP | kidney development | GO:0001822 | 6.0E-06 | 4.4 |
| GO:BP | muscle tissue development | GO:0060537 | 3.0E-06 | 4.0 |
| GO:BP | cellular response to stimulus | GO:0051716 | 2.3E-06 | 1.4 |
| GO:BP | muscle structure development | GO:0061061 | 2.8E-09 | 3.7 |
| GO:CC | actin cytoskeleton | GO:0015629 | 4.5E-06 | 3.4 |
| GO:BP | tube morphogenesis | GO:0035239 | 7.8E-06 | 2.8 |
| GO:BP | regulation of response to stimulus | GO:0048583 | 1.0E-05 | 1.7 |
| GO:BP | nephron development | GO:0072006 | 1.5E-05 | 6.2 |
| GO:BP | regulation of cell communication | GO:0010646 | 1.8E-05 | 1.7 |
| GO:BP | positive regulation of cellular process | GO:0048522 | 1.9E-05 | 1.5 |
| GO:BP | response to endogenous stimulus | GO:0009719 | 2.4E-05 | 2.3 |
| GO:BP | blood vessel morphogenesis | GO:0048514 | 2.9E-05 | 3.1 |
| GO:BP | signaling | GO:0023052 | 3.0E-05 | 1.5 |
| GO:BP | signal transduction | GO:0007165 | 3.4E-05 | 1.5 |
| GO:BP | regulation of signaling | GO:0023051 | 3.4E-05 | 1.7 |
| GO:BP | alcohol metabolic process | GO:0006066 | 3.6E-05 | 4.0 |
| GO:BP | biological regulation | GO:0065007 | 4.2E-05 | 1.2 |
| GO:BP | muscle cell differentiation | GO:0042692 | 5.1E-05 | 3.8 |
| GO:BP | tube development | GO:0035295 | 5.2E-05 | 2.5 |
| GO:BP | secondary alcohol metabolic process | GO:1902652 | 6.9E-05 | 6.0 |
| GO:BP | sterol metabolic process | GO:0016125 | 7.6E-05 | 6.0 |
| GO:BP | cell communication | GO:0007154 | 8.7E-05 | 1.4 |
| GO:BP | regulation of developmental process | GO:0050793 | 1.0E-04 | 1.9 |
| GO:BP | cell-substrate adhesion | GO:0031589 | 1.4E-04 | 3.9 |
| GO:BP | regulation of signal transduction | GO:0009966 | 1.5E-04 | 1.8 |
| GO:BP | regulation of biological process | GO:0050789 | 1.6E-04 | 1.2 |
| GO:BP | cholesterol metabolic process | GO:0008203 | 1.9E-04 | 6.1 |
| GO:BP | muscle cell development | GO:0055001 | 1.9E-04 | 5.2 |
| GO:BP | smooth muscle contraction | GO:0006939 | 2.1E-04 | 7.3 |
| GO:BP | lipid localization | GO:0010876 | 2.2E-04 | 3.4 |
| GO:BP | cell migration | GO:0016477 | 2.5E-04 | 2.1 |
| GO:BP | heart development | GO:0007507 | 2.8E-04 | 3.0 |
| GO:BP | lipid transport | GO:0006869 | 3.9E-04 | 3.4 |
| GO:BP | cellular response to chemical stimulus | GO:0070887 | 5.2E-04 | 1.9 |
| GO:BP | positive regulation of locomotion | GO:0040017 | 7.9E-04 | 3.0 |
| GO:BP | regulation of cell-substrate adhesion | GO:0010810 | 8.5E-04 | 4.7 |
| GO:BP | response to growth factor | GO:0070848 | 8.7E-04 | 2.7 |
| GO:BP | regulation of localization | GO:0032879 | 9.8E-04 | 1.9 |
| GO:BP | muscle organ development | GO:0007517 | 1.1E-03 | 3.6 |
| GO:BP | regulation of cell migration | GO:0030334 | 1.4E-03 | 2.4 |
| GO:BP | regulation of cell differentiation | GO:0045595 | 2.0E-03 | 2.0 |
| GO:BP | positive regulation of cell migration | GO:0030335 | 2.1E-03 | 3.0 |
| GO:BP | angiogenesis | GO:0001525 | 2.2E-03 | 2.9 |
| GO:BP | regulation of locomotion | GO:0040012 | 2.4E-03 | 2.3 |
| GO:BP | striated muscle cell differentiation | GO:0051146 | 2.4E-03 | 3.9 |
| GO:BP | cell development | GO:0048468 | 2.9E-03 | 1.7 |
| GO:BP | connective tissue development | GO:0061448 | 3.0E-03 | 3.8 |
| GO:BP | actin filament-based process | GO:0030029 | 3.0E-03 | 2.5 |
| GO:BP | steroid metabolic process | GO:0008202 | 3.2E-03 | 3.6 |
| GO:BP | homeostatic process | GO:0042592 | 4.1E-03 | 2.0 |
| GO:BP | cellular response to endogenous stimulus | GO:0071495 | 4.1E-03 | 2.1 |
| GO:BP | negative regulation of substrate adhesion-dependent cell spreading | GO:1900025 | 4.1E-03 | 22.1 |
| GO:BP | intracellular signaling cassette | GO:0141124 | 4.5E-03 | 1.9 |
| GO:BP | positive regulation of cell motility | GO:2000147 | 5.1E-03 | 2.8 |
| GO:BP | glomerulus development | GO:0032835 | 5.1E-03 | 7.7 |
| GO:BP | response to chemical | GO:0042221 | 5.6E-03 | 1.6 |
| GO:BP | regulation of cell motility | GO:2000145 | 6.1E-03 | 2.3 |
| GO:BP | regulation of transport | GO:0051049 | 6.3E-03 | 2.0 |
| GO:BP | smooth muscle tissue development | GO:0048745 | 6.5E-03 | 14.3 |
| GO:BP | response to stress | GO:0006950 | 6.8E-03 | 1.5 |
| GO:BP | metanephric nephron development | GO:0072210 | 6.9E-03 | 10.8 |
| GO:BP | regulation of intracellular signal transduction | GO:1902531 | 7.7E-03 | 1.8 |
| GO:BP | transport | GO:0006810 | 7.9E-03 | 1.5 |
| GO:BP | regulation of calcium ion transport | GO:0051924 | 8.2E-03 | 4.2 |
| GO:BP | negative regulation of signaling | GO:0023057 | 8.5E-03 | 2.0 |
| GO:BP | regulation of calcium ion transmembrane transport | GO:1903169 | 8.8E-03 | 5.2 |
| GO:BP | glomerular mesangium development | GO:0072109 | 8.8E-03 | 19.3 |
| GO:BP | regulation of cell adhesion | GO:0030155 | 9.1E-03 | 2.5 |
| GO:BP | kidney morphogenesis | GO:0060993 | 9.2E-03 | 6.3 |
| GO:BP | locomotion | GO:0040011 | 9.5E-03 | 2.1 |
| GO:BP | cell surface receptor signaling pathway | GO:0007166 | 9.9E-03 | 1.7 |
| GO:BP | lipid metabolic process | GO:0006629 | 9.9E-03 | 2.0 |
| GO:BP | cellular response to growth factor stimulus | GO:0071363 | 1.0E-02 | 2.5 |
| GO:BP | mesenchyme development | GO:0060485 | 1.1E-02 | 3.5 |
| GO:BP | cardiocyte differentiation | GO:0035051 | 1.1E-02 | 5.1 |
| GO:BP | nephron epithelium development | GO:0072009 | 1.1E-02 | 5.5 |
| GO:BP | nephron epithelium morphogenesis | GO:0072088 | 1.1E-02 | 7.0 |
| GO:BP | negative regulation of cell-substrate adhesion | GO:0010812 | 1.1E-02 | 8.2 |
| GO:BP | regulation of cellular process | GO:0050794 | 1.2E-02 | 1.2 |
| GO:BP | actomyosin structure organization | GO:0031032 | 1.2E-02 | 4.3 |
| GO:BP | renal vesicle development | GO:0072087 | 1.2E-02 | 18.2 |
| GO:BP | cholesterol biosynthetic process | GO:0006695 | 1.3E-02 | 8.1 |
| GO:BP | secondary alcohol biosynthetic process | GO:1902653 | 1.3E-02 | 8.1 |
| GO:BP | actin cytoskeleton organization | GO:0030036 | 1.3E-02 | 2.5 |
| GO:BP | cell motility | GO:0048870 | 1.4E-02 | 1.9 |
| GO:BP | nephron morphogenesis | GO:0072028 | 1.4E-02 | 6.9 |
| GO:BP | regulation of animal organ morphogenesis | GO:2000027 | 1.5E-02 | 6.8 |
| GO:BP | kidney epithelium development | GO:0072073 | 1.5E-02 | 4.9 |
| GO:BP | positive regulation of developmental process | GO:0051094 | 1.6E-02 | 2.0 |
| GO:BP | muscle system process | GO:0003012 | 1.6E-02 | 3.0 |
| GO:BP | organic hydroxy compound transport | GO:0015850 | 1.6E-02 | 3.7 |
| GO:BP | tissue morphogenesis | GO:0048729 | 1.8E-02 | 2.6 |
| GO:BP | cellular component assembly involved in morphogenesis | GO:0010927 | 1.8E-02 | 5.3 |
| GO:BP | cellular anatomical entity morphogenesis | GO:0032989 | 1.8E-02 | 5.3 |
| GO:BP | negative regulation of cell communication | GO:0010648 | 1.9E-02 | 2.0 |
| GO:BP | striated muscle tissue development | GO:0014706 | 1.9E-02 | 3.1 |
| GO:BP | establishment of localization | GO:0051234 | 2.4E-02 | 1.4 |
| GO:BP | negative regulation of response to stimulus | GO:0048585 | 2.5E-02 | 1.9 |
| GO:BP | extracellular matrix organization | GO:0030198 | 2.5E-02 | 3.4 |
| GO:BP | muscle contraction | GO:0006936 | 2.6E-02 | 3.3 |
| GO:BP | extracellular structure organization | GO:0043062 | 2.6E-02 | 3.4 |
| GO:BP | external encapsulating structure organization | GO:0045229 | 2.8E-02 | 3.4 |
| GO:BP | striated muscle cell development | GO:0055002 | 2.9E-02 | 4.6 |
| GO:BP | muscle cell proliferation | GO:0033002 | 3.0E-02 | 4.2 |
| GO:BP | circulatory system process | GO:0003013 | 3.2E-02 | 2.6 |
| GO:BP | sterol biosynthetic process | GO:0016126 | 3.3E-02 | 7.2 |
| GO:BP | animal organ morphogenesis | GO:0009887 | 3.4E-02 | 2.2 |
| GO:BP | mesenchymal cell differentiation | GO:0048762 | 3.6E-02 | 3.7 |
| GO:BP | myofibril assembly | GO:0030239 | 3.7E-02 | 7.1 |
| GO:BP | metanephros development | GO:0001656 | 3.9E-02 | 6.1 |
| GO:BP | positive regulation of animal organ morphogenesis | GO:0110110 | 4.1E-02 | 10.6 |
| GO:BP | cell-substrate junction assembly | GO:0007044 | 4.3E-02 | 6.0 |
| GO:CC | cell junction | GO:0030054 | 9.5E-12 | 2.3 |
| GO:CC | cell periphery | GO:0071944 | 7.0E-09 | 1.6 |
| GO:CC | synapse | GO:0045202 | 8.3E-08 | 2.4 |
| GO:BP | blood vessel development | GO:0001568 | 4.6E-07 | 3.2 |
| GO:BP | circulatory system development | GO:0072359 | 2.8E-12 | 3.2 |
| GO:CC | cytoplasm | GO:0005737 | 1.5E-06 | 1.3 |
| GO:CC | anchoring junction | GO:0070161 | 2.8E-06 | 2.7 |
| GO:BP | vasculature development | GO:0001944 | 4.1E-07 | 3.2 |
| GO:CC | cell-substrate junction | GO:0030055 | 1.4E-05 | 3.6 |
| GO:CC | focal adhesion | GO:0005925 | 3.6E-05 | 3.5 |
| GO:CC | contractile muscle fiber | GO:0043292 | 4.2E-05 | 4.5 |
| GO:CC | actin filament bundle | GO:0032432 | 7.2E-05 | 7.5 |
| GO:CC | Z disc | GO:0030018 | 7.4E-05 | 6.1 |
| GO:CC | myofibril | GO:0030016 | 1.2E-04 | 4.4 |
| GO:CC | sarcomere | GO:0030017 | 1.8E-04 | 4.5 |
| GO:CC | extracellular region | GO:0005576 | 2.6E-04 | 1.6 |
| GO:CC | contractile actin filament bundle | GO:0097517 | 2.7E-04 | 7.5 |
| GO:CC | stress fiber | GO:0001725 | 2.7E-04 | 7.5 |
| GO:CC | I band | GO:0031674 | 2.7E-04 | 5.4 |
| GO:CC | actomyosin | GO:0042641 | 5.6E-04 | 6.9 |
| GO:CC | plasma membrane | GO:0005886 | 5.7E-04 | 1.4 |
| GO:CC | synaptic membrane | GO:0097060 | 1.9E-03 | 3.1 |
| GO:CC | postsynaptic membrane | GO:0045211 | 2.9E-03 | 3.5 |
| GO:CC | cell-cell junction | GO:0005911 | 4.3E-03 | 2.7 |
| GO:CC | vesicle | GO:0031982 | 4.4E-03 | 1.5 |
| GO:CC | postsynaptic density membrane | GO:0098839 | 6.0E-03 | 5.3 |
| GO:CC | presynapse | GO:0098793 | 6.2E-03 | 2.6 |
| GO:CC | endomembrane system | GO:0012505 | 7.1E-03 | 1.4 |
| GO:CC | postsynapse | GO:0098794 | 7.8E-03 | 2.4 |
| GO:CC | glutamatergic synapse | GO:0098978 | 9.2E-03 | 2.6 |
| GO:CC | neuron projection terminus | GO:0044306 | 9.3E-03 | 5.1 |
| GO:CC | interstitial matrix | GO:0005614 | 1.1E-02 | 7.6 |
| GO:CC | axon terminus | GO:0043679 | 1.8E-02 | 5.2 |
| GO:CC | neuron to neuron synapse | GO:0098984 | 1.9E-02 | 2.9 |
| GO:CC | extracellular space | GO:0005615 | 2.6E-02 | 1.5 |
| GO:CC | postsynaptic specialization membrane | GO:0099634 | 3.3E-02 | 4.4 |
| GO:CC | membrane | GO:0016020 | 3.8E-02 | 1.2 |
| GO:CC | postsynaptic actin cytoskeleton | GO:0098871 | 4.2E-02 | 26.7 |
| GO:CC | plasma membrane region | GO:0098590 | 4.3E-02 | 1.9 |
| GO:CC | intercalated disc | GO:0014704 | 4.4E-02 | 7.5 |
| KEGG | Cytoskeleton in muscle cells | KEGG:04820 | 3.4E-04 | 3.5 |
| KEGG | ABC transporters | KEGG:02010 | 5.1E-03 | 7.1 |
| KEGG | Regulation of actin cytoskeleton | KEGG:04810 | 1.5E-02 | 3.0 |
| REAC | Cholesterol biosynthesis | REAC:R-HSA-191273 | 5.2E-03 | 11.3 |
| REAC | Defective B3GALTL causes PpS | REAC:R-HSA-5083635 | 3.2E-02 | 8.4 |
| REAC | O-glycosylation of TSR domain-containing proteins | REAC:R-HSA-5173214 | 3.7E-02 | 8.2 |
| WP | Cholesterol metabolism with Bloch and Kandutsch Russell pathways | WP:WP4718 | 2.5E-03 | 7.2 |
| WP | Enterocyte cholesterol metabolism | WP:WP5333 | 3.6E-03 | 8.3 |
| WP | Cholesterol biosynthesis pathway | WP:WP197 | 3.6E-03 | 14.2 |
| WP | Cholesterol synthesis disorders | WP:WP5193 | 9.7E-03 | 11.8 |
| TF | Factor: MED8; motif: CYYNSCYYCCTSCNCC; match class: 1 | TF:M13128_1 | 3.4E-04 | 1.3 |
| TF | Factor: KLF15; motif: RCCMCRCCCMCN; match class: 1 | TF:M12160_1 | 6.7E-04 | 1.3 |
| TF | Factor: ZNF37A; motif: CCYYGGCTCCNTSCCMN | TF:M12354 | 1.0E-03 | 1.2 |
| TF | Factor: ZXDL; motif: GSGSCNNGGGMRGCNCCGGGS | TF:M12722 | 1.1E-03 | 1.2 |
| TF | Factor: MED8; motif: CYYNSCYYCCTSCNCC | TF:M13128 | 1.2E-02 | 1.2 |
| TF | Factor: MAZ; motif: NKGGGAGGGGRGGR | TF:M02023 | 1.6E-02 | 1.3 |
| TF | Factor: ZBTB33; motif: GGYGCTG; match class: 1 | TF:M13142_1 | 1.6E-02 | 1.6 |
| TF | Factor: SP1; motif: GGCCCCGCCCCCN; match class: 1 | TF:M12666_1 | 1.9E-02 | 1.5 |
| TF | Factor: Kaiso; motif: GCMGGGRGCRGS; match class: 1 | TF:M03876_1 | 1.9E-02 | 1.3 |
| TF | Factor: ZBTB33; motif: GGYGCTG | TF:M13142 | 1.9E-02 | 1.3 |
| TF | Factor: E2F3; motif: NNRGMKGGAR; match class: 1 | TF:M12598_1 | 2.0E-02 | 1.3 |
| TF | Factor: BTEB1; motif: GGGGGCGGGGCNGSGGGNGS | TF:M09723 | 2.1E-02 | 1.2 |
| TF | Factor: E2F-4; motif: NGGCGGGAARN; match class: 1 | TF:M07084_1 | 2.8E-02 | 1.4 |
| TF | Factor: MAZ; motif: GGGGGAGGGGGNGRGRRRGNRG; match class: 1 | TF:M09984_1 | 2.9E-02 | 1.4 |
| TF | Factor: Hey2; motif: NNCACGYGNN | TF:M11052 | 3.6E-02 | 1.2 |
| TF | Factor: BEN; motif: CAGCGRNV; match class: 1 | TF:M01240_1 | 3.7E-02 | 1.2 |
| TF | Factor: E2F-4; motif: GCGGGAAANA; match class: 1 | TF:M02090_1 | 3.7E-02 | 1.3 |
| TF | Factor: Hey1; motif: NGCRCGYGYN; match class: 1 | TF:M11058_1 | 4.7E-02 | 1.2 |
| TF | Factor: KLF15; motif: RCCMCRCCCMCN | TF:M12160 | 4.7E-02 | 1.2 |
| MIRNA | hsa-mir-124-3p | MIRNA:hsa-mir-124-3p | 1.5E-02 | 1.8 |
| MIRNA | hsa-mir-4755-5p | MIRNA:hsa-mir-4755-5p | 2.8E-02 | 1.8 |
| HPA | Endometrium; smooth muscle cells[ÔëÑLow] | HPA:0640691 | 2.4E-03 | 6.5 |
| HPA | Endometrium; smooth muscle cells[High] | HPA:0640693 | 5.0E-03 | 10.5 |
| HPA | Endometrium | HPA:0640000 | 1.3E-02 | 4.3 |
| HPA | Endometrium; smooth muscle cells[ÔëÑMedium] | HPA:0640692 | 1.6E-02 | 7.0 |
| CORUM | CTGF/Hcs24-actin(beta/gamma) complex | CORUM:2254 | 5.0E-02 | 57.3 |
| CORUM | FGF1-FGFR1-ITGB3 complex | CORUM:6909 | 5.0E-02 | 57.3 |
| HP | Gastrointestinal dysmotility | HP:0002579 | 2.5E-02 | 4.4 |
| HP | Abnormal gastrointestinal motility | HP:0030895 | 3.0E-02 | 4.3 |

Supplementary Table 6 FPM of Monocarboxylate transporters and Basigin in HK-2 cells.

| **Gene name** | **Corresponding protein** | **mean ± CI_95_ pH 7.4** | **mean ± CI_95_ lactic acid pH 6.4** |
| --- | --- | --- | --- |
| SLC16A1 | MCT1 | 354.8 ± (327.7; 381.9) | 289.6 (250.6; 328.6) |
| SLC16A7 | MCT2 | 27 ± (22.5; 31.5) | 16.8 ± (13.9; 19.7) |
| SLC16A3 | MCT4 | 134.4 ± (109.6; 159:2) | 236.0 ± (195.6; 276.4) |
| BSG | Basigin | 7.0 ± (5.0; 9.0) | 4.4 ± (2.7; 6.1) |

Supplementary Table 7 FPM of Monocarboxylate transporters and Basigin in CCDSk cells.

| **Gene name** | **Corresponding protein** | **mean ± CI_95_ pH 7.4** | **mean ± CI_95_ lactic acid pH 6.4** |
| --- | --- | --- | --- |
| SLC16A1 | MCT1 | 32.6 ± (30.1; 35.1) | 63.2 (39.4; 87.0) |
| SLC16A7 | MCT2 | 23.4 ± (21.5; 25.4) | 25.2 ± (18.2; 32.2) |
| SLC16A3 | MCT4 | 63.8 ± (55.0; 72.6) | 104.2 ± (50.6; 157.8) |
| BSG | Basigin | 454.4 ± (454.4; 479.3) | 1080.4 ± (826.8; 1334.0) |

Supplementary Table 8 Overview over the comparative analysis of canonical pathway analysis obtained for genes regulated in HK-2 cells or CCDSk cells after incubation with HCl or lactic acid. Red field marks a predicted activation (z-score > 3) and the blue field marks a predicted inhibition (z-score < -3).

| **Canonical Pathways** | **HK2_mono_HCl** | **HK2_mono_LA** | **CCDSK_mono_HCl** | **CCDSK_mono_LA** |
| --- | --- | --- | --- | --- |
| 3-phosphoinositide Biosynthesis | N/A | 0.7 | N/A | 1.1 |
| 3-phosphoinositide Degradation | N/A | 0.7 | N/A | 2.1 |
| ABC-family proteins mediated transport | N/A | -1.0 | 0.4 | -0.6 |
| ABRA Signaling Pathway | N/A | 1.7 | 0.8 | 3.3 |
| Actin Cytoskeleton Signaling | N/A | 1.3 | 2.5 | 3.1 |
| Actin Nucleation by ARP-WASP Complex | N/A | 1.1 | N/A | 4.0 |
| Activation of gene expression by SREBF (SREBP) | -3.3 | -3.0 | 2.2 | 0.7 |
| Activation of NMDA receptors and postsynaptic events | -1.0 | -1.5 | N/A | 0.2 |
| Activation of the pre-replicative complex | -2.4 | -4.8 | N/A | 0.5 |
| Activin Inhibin Signaling Pathway | N/A | 2.5 | -0.8 | -0.9 |
| Acute Myeloid Leukemia Signaling | N/A | 0.3 | N/A | -0.5 |
| Acute Phase Response Signaling | N/A | 2.6 | 0.0 | 1.6 |
| Adrenomedullin signaling pathway | N/A | 1.4 | N/A | 1.8 |
| Aggrephagy | N/A | -1.9 | N/A | 2.8 |
| Amino acids regulate mTORC1 | N/A | -3.9 | N/A | 1.8 |
| AMPK Signaling | 1.3 | 1.7 | 2.0 | -2.8 |
| Androgen Signaling | N/A | 0.7 | N/A | 0.6 |
| Angiopoietin Signaling | N/A | 0.7 | N/A | 1.5 |
| Apelin Endothelial Signaling Pathway | N/A | 1.8 | N/A | 0.9 |
| Apelin Pancreas Signaling Pathway | N/A | 0.6 | N/A | -2.8 |
| Aryl Hydrocarbon Receptor Signaling | -2.0 | -1.1 | N/A | 0.6 |
| Assembly and cell surface presentation of NMDA receptors | N/A | 0.3 | N/A | 1.4 |
| Assembly of collagen fibrils and other multimeric structures | N/A | 4.0 | 1.0 | 1.3 |
| Assembly of RNA Polymerase II Complex | N/A | -1.9 | N/A | -0.4 |
| ATF4 activates genes in response to endoplasmic reticulum stress | N/A | 1.6 | N/A | 2.7 |
| ATM Signaling | -0.4 | -1.3 | N/A | -1.9 |
| Autophagy | N/A | -2.1 | N/A | 2.4 |
| B Cell Activating Factor Signaling | N/A | 0.4 | N/A | 1.6 |
| BER (Base Excision Repair) Pathway | N/A | -2.8 | N/A | 0.5 |
| Beta-catenin independent WNT signaling | N/A | -0.5 | -2.0 | 0.1 |
| BMAL1:CLOCK,NPAS2 activates circadian gene expression | N/A | 1.9 | N/A | 0.5 |
| BMP signaling pathway | N/A | -0.3 | N/A | 0.3 |
| Branched-chain amino acid catabolism | N/A | -2.0 | N/A | -2.7 |
| B-WICH complex positively regulates rRNA expression | N/A | -2.1 | N/A | 2.4 |
| Cachexia Signaling Pathway | N/A | 1.3 | -0.4 | 3.5 |
| Cancer Drug Resistance by Drug Efflux | N/A | 1.0 | N/A | 1.8 |
| Carboxyterminal post-translational modifications of tubulin | N/A | -1.1 | N/A | 1.4 |
| Cardiac Hypertrophy Signaling | N/A | 2.2 | 1.9 | 1.5 |
| Cargo recognition for clathrin-mediated endocytosis | N/A | 0.0 | N/A | 2.1 |
| Caveolar-mediated Endocytosis Signaling | N/A | 4.2 | 1.9 | 1.5 |
| CDK5 Signaling | N/A | 1.2 | N/A | 3.5 |
| Cell Cycle Checkpoints | -6.7 | -7.5 | N/A | 1.2 |
| Cell Cycle Control of Chromosomal Replication | -2.6 | -4.1 | N/A | 0.4 |
| Cell Cycle Regulation by BTG Family Proteins | N/A | -2.3 | N/A | 2.3 |
| Cellular Effects of Sildenafil (Viagra) | 0.8 | -4.0 | 0.2 | -0.7 |
| Cellular hexose transport | N/A | 2.3 | N/A | 1.1 |
| Cellular response to heat stress | N/A | -1.0 | N/A | 3.4 |
| Cellular response to hypoxia | N/A | -2.1 | N/A | 3.1 |
| Ceramide Signaling | N/A | 0.8 | N/A | 5.0 |
| CGAS-STING Signaling Pathway | N/A | -1.6 | N/A | 4.3 |
| Cholecystokinin/Gastrin-mediated Signaling | N/A | 1.4 | 1.0 | 3.0 |
| Cholesterol biosynthesis | -3.3 | -4.1 | 2.4 | 2.5 |
| Cholesterol Biosynthesis I | -2.0 | -3.0 | 2.0 | 2.3 |
| Cholesterol Biosynthesis II (via 24,25-dihydrolanosterol) | -2.0 | -3.0 | 2.0 | 2.3 |
| Cholesterol Biosynthesis III (via Desmosterol) | -2.0 | -3.0 | 2.0 | 2.3 |
| Chromatin organization | N/A | -4.9 | N/A | 1.1 |
| Chronic Myeloid Leukemia Signaling | -2.6 | 1.4 | -0.4 | 1.9 |
| Cilium Assembly | -2.2 | -0.7 | 0.0 | -2.9 |
| Circadian Clock | N/A | 0.9 | N/A | 0.8 |
| Class I MHC mediated antigen processing and presentation | N/A | -0.9 | N/A | 1.9 |
| Class I peroxisomal membrane protein import | N/A | -1.0 | N/A | -2.1 |
| Clathrin-mediated endocytosis | N/A | 0.0 | 2.0 | 2.8 |
| Cohesin Chromatin Regulation Pathway | -2.4 | -3.0 | N/A | -1.0 |
| Collagen biosynthesis and modifying enzymes | N/A | 3.3 | -0.4 | 2.0 |
| Collagen chain trimerization | N/A | 2.5 | 0.0 | 0.6 |
| Collagen degradation | N/A | 4.1 | 0.0 | 1.1 |
| Complex I biogenesis | N/A | -3.2 | N/A | -0.2 |
| Complex IV assembly | N/A | -0.8 | N/A | 2.4 |
| COPII-mediated vesicle transport | N/A | 1.3 | N/A | -1.8 |
| COPI-mediated anterograde transport | N/A | -0.7 | N/A | -0.3 |
| Coronavirus Replication Pathway | N/A | -1.7 | N/A | 1.5 |
| Corticotropin Releasing Hormone Signaling | N/A | -0.6 | N/A | 1.7 |
| Cristae formation | N/A | -2.1 | N/A | 3.3 |
| CSDE1 Signaling Pathway | N/A | 0.0 | N/A | 1.6 |
| C-type lectin receptors (CLRs) | N/A | -1.7 | N/A | 2.6 |
| CXCR4 Signaling | N/A | 1.2 | N/A | 1.9 |
| Cyclins and Cell Cycle Regulation | -2.6 | -2.4 | N/A | 0.6 |
| Cyclophilin Signaling Pathway | N/A | 0.0 | N/A | 2.1 |
| Cytoprotection by HMOX1 | N/A | 0.7 | N/A | 2.9 |
| Cytosolic sensors of pathogen-associated DNA | N/A | -1.4 | N/A | 0.3 |
| DAG and IP3 signaling | N/A | -1.5 | N/A | -0.5 |
| Deadenylation-dependent mRNA decay | N/A | -2.9 | N/A | 1.5 |
| Degradation of beta-catenin by the destruction complex | N/A | -2.7 | N/A | 1.2 |
| Degradation of the extracellular matrix | N/A | 3.9 | 1.0 | 3.1 |
| Deubiquitination | -2.1 | -3.8 | N/A | 1.1 |
| D-myo-inositol (1,4,5,6)-Tetrakisphosphate Biosynthesis | N/A | 1.3 | N/A | 2.3 |
| D-myo-inositol (3,4,5,6)-tetrakisphosphate Biosynthesis | N/A | 1.3 | N/A | 2.3 |
| D-myo-inositol-5-phosphate Metabolism | N/A | 1.2 | N/A | 2.1 |
| DNA Damage Bypass | N/A | -2.5 | N/A | 1.3 |
| DNA Damage/Telomere Stress Induced Senescence | -0.8 | -2.5 | N/A | 2.0 |
| DNA Double Strand Break Response | -2.0 | -2.1 | N/A | 1.7 |
| DNA Methylation and Transcriptional Repression Signaling | -2.0 | -1.7 | N/A | 3.3 |
| DNA Replication Pre-Initiation | -1.6 | -5.1 | N/A | 2.7 |
| Docosahexaenoic Acid (DHA) Signaling | N/A | 1.5 | 0.0 | 1.1 |
| EGF Signaling | N/A | 0.6 | N/A | 0.2 |
| EGR2 and SOX10-mediated initiation of Schwann cell myelination | N/A | 0.4 | N/A | 1.1 |
| Eicosanoid Signaling | N/A | 2.8 | -0.3 | 2.6 |
| EIF2 Signaling | N/A | 1.0 | N/A | 2.8 |
| Elastic fibre formation | N/A | 4.0 | N/A | 0.4 |
| Endocannabinoid Developing Neuron Pathway | N/A | 1.3 | N/A | 1.3 |
| Endometrial Cancer Signaling | N/A | 0.0 | N/A | -0.2 |
| Endothelin-1 Signaling | N/A | 1.6 | N/A | 0.7 |
| EPH-Ephrin signaling | N/A | -1.1 | 1.6 | 2.1 |
| Ephrin Receptor Signaling | N/A | 2.6 | 0.0 | 3.7 |
| Epithelial Adherens Junction Signaling | -1.6 | -1.3 | 1.0 | 0.0 |
| ERB2-ERBB3 Signaling | N/A | 1.1 | N/A | 0.0 |
| ERBB Signaling | N/A | 2.2 | N/A | 1.2 |
| ERBB4 Signaling | N/A | 0.7 | N/A | -0.2 |
| ERCC6 (CSB) and EHMT2 (G9a) positively regulate rRNA expression | N/A | -2.6 | N/A | 3.2 |
| ERK/MAPK Signaling | N/A | 1.3 | N/A | 1.9 |
| ERK5 Signaling | N/A | 0.5 | N/A | 2.5 |
| ESR-mediated signaling | -1.0 | -2.5 | N/A | 3.3 |
| Estrogen Receptor Signaling | N/A | 2.3 | -1.1 | 1.2 |
| Estrogen-Dependent Breast Cancer Signaling | N/A | 0.3 | N/A | 1.4 |
| Estrogen-mediated S-phase Entry | -2.2 | -2.1 | N/A | 1.5 |
| Eukaryotic Translation Elongation | N/A | -1.0 | N/A | 9.0 |
| Eukaryotic Translation Initiation | N/A | -0.8 | N/A | 9.4 |
| Eukaryotic Translation Termination | N/A | -1.0 | N/A | 8.5 |
| Extracellular matrix organization | N/A | 5.5 | 1.1 | 2.5 |
| Extra-nuclear estrogen signaling | N/A | 2.0 | N/A | 2.2 |
| Factors Promoting Cardiogenesis in Vertebrates | N/A | 3.0 | N/A | -1.4 |
| Fanconi Anemia Pathway | N/A | -1.6 | N/A | 0.2 |
| FAT10 Cancer Signaling Pathway | N/A | 1.6 | N/A | 0.4 |
| Fcγ Receptor-mediated Phagocytosis in Macrophages and Monocytes | N/A | 0.2 | 2.0 | 1.2 |
| Ferroptosis Signaling Pathway | 0.8 | 2.7 | N/A | 0.5 |
| FGF Signaling | N/A | 1.0 | N/A | -0.8 |
| FLT3 Signaling | N/A | 1.0 | N/A | 1.9 |
| FLT3 Signaling in Hematopoietic Progenitor Cells | N/A | 0.3 | N/A | -0.2 |
| fMLP Signaling in Neutrophils | N/A | 0.3 | N/A | 1.9 |
| Folate Signaling Pathway | N/A | -1.4 | N/A | 1.2 |
| Formation of paraxial mesoderm | N/A | 2.2 | N/A | 1.1 |
| Formation of WDR5-containing histone-modifying complexes | N/A | -2.6 | N/A | -0.7 |
| FOXO-mediated transcription of oxidative stress, metabolic and neuronal genes | N/A | 1.0 | N/A | -0.3 |
| G alpha (12/13) signalling events | N/A | 1.4 | 0.4 | -1.5 |
| G alpha (q) signalling events | N/A | 2.7 | -1.3 | 0.4 |
| G Beta Gamma Signaling | N/A | 2.6 | N/A | 2.0 |
| Gamma carboxylation, hypusinylation, hydroxylation, and arylsulfatase activation | N/A | -0.9 | N/A | 2.0 |
| Gap Junction Signaling | N/A | -0.1 | 1.7 | 1.8 |
| Gap junction trafficking and regulation | N/A | -1.7 | N/A | 3.0 |
| GDNF Family Ligand-Receptor Interactions | N/A | 1.7 | N/A | 1.0 |
| Gene Silencing by RNA | N/A | -3.0 | N/A | 0.1 |
| Generic Transcription Pathway | N/A | 2.7 | N/A | -9.2 |
| Glioma Invasiveness Signaling | N/A | 2.3 | N/A | 1.5 |
| Glucose metabolism | N/A | 1.2 | N/A | 2.0 |
| Glutaminergic Receptor Signaling Pathway (Enhanced) | -0.8 | 2.6 | 0.7 | 0.3 |
| Glycation Signaling Pathway | N/A | 1.9 | -0.4 | 2.9 |
| Glycerophospholipid biosynthesis | N/A | 2.0 | 1.0 | 1.6 |
| Glycosaminoglycan metabolism | N/A | 1.0 | 0.4 | 1.9 |
| GM-CSF Signaling | N/A | 0.3 | N/A | 0.7 |
| GNRH Signaling | N/A | 1.2 | 0.0 | 2.6 |
| GPER1 signaling | N/A | 0.4 | N/A | 0.3 |
| Granzyme A Signaling | 1.0 | 2.5 | N/A | -1.0 |
| Granzyme B Signaling | N/A | -2.0 | N/A | -1.6 |
| Growth Hormone Signaling | N/A | 0.3 | N/A | -3.0 |
| Gα12/13 Signaling | N/A | 0.2 | 0.0 | 1.8 |
| Gαq Signaling | N/A | 1.7 | -0.4 | -0.7 |
| HDR through Homologous Recombination (HRR) or Single Strand Annealing (SSA) | -3.9 | -5.3 | N/A | 0.7 |
| HDR through MMEJ (alt-NHEJ) | N/A | -2.2 | N/A | N/A |
| Hedgehog ligand biogenesis | N/A | -2.5 | N/A | 1.2 |
| Hedgehog 'off' state | N/A | -3.1 | N/A | 1.1 |
| Hedgehog 'on' state | N/A | -1.6 | N/A | -0.7 |
| Hematoma Resolution Signaling Pathway | N/A | -2.1 | N/A | -1.3 |
| Heme signaling | N/A | -1.1 | N/A | -0.2 |
| Hepatic Cholestasis | N/A | 1.0 | N/A | 2.7 |
| Hepatic Fibrosis Signaling Pathway | N/A | 4.9 | 1.3 | 1.3 |
| Hepatitis B Chronic Liver Pathogenesis Signaling Pathway | 0.4 | 0.7 | N/A | 2.4 |
| HER-2 Signaling in Breast Cancer | N/A | 2.7 | -1.0 | 0.7 |
| Hereditary Breast Cancer Signaling | 2.1 | 2.6 | N/A | -0.3 |
| HGF Signaling | N/A | 2.7 | N/A | 1.3 |
| HIF1α Signaling | N/A | 1.9 | 1.6 | 3.0 |
| HIPPO signaling | N/A | -0.3 | N/A | -1.4 |
| Histone Modification Signaling Pathway | -2.3 | -4.1 | N/A | 2.4 |
| HMGB1 Signaling | N/A | 1.7 | N/A | 3.2 |
| HOTAIR Regulatory Pathway | N/A | 1.9 | N/A | 0.3 |
| HSP90 chaperone cycle for steroid hormone receptors in the presence of ligand | N/A | -2.3 | N/A | 3.3 |
| Human Embryonic Stem Cell Pluripotency | N/A | 2.9 | 0.4 | 0.8 |
| Huntington's Disease Signaling | N/A | 2.2 | N/A | -0.1 |
| ID1 Signaling Pathway | -0.4 | 1.0 | 0.0 | 1.4 |
| IGF-1 Signaling | N/A | 0.0 | N/A | 0.1 |
| IL-1 Signaling | N/A | 0.6 | N/A | 2.1 |
| IL-17A Signaling in Airway Cells | N/A | 1.3 | N/A | -2.0 |
| IL-17A Signaling in Fibroblasts | N/A | 2.6 | N/A | 2.4 |
| IL-2 Signaling | N/A | 1.4 | N/A | 0.0 |
| IL-3 Signaling | N/A | 0.9 | N/A | 0.5 |
| IL-33 Signaling Pathway | N/A | 0.9 | N/A | 0.9 |
| IL-6 Signaling | N/A | 2.4 | 1.3 | 2.2 |
| IL-7 Signaling Pathway | N/A | 2.1 | N/A | -1.1 |
| IL-8 Signaling | N/A | 2.9 | -1.1 | 2.6 |
| ILK Signaling | N/A | 1.0 | 1.9 | 1.5 |
| Immunogenic Cell Death Signaling Pathway | N/A | 0.6 | N/A | 2.5 |
| Inhibition of ARE-Mediated mRNA Degradation Pathway | N/A | 0.0 | N/A | 0.1 |
| iNOS Signaling | N/A | 0.8 | N/A | 0.9 |
| Insertion of tail-anchored proteins into the endoplasmic reticulum membrane | N/A | 0.4 | N/A | 2.7 |
| Insulin Secretion Signaling Pathway | N/A | 1.2 | N/A | 0.5 |
| Integrin cell surface interactions | N/A | 5.0 | N/A | 2.7 |
| Integrin Signaling | N/A | 3.3 | 2.1 | 2.1 |
| Integrin signaling | N/A | 1.9 | N/A | 0.3 |
| Interconversion of nucleotide di- and triphosphates | N/A | -0.6 | N/A | 2.3 |
| Interferon alpha/beta signaling | N/A | 3.2 | N/A | 2.3 |
| Interferon gamma signaling | N/A | 2.8 | N/A | 3.6 |
| Interleukin-1 family signaling | N/A | -1.1 | N/A | 2.0 |
| Interleukin-10 signaling | N/A | 2.1 | N/A | 3.8 |
| Interleukin-3, Interleukin-5 and GM-CSF signaling | N/A | 0.0 | N/A | 1.3 |
| Interleukin-6 family signaling | N/A | 2.1 | N/A | 1.9 |
| Intra-Golgi and retrograde Golgi-to-ER traffic | -3.1 | -0.5 | N/A | -0.3 |
| Intrinsic Pathway for Apoptosis | N/A | 0.0 | N/A | 1.3 |
| Ion channel transport | N/A | 0.6 | N/A | 1.3 |
| Iron uptake and transport | N/A | -2.3 | N/A | 3.9 |
| ISG15 antiviral mechanism | N/A | -0.6 | N/A | 0.9 |
| JAK/STAT Signaling | N/A | 1.9 | N/A | -0.6 |
| KEAP1-NFE2L2 pathway | N/A | -2.8 | N/A | 1.2 |
| Kinesins | -3.1 | -0.3 | N/A | 1.2 |
| Kinetochore Metaphase Signaling Pathway | -3.0 | -2.3 | N/A | 1.3 |
| L1CAM interactions | -1.0 | 1.4 | 1.1 | 3.3 |
| Late endosomal microautophagy | N/A | -0.4 | N/A | 2.4 |
| LPS-stimulated MAPK Signaling | N/A | 0.6 | N/A | 1.5 |
| LXR/RXR Activation | N/A | 0.0 | N/A | -1.1 |
| Macroautophagy | N/A | -1.5 | N/A | 1.5 |
| Macropinocytosis Signaling | N/A | 0.9 | N/A | 1.0 |
| Major pathway of rRNA processing in the nucleolus and cytosol | N/A | -5.1 | N/A | 7.0 |
| MAP kinase activation | N/A | 0.4 | N/A | 1.6 |
| MAPK targets/ Nuclear events mediated by MAP kinases | N/A | -0.8 | N/A | 2.1 |
| MAPK6/MAPK4 signaling | N/A | 1.6 | N/A | 2.7 |
| Maturation of TCA enzymes and regulation of TCA cycle | N/A | -1.0 | N/A | -1.2 |
| M-decay: degradation of maternal mRNAs by maternally stored factors | N/A | -1.4 | N/A | 2.3 |
| Meiotic recombination | -1.9 | -3.9 | N/A | 1.2 |
| Metabolism of cofactors | N/A | -2.2 | N/A | -1.6 |
| Metabolism of folate and pterines | N/A | -1.0 | N/A | 0.4 |
| Metabolism of non-coding RNA | N/A | -3.5 | N/A | -1.4 |
| Metabolism of polyamines | N/A | -2.7 | N/A | 2.4 |
| Metabolism of water-soluble vitamins and cofactors | N/A | -1.1 | N/A | -1.4 |
| Mevalonate Pathway I | -2.0 | -2.2 | N/A | N/A |
| MHC class II antigen presentation | -3.0 | -1.1 | N/A | 2.7 |
| Microautophagy Signaling Pathway | N/A | -2.0 | N/A | 0.4 |
| MicroRNA Biogenesis Signaling Pathway | N/A | -1.8 | N/A | -1.4 |
| MIF Regulation of Innate Immunity | N/A | 1.6 | N/A | 2.5 |
| Mismatch Repair | N/A | -1.6 | N/A | 0.0 |
| Mismatch Repair in Eukaryotes | N/A | -3.2 | N/A | 1.0 |
| MITF-M-dependent gene expression | N/A | -2.0 | N/A | 2.4 |
| Mitochondrial biogenesis | N/A | -0.5 | N/A | -0.4 |
| Mitochondrial Division Signaling Pathway | -1.1 | 2.1 | 1.3 | 2.2 |
| Mitochondrial Dysfunction | N/A | 1.2 | N/A | -2.9 |
| Mitochondrial Fatty Acid Beta-Oxidation | N/A | -1.0 | N/A | -2.2 |
| Mitochondrial protein degradation | N/A | -2.8 | N/A | 1.3 |
| Mitochondrial protein import | N/A | -2.2 | N/A | 2.5 |
| Mitochondrial translation | N/A | -3.3 | N/A | 1.5 |
| Mitophagy | N/A | -0.8 | N/A | 2.7 |
| Mitotic G1 phase and G1/S transition | 2.8 | 0.2 | N/A | 2.4 |
| Mitotic G2-G2/M phases | -4.1 | -4.3 | 0.0 | 3.1 |
| Mitotic Metaphase and Anaphase | -5.7 | -6.2 | N/A | 1.5 |
| Mitotic Prometaphase | -5.8 | -4.8 | N/A | 0.7 |
| Mitotic Prophase | -2.5 | -4.0 | N/A | 0.6 |
| Mitotic Roles of Polo-Like Kinase | -1.9 | -0.8 | N/A | -1.5 |
| Mouse Embryonic Stem Cell Pluripotency | N/A | 1.5 | N/A | -0.3 |
| mRNA 3 Prime End Processing Signaling Pathway | 0.0 | -2.2 | N/A | 1.8 |
| mRNA Capping | N/A | -1.0 | N/A | -0.7 |
| MSP-RON Signaling in Cancer Cells Pathway | N/A | 1.5 | N/A | 2.8 |
| MTOR signalling | N/A | -2.2 | N/A | 1.0 |
| NAD Signaling Pathway | N/A | 2.2 | 1.3 | 0.0 |
| NAFLD Signaling Pathway | -1.4 | 1.1 | -0.4 | 3.4 |
| NAP1L1 Transcription Regulation Signaling Pathway | N/A | -2.1 | N/A | 1.9 |
| Neddylation | N/A | -3.3 | N/A | 2.1 |
| NER (Nucleotide Excision Repair, Enhanced Pathway) | N/A | -3.0 | N/A | -1.0 |
| Netrin Signaling | N/A | 1.3 | 0.4 | 1.8 |
| Netrin-1 signaling | N/A | 0.4 | N/A | -0.2 |
| Neuregulin Signaling | N/A | 1.3 | N/A | 1.2 |
| Neurexins and neuroligins | N/A | 2.8 | N/A | -1.0 |
| Neuroinflammation Signaling Pathway | N/A | 2.0 | 1.0 | 0.3 |
| Neurotrophin/TRK Signaling | N/A | 0.0 | N/A | 1.6 |
| Neutrophil degranulation | N/A | 0.1 | 0.4 | 6.4 |
| NF-κB Activation by Viruses | N/A | 1.2 | N/A | 0.2 |
| NGF Signaling | N/A | -0.2 | N/A | 1.0 |
| NGF-stimulated transcription | 0.0 | 0.3 | N/A | 4.4 |
| NIK-->noncanonical NF-kB signaling | N/A | -2.3 | N/A | 2.0 |
| NLR signaling pathways | N/A | 0.9 | N/A | 2.5 |
| NOD1/2 Signaling Pathway | N/A | 1.8 | 0.0 | 2.0 |
| Nonhomologous End-Joining (NHEJ) | N/A | -2.5 | N/A | 0.9 |
| Nonsense-Mediated Decay (NMD) | N/A | -2.1 | N/A | 8.5 |
| Non-Small Cell Lung Cancer Signaling | N/A | 0.9 | N/A | 0.2 |
| NoRC negatively regulates rRNA expression | N/A | -1.7 | N/A | 1.9 |
| NRF2-mediated Oxidative Stress Response | N/A | 0.0 | N/A | 3.6 |
| Nuclear Cytoskeleton Signaling Pathway | -3.4 | 1.0 | 1.0 | 1.2 |
| Nucleosome assembly | -2.5 | -3.5 | N/A | 2.7 |
| Nucleotide Excision Repair | N/A | -3.8 | N/A | -0.3 |
| Nucleotide salvage | N/A | 0.0 | N/A | 2.7 |
| Oncostatin M Signaling | N/A | 1.7 | N/A | 2.2 |
| Opioid Signaling Pathway | N/A | 1.3 | -0.8 | 2.3 |
| Opioid Signalling | N/A | -0.3 | N/A | 0.6 |
| Oxidative Phosphorylation | N/A | -2.0 | N/A | 4.0 |
| Oxidative Stress Induced Senescence | N/A | -1.3 | N/A | 3.3 |
| Oxytocin in Brain Signaling Pathway | N/A | 2.4 | 0.7 | 1.1 |
| Oxytocin Signaling Pathway | N/A | 2.1 | 1.5 | 2.7 |
| P2Y Purinergic Receptor Signaling Pathway | N/A | 1.3 | N/A | 0.6 |
| p38 MAPK Signaling | N/A | 1.1 | 1.3 | 2.9 |
| p75 NTR receptor-mediated signalling | -2.0 | -0.9 | N/A | 0.6 |
| PAK Signaling | N/A | 2.4 | N/A | 1.1 |
| Pancreatic Secretion Signaling Pathway | N/A | 2.2 | 0.4 | -0.8 |
| Paxillin Signaling | N/A | 1.4 | 2.4 | 1.1 |
| PCP (Planar Cell Polarity) Pathway | N/A | 2.3 | N/A | 0.0 |
| PDGF Signaling | N/A | 1.4 | N/A | 1.2 |
| Peroxisomal protein import | N/A | 0.4 | N/A | -1.6 |
| PFKFB4 Signaling Pathway | N/A | 0.0 | N/A | 1.3 |
| PI Metabolism | N/A | -1.4 | N/A | -2.6 |
| PKR-mediated signaling | N/A | -2.2 | N/A | 2.8 |
| Plasma lipoprotein assembly, remodeling, and clearance | 0.0 | -0.3 | N/A | 3.2 |
| Polyamine Regulation in Colon Cancer | N/A | 0.0 | N/A | 1.2 |
| PPAR Signaling | N/A | -2.5 | N/A | -2.2 |
| PPARα/RXRα Activation | N/A | -0.7 | N/A | -1.2 |
| Pre-NOTCH Expression and Processing | N/A | 0.0 | N/A | 3.3 |
| Processing of Capped Intron-Containing Pre-mRNA | N/A | -8.7 | N/A | 1.8 |
| Processing of Capped Intronless Pre-mRNA | N/A | -3.2 | N/A | 1.0 |
| Production of Nitric Oxide and Reactive Oxygen Species in Macrophages | N/A | 2.3 | N/A | 0.4 |
| Prolactin Signaling | N/A | 1.3 | N/A | -0.7 |
| Prostate Cancer Signaling | N/A | -0.7 | N/A | 1.6 |
| Protein folding | N/A | -1.2 | 0.4 | 4.0 |
| Protein Kinase A Signaling | 1.3 | 0.8 | -0.7 | 1.5 |
| Protein Sorting Signaling Pathway | N/A | -0.5 | N/A | 1.1 |
| Protein Ubiquitination Pathway | N/A | -1.5 | N/A | 2.0 |
| PTEN Regulation | N/A | -2.1 | N/A | 1.1 |
| PTEN Signaling | N/A | -1.3 | N/A | -0.8 |
| Pulmonary Fibrosis Idiopathic Signaling Pathway | N/A | 4.3 | 0.6 | 0.4 |
| Pulmonary Healing Signaling Pathway | N/A | 2.2 | 0.4 | 1.1 |
| Pyridoxal 5'-phosphate Salvage Pathway | -2.0 | 1.3 | N/A | 0.2 |
| RAB geranylgeranylation | N/A | 0.0 | N/A | 1.9 |
| RAC Signaling | N/A | 0.0 | N/A | 2.0 |
| RANK Signaling in Osteoclasts | N/A | 1.5 | 1.0 | 0.3 |
| Reelin Signaling in Neurons | N/A | 2.9 | 0.0 | 0.6 |
| Regulation of Actin-based Motility by Rho | N/A | 1.2 | 1.0 | 4.0 |
| Regulation of Apoptosis | N/A | -2.9 | N/A | 1.5 |
| Regulation of Cellular Mechanics by Calpain Protease | N/A | 1.5 | N/A | 2.7 |
| Regulation of mitotic cell cycle | -3.5 | -3.6 | N/A | 1.3 |
| Regulation of mRNA stability by proteins that bind AU-rich elements | N/A | -2.2 | N/A | 2.4 |
| Regulation of RUNX2 expression and activity | N/A | -1.3 | N/A | 2.1 |
| Regulation of the Epithelial Mesenchymal Transition by Growth Factors Pathway | N/A | 2.7 | -0.4 | 1.8 |
| Regulation of TP53 Activity through Acetylation | N/A | -2.1 | N/A | 0.8 |
| Regulation of TP53 Activity through Phosphorylation | -3.3 | -3.1 | N/A | 0.9 |
| Relaxin Signaling | N/A | 0.5 | N/A | -0.4 |
| Remodeling of Epithelial Adherens Junctions | N/A | -0.4 | N/A | 1.9 |
| Renal Cell Carcinoma Signaling | N/A | 1.0 | N/A | 0.8 |
| Renin-Angiotensin Signaling | N/A | 1.3 | N/A | 1.0 |
| Resolution of Abasic Sites (AP sites) | N/A | -2.7 | N/A | -0.3 |
| Respiratory electron transport | N/A | -2.3 | N/A | 2.6 |
| Response of EIF2AK4 (GCN2) to amino acid deficiency | N/A | 0.4 | N/A | 8.9 |
| Response to elevated platelet cytosolic Ca2+ | N/A | 1.3 | -0.4 | 3.2 |
| RHO GTPase cycle | -2.9 | 0.8 | 1.4 | -0.1 |
| RHO GTPases Activate Formins | -4.9 | -3.7 | N/A | 2.1 |
| RHO GTPases activate IQGAPs | N/A | -1.9 | N/A | 1.6 |
| RHO GTPases activate PKNs | N/A | -1.0 | N/A | 1.7 |
| RHO GTPases Activate ROCKs | N/A | -1.0 | N/A | 0.9 |
| RHO GTPases Activate WASPs and WAVEs | N/A | -0.8 | N/A | 2.8 |
| RHOA Signaling | N/A | 0.6 | 1.5 | 2.1 |
| RHOGDI Signaling | N/A | -3.1 | -1.4 | -1.9 |
| Ribonucleotide Reductase Signaling Pathway | -1.1 | 0.2 | 0.0 | -0.1 |
| Ribosomal Quality Control Signaling Pathway | N/A | -3.1 | N/A | 7.0 |
| RNA Polymerase I Transcription | N/A | -2.5 | N/A | 2.1 |
| RNA Polymerase II Transcription | N/A | -5.3 | N/A | 2.1 |
| RNA Polymerase III Transcription | N/A | -0.4 | N/A | -1.0 |
| ROBO SLIT Signaling Pathway | N/A | 0.0 | N/A | -2.3 |
| Role of BRCA1 in DNA Damage Response | -0.7 | -0.7 | N/A | -1.1 |
| Role of CHK Proteins in Cell Cycle Checkpoint Control | 1.9 | 1.2 | N/A | 0.2 |
| Role of Chondrocytes in Rheumatoid Arthritis Signaling Pathway | N/A | 3.0 | N/A | 2.3 |
| Role of IL-17F in Allergic Inflammatory Airway Diseases | N/A | 0.3 | N/A | 2.3 |
| Role of JAK family kinases in IL-6-type Cytokine Signaling | N/A | 2.2 | N/A | 2.3 |
| Role of JAK1 and JAK3 in γc Cytokine Signaling | N/A | 2.3 | N/A | 0.0 |
| Role of MAPK Signaling in Inhibiting the Pathogenesis of Influenza | N/A | 1.7 | N/A | 0.4 |
| Role of MAPK Signaling in Promoting the Pathogenesis of Influenza | N/A | -1.3 | 1.0 | 2.9 |
| Role of NANOG in Mammalian Embryonic Stem Cell Pluripotency | N/A | 1.7 | N/A | -0.2 |
| Role of NFAT in Cardiac Hypertrophy | N/A | 2.0 | -0.4 | 1.1 |
| Role of Osteoblasts in Rheumatoid Arthritis Signaling Pathway | N/A | -0.4 | 0.0 | 4.5 |
| Role of Tissue Factor in Cancer | -1.0 | 1.6 | -0.4 | 2.7 |
| rRNA modification in the nucleus and cytosol | N/A | -4.1 | N/A | 0.3 |
| S Phase | N/A | -3.8 | N/A | 2.1 |
| Salvage Pathways of Pyrimidine Ribonucleotides | -2.0 | 1.4 | 1.6 | 0.8 |
| Selenoamino acid metabolism | N/A | -0.4 | N/A | 8.0 |
| Semaphorin interactions | N/A | 0.5 | 1.0 | -0.5 |
| Senescence Pathway | 2.6 | 2.2 | 0.0 | 1.1 |
| Senescence-Associated Secretory Phenotype (SASP) | -1.0 | -2.1 | N/A | 3.8 |
| Serotonin Receptor Signaling | -0.4 | 2.8 | 1.4 | 2.5 |
| Sertoli Cell-Sertoli Cell Junction Signaling | N/A | 1.0 | 1.9 | 2.9 |
| Sheddase Signaling Pathway | N/A | 2.9 | -1.9 | 2.1 |
| Signaling by BMP | N/A | 2.3 | N/A | -0.5 |
| Signaling by CSF1 (M-CSF) in myeloid cells | N/A | 1.3 | N/A | 0.7 |
| Signaling by EGFR | N/A | 1.1 | N/A | 1.5 |
| Signaling by ERBB2 | N/A | 2.5 | N/A | 0.8 |
| Signaling by FGFR1 | N/A | 1.3 | N/A | 1.6 |
| Signaling by FGFR2 | N/A | -1.5 | N/A | 1.8 |
| Signaling by FGFR3 | N/A | 1.6 | N/A | 1.4 |
| Signaling by FGFR4 | N/A | 1.3 | N/A | 1.4 |
| Signaling by Insulin receptor | N/A | -1.7 | N/A | 1.0 |
| Signaling by NOTCH1 | N/A | 2.1 | N/A | 1.3 |
| Signaling by NOTCH2 | N/A | 1.6 | N/A | 1.7 |
| Signaling by NOTCH3 | N/A | 3.1 | N/A | 1.1 |
| Signaling by NTRK1 (TRKA) | N/A | -1.0 | N/A | 2.2 |
| Signaling by PDGF | N/A | 2.3 | N/A | -0.2 |
| Signaling by PTK6 | N/A | 0.3 | N/A | 3.5 |
| Signaling by Rho Family GTPases | N/A | 2.7 | 1.4 | 2.5 |
| Signaling by ROBO receptors | N/A | -2.3 | N/A | 2.0 |
| Signaling by SCF-KIT | N/A | 0.7 | N/A | 0.4 |
| Signaling by TGF-beta Receptor Complex | N/A | 2.4 | N/A | 2.2 |
| Signaling by TGFBR3 | N/A | 0.0 | N/A | 0.9 |
| Signaling by VEGF | N/A | 1.0 | 1.3 | 0.9 |
| Sleep NREM Signaling Pathway | N/A | 0.0 | N/A | -2.7 |
| Sleep REM Signaling Pathway | N/A | -2.1 | N/A | -1.2 |
| SNARE Signaling Pathway | N/A | 0.7 | 0.4 | 0.8 |
| Somitogenesis | N/A | -1.0 | N/A | 1.1 |
| Sphingosine-1-phosphate Signaling | N/A | 1.6 | N/A | 0.9 |
| Spliceosomal Cycle | N/A | -3.1 | N/A | 0.7 |
| SRP-dependent cotranslational protein targeting to membrane | N/A | -0.7 | N/A | 8.7 |
| STAT3 Pathway | N/A | 2.4 | N/A | 0.1 |
| SUMOylation of chromatin organization proteins | N/A | -2.1 | N/A | 1.2 |
| SUMOylation of DNA damage response and repair proteins | N/A | -3.3 | N/A | -0.6 |
| SUMOylation of DNA replication proteins | -2.4 | -2.4 | N/A | -0.4 |
| SUMOylation of RNA binding proteins | N/A | -2.4 | N/A | 0.8 |
| SUMOylation of transcription cofactors | N/A | -1.0 | N/A | 2.2 |
| Superpathway of Cholesterol Biosynthesis | -3.0 | -3.9 | 2.4 | 2.3 |
| Superpathway of Geranylgeranyldiphosphate Biosynthesis I (via Mevalonate) | -2.2 | -2.4 | N/A | 0.0 |
| Superpathway of Inositol Phosphate Compounds | N/A | 0.2 | N/A | 1.3 |
| Synaptic Long Term Potentiation | N/A | 0.5 | N/A | 2.3 |
| Synaptogenesis Signaling Pathway | N/A | 2.8 | 0.7 | 1.2 |
| Syndecan interactions | N/A | 1.7 | N/A | 0.0 |
| Synthesis of DNA | -3.5 | -6.6 | N/A | 1.4 |
| TAK1-dependent IKK and NF-kappa-B activation | N/A | 0.0 | N/A | 1.3 |
| TBC/RABGAPs | N/A | 1.6 | N/A | 0.9 |
| TCF dependent signaling in response to WNT | 1.0 | -1.4 | N/A | 1.7 |
| TCR signaling | N/A | -1.9 | N/A | 1.9 |
| Telomerase Signaling | N/A | 1.2 | N/A | -0.2 |
| Telomere Maintenance | -2.3 | -4.1 | N/A | 1.9 |
| TGF-β Signaling | N/A | 0.5 | N/A | 1.1 |
| Thrombin Signaling | N/A | 1.6 | N/A | 0.0 |
| Thrombopoietin Signaling | N/A | 1.0 | N/A | 0.6 |
| Thyroid Cancer Signaling | N/A | 1.4 | N/A | 1.6 |
| TNFR1 Signaling | N/A | -0.4 | N/A | 0.2 |
| TNFR2 non-canonical NF-kB pathway | N/A | -1.6 | N/A | 2.4 |
| TNFR2 Signaling | N/A | 0.0 | N/A | 2.1 |
| TP53 Regulates Metabolic Genes | N/A | 0.2 | N/A | 2.3 |
| TP53 Regulates Transcription of Cell Cycle Genes | -2.8 | -1.8 | N/A | 3.5 |
| TP53 Regulates Transcription of DNA Repair Genes | N/A | -2.7 | N/A | 0.5 |
| TR/RXR Activation | -1.1 | 1.3 | 1.3 | 0.2 |
| Transcriptional activity of SMAD2/SMAD3:SMAD4 heterotrimer | N/A | 0.4 | N/A | 2.1 |
| Transcriptional regulation by RUNX1 | N/A | -1.9 | N/A | 3.3 |
| Transcriptional regulation by RUNX3 | N/A | 2.4 | N/A | 1.8 |
| Transcriptional regulation by the AP-2 (TFAP2) family of transcription factors | N/A | -0.8 | N/A | 2.7 |
| Transcriptional regulation of granulopoiesis | N/A | -1.3 | N/A | 3.4 |
| Transcriptional Regulatory Network in Embryonic Stem Cells | N/A | 2.9 | N/A | 0.9 |
| trans-Golgi Network Vesicle Budding | N/A | -1.8 | N/A | 1.0 |
| Translocation of SLC2A4 (GLUT4) to the plasma membrane | N/A | -0.8 | N/A | 0.5 |
| Transport of inorganic cations/anions and amino acids/oligopeptides | N/A | 0.7 | 0.0 | 0.5 |
| tRNA Charging | N/A | -1.9 | N/A | -1.3 |
| UFMylation Signaling Pathway | N/A | -2.1 | N/A | 0.9 |
| Unfolded protein response | N/A | 1.0 | N/A | 3.7 |
| UVC-Induced MAPK Signaling | N/A | 0.0 | N/A | 2.2 |
| VDR/RXR Activation | N/A | -1.3 | N/A | 0.3 |
| VEGF Signaling | N/A | 1.2 | 2.6 | 0.7 |
| WNK Renal Signaling Pathway | N/A | 2.0 | N/A | -1.2 |
| WNT/SHH Axonal Guidance Signaling Pathway | N/A | 2.1 | N/A | -1.2 |
| XBP1(S) activates chaperone genes | N/A | 1.4 | N/A | 3.3 |
| α-Adrenergic Signaling | N/A | -0.3 | N/A | 2.0 |
|  |  |  |  |  |
|  |  |  |  |  |

Supplementary figure 9 Comparative analysis of canonical pathway (A) and upstream regulators (B) analysis by IPA with focus on lipid metabolism.

Supplementary figure 2, acid-mediated inhibition of lipid and cholesterol metabolism is repeatedly predicted in HK-2 cells (inhibition of “cholesterin synthesis”, of “superpathway of cholesterol biosynthesis”. Moreover, the upstream regulator analysis, predicted that this inhibition may be mediated by acid-induced suppression of RXRA, SCAP, SREBF1, and SREBF2.

Supplementary Table 9 List of enriched GO terms for upregulated DEGs in CCDSk after incubation with lactic acid.

| **source** | **term_name** | **adjusted_p_value** | **enrichment** |
| --- | --- | --- | --- |
| GO:MF | translation factor activity | 1.08E-11 | 3.24 |
| GO:BP | cytoplasmic translation | 5.22E-58 | 4.95 |
| GO:CC | cytosolic ribosome | 3.29E-53 | 5.63 |
| GO:CC | ribosomal subunit | 1.24E-46 | 4.30 |
| GO:CC | cytosolic large ribosomal subunit | 5.32E-33 | 6.34 |
| GO:CC | large ribosomal subunit | 4.23E-26 | 4.18 |
| GO:CC | cytosolic small ribosomal subunit | 3.53E-24 | 6.27 |
| GO:CC | small ribosomal subunit | 2.75E-19 | 4.40 |

Supplementary Table 10 Predicted acidosis-induced canonical pathways in CCDSk

| **Canonical Pathways** | **LA** |
| --- | --- |
| ABRA Signaling Pathway | 3.3 |
| Actin Cytoskeleton Signaling | 3.1 |
| Actin Nucleation by ARP-WASP Complex | 4.0 |
| Major pathway of rRNA processing in the nucleolus and cytosol | 7.0 |
| Cachexia Signaling Pathway | 3.5 |
| CDK5 Signaling | 3.5 |
| Mitotic G2-G2/M phases | 3.1 |
| Cellular response to heat stress | 3.4 |
| Cellular response to hypoxia | 3.1 |
| Ceramide Signaling | 5.0 |
| CGAS-STING Signaling Pathway | 4.3 |
| Cholecystokinin/Gastrin-mediated Signaling | 3.0 |
| Cristae formation | 3.3 |
| DNA Methylation and Transcriptional Repression Signaling | 3.3 |
| Ephrin Receptor Signaling | 3.7 |
| ERCC6 (CSB) and EHMT2 (G9a) positively regulate rRNA expression | 3.2 |
| ESR-mediated signaling | 3.3 |
| Eukaryotic Translation Elongation | 9.0 |
| Eukaryotic Translation Initiation | 9.4 |
| Eukaryotic Translation Termination | 8.5 |
| Formation of ATP by chemiosmotic coupling | 3.5 |
| Gap junction trafficking and regulation | 3.0 |
| Generic Transcription Pathway | -9.2 |
| Growth Hormone Signaling | -3.0 |
| Ribosomal Quality Control Signaling Pathway | 7.0 |
| HIF1α Signaling | 3.0 |
| HMGB1 Signaling | 3.2 |
| HSP90 chaperone cycle for steroid hormone receptors in the presence of ligand | 3.3 |
| Hypoxia Signaling in the Cardiovascular System | 3.1 |
| Interferon gamma signaling | 3.6 |
| Interleukin-10 signaling | 3.8 |
| Iron uptake and transport | 3.9 |
| L1CAM interactions | 3.3 |
| NAFLD Signaling Pathway | 3.4 |
| Interleukin-4 and Interleukin-13 signaling | 4.6 |
| Neutrophil degranulation | 6.4 |
| NGF-stimulated transcription | 4.4 |
| Nonsense-Mediated Decay (NMD) | 8.5 |
| NRF2-mediated Oxidative Stress Response | 3.6 |
| Oxidative Phosphorylation | 4.0 |
| Oxidative Stress Induced Senescence | 3.3 |
| Plasma lipoprotein assembly, remodeling, and clearance | 3.2 |
| Pre-NOTCH Expression and Processing | 3.3 |
| Protein folding | 4.0 |
| Regulation of Actin-based Motility by Rho | 4.0 |
| Degradation of the extracellular matrix | 3.1 |
| Response of EIF2AK4 (GCN2) to amino acid deficiency | 8.9 |
| Response to elevated platelet cytosolic Ca2+ | 3.2 |
| Role of Osteoblasts in Rheumatoid Arthritis Signaling Pathway | 4.5 |
| Post-translational protein phosphorylation | 3.1 |
| Selenoamino acid metabolism | 8.0 |
| Senescence-Associated Secretory Phenotype (SASP) | 3.8 |
| Regulation of Insulin-like Growth Factor (IGF) transport and uptake by IGFBPs | 3.5 |
| Signaling by PTK6 | 3.5 |
| SRP-dependent cotranslational protein targeting to membrane | 8.7 |
| TP53 Regulates Transcription of Cell Cycle Genes | 3.5 |
| Transcriptional regulation by RUNX1 | 3.3 |
| Transcriptional regulation of granulopoiesis | 3.4 |
| Unfolded protein response | 3.7 |
| XBP1(S) activates chaperone genes | 3.3 |
| Z-decay: degradation of maternal mRNAs by zygotically expressed factors | 3.2 |

Supplementary Table 11 Filtered list of predicted upstream regulators for HK-2, after incubation with lactic acid for 48 h.

| 1. Extracellular stimuli | | | | |
| --- | --- | --- | --- | --- |
| Upstream Regulator | Expr Log Ratio | Molecule Type | Activation z-score | p-value of overlap |
| CXCL12 |  | cytokine | 3.1 | 9.5E-05 |
| EDN1 |  | cytokine | 3.7 | 1.6E-07 |
| IFNG |  | cytokine | 5.1 | 3.4E-14 |
| IL13 |  | cytokine | 3.6 | 1.4E-05 |
| IL1A |  | cytokine | 6.0 | 5.8E-04 |
| IL1B |  | cytokine | 5.1 | 1.4E-20 |
| IL27 |  | cytokine | 4.8 | 6.9E-03 |
| IL6 | 2.39 | cytokine | 4.7 | 4.1E-07 |
| OSM |  | cytokine | 5.9 | 2.8E-11 |
| TNF |  | cytokine | 4.1 | 2.1E-24 |
| TNFSF11 |  | cytokine | 3.7 | 1.1E-06 |
| AGT | 1.47 | growth factor | 5.7 | 1.7E-18 |
| BMP6 |  | growth factor | 3.0 | 3.9E-04 |
| EGF | 1.41 | growth factor | 4.2 | 8.3E-21 |
| FGF2 |  | growth factor | 3.0 | 2.0E-11 |
| GDF2 |  | growth factor | 3.9 | 3.1E-05 |
| HBEGF |  | growth factor | 3.1 | 7.5E-05 |
| TGFA | 0.97 | growth factor | 3.2 | 4.9E-05 |
| TGFB1 | 1.32 | growth factor | 6.6 | 5.8E-42 |
| TGFB3 | -0.68 | growth factor | 3.1 | 3.3E-07 |
| 2. Membrane bound receptors | | | | |
| SUCNR1 |  | g-protein coupled receptor | 3.2 | 3.7E-04 |
| CCR2 |  | g-protein coupled receptor | 3.3 | 1.5E-03 |
| GPR174 |  | g-protein coupled receptor | 3.3 | 2.6E-02 |
| GRIN2A |  | ion channel | 3.0 | 3.3E-05 |
| STING1 |  | ion channel | 3.5 | 1.0E-01 |
| TNFRSF9 | -1.58 | transmembrane receptor | -5.0 | 3.6E-10 |
| CD3E |  | transmembrane receptor | -3.4 | 1.0E-08 |
| AMFR |  | transmembrane receptor | -3.7 | 2.7E-07 |
| IFNAR1 |  | transmembrane receptor | 3.2 | 2.2E-03 |
| TLR3 |  | transmembrane receptor | 3.2 | 1.2E-02 |
| SORL1 |  | transporter | 4.6 | 1.4E-09 |
| STEAP3 |  | transporter | -3.6 | 3.2E-05 |
| 3. Enzymes | | | | |
| MAGI1 |  | enzyme | 5.3 | 1.1E-30 |
| KDM1A |  | enzyme | -4.3 | 8.6E-11 |
| RRAS2 |  | enzyme | 4.2 | 1.0E-10 |
| METTL3 |  | enzyme | 3.4 | 1.9E-07 |
| KDM5B |  | enzyme | 4.1 | 2.4E-07 |
| ST6GAL1 | -0.5 | enzyme | 3.2 | 2.2E-07 |
| FKBP10 | 0.9 | enzyme | -3.8 | 5.7E-06 |
| RHOJ |  | enzyme | 3.2 | 1.7E-04 |
| LAMA4 |  | enzyme | -3.2 | 8.1E-04 |
| TGM2 | 2.32 | enzyme | 3.5 | 1.5E-02 |
| KDM5A |  | enzyme | 4.3 | 1.7E-02 |
| NLRP3 |  | enzyme | 3.4 | 1.8E-02 |
| TXN |  | enzyme | 3.2 | 3.7E-02 |
| RIGI |  | enzyme | 3.1 | 3.8E-02 |
| PRKCSH |  | enzyme | 3.2 | 9.8E-02 |
| MAP2K1 |  | kinase | 3.4 | 8.0E-09 |
| MAP2K5 |  | kinase | -3.1 | 8.3E-09 |
| MAPK14 |  | kinase | 3.0 | 1.8E-07 |
| FGFR1 | 0.6 | kinase | 3.8 | 1.6E-06 |
| ROCK1 |  | kinase | 3.4 | 1.6E-05 |
| CDK6 |  | kinase | 3.0 | 8.5E-05 |
| IKBKG |  | kinase | 3.0 | 3.4E-04 |
| JAK1 |  | kinase | 3.8 | 1.8E-03 |
| PRKAA2 | 0.6 | kinase | -4.1 | 2.0E-03 |
| MAPK3 |  | kinase | 3.6 | 3.8E-02 |
| MAPKAPK2 |  | kinase | 3.4 | 4.3E-02 |
| MAP3K1 |  | kinase | 3.4 | 4.9E-02 |
| LACTB |  | peptidase | -3.3 | 2.9E-13 |
| F2 | 2.5 | peptidase | 3.3 | 4.8E-07 |
| 4. Regulators of Translation | | | | |
| IGF2BP1 |  | translation regulator | 5.2 | 7.3E-22 |
| 5. Regulators of Transcription | | | | |
| ARNT |  | transcription regulator | 3.2 | 1.6E-07 |
| BHLHE40 | 0.65 | transcription regulator | 4.3 | 5.0E-07 |
| CBX5 | -0.71 | transcription regulator | -3.0 | 8.6E-04 |
| CEBPD | 0.61 | transcription regulator | 3.2 | 4.4E-05 |
| CEBPE |  | transcription regulator | 3.7 | 7.1E-03 |
| E2F1 | -0.96 | transcription regulator | -3.3 | 1.9E-15 |
| E2F3 |  | transcription regulator | -3.3 | 9.4E-06 |
| EPAS1 | 0.93 | transcription regulator | 4.3 | 2.0E-09 |
| FOXC1 |  | transcription regulator | 3.1 | 9.2E-06 |
| GATA1 |  | transcription regulator | 4.0 | 1.2E-05 |
| GLI1 | 0.99 | transcription regulator | -5.3 | 9.9E-28 |
| GMNN | -1.06 | transcription regulator | -3.4 | 4.4E-02 |
| HIF1A |  | transcription regulator | 6.3 | 6.5E-16 |
| IRF7 |  | transcription regulator | 3.3 | 4.6E-01 |
| KLF4 |  | transcription regulator | 3.1 | 8.4E-05 |
| KLF6 |  | transcription regulator | 3.5 | 9.3E-14 |
| MGA | -0.73 | transcription regulator | -3.1 | 1.5E-06 |
| MYC |  | transcription regulator | -3.8 | 9.9E-20 |
| MYCN |  | transcription regulator | -3.1 | 2.9E-07 |
| NFATC2 |  | transcription regulator | 3.6 | 4.5E-02 |
| NPM1 |  | transcription regulator | 5.6 | 8.8E-12 |
| NUPR1 |  | transcription regulator | 3.5 | 3.3E-26 |
| PAX6 | 0.97 | transcription regulator | 3.8 | 2.9E-02 |
| PHF12 |  | transcription regulator | 3.3 | 2.6E-03 |
| PPARGC1A |  | transcription regulator | -3.0 | 2.8E-12 |
| PTTG1 |  | transcription regulator | 3.9 | 1.1E-02 |
| RB1 | -0.64 | transcription regulator | 3.1 | 7.2E-16 |
| RBL1 | -1.15 | transcription regulator | 3.3 | 1.5E-05 |
| RELA |  | transcription regulator | 3.7 | 9.6E-04 |
| RXRA |  | ligand-dependent nuclear receptor | -4.3 | 4.0E-04 |
| RXRB |  | ligand-dependent nuclear receptor | -3.9 | 1.2E-04 |
| SIRT2 |  | transcription regulator | -3.4 | 9.5E-04 |
| SIX2 |  | transcription regulator | 4.0 | 3.8E-06 |
| SMAD3 | 1.21 | transcription regulator | 4.3 | 7.3E-09 |
| SMARCA4 |  | transcription regulator | 3.6 | 4.7E-21 |
| SOX1 |  | transcription regulator | -3.7 | 1.1E-01 |
| SOX3 |  | transcription regulator | -3.7 | 1.7E-01 |
| SREBF1 | -1.08 | transcription regulator | -3.1 | 5.0E-08 |
| SREBF2 |  | transcription regulator | -3.9 | 2.2E-11 |
| STAT1 | 1.05 | transcription regulator | 3.4 | 6.2E-05 |
| STAT3 |  | transcription regulator | 4.6 | 2.2E-09 |
| STAT4 | 1.66 | transcription regulator | 4.1 | 2.9E-08 |
| TARDBP | -0.57 | transcription regulator | -3.2 | 3.2E-09 |
| TBX2 |  | transcription regulator | -4.1 | 1.2E-10 |
| TFEB | 0.83 | transcription regulator | -7.3 | 6.0E-32 |
| TP53 | -1.19 | transcription regulator | 5.1 | 5.7E-35 |
| 6. Complex/groups | | | | |
| ALPHA CATENIN (family) |  | group | -4.8 | 5.2E-04 |
| CG (complex) |  | complex | 4.0 | 8.7E-23 |
| E2F (family) |  | group | -3.8 | 4.2E-14 |
| EGLN |  | group | -4.3 | 9.4E-07 |
| ERK (family) |  | group | 4.8 | 1.0E-11 |
| ERK1/2 (family) |  | group | 3.6 | 6.3E-05 |
| IFN ALPHA/BETA (family) |  | group | 3.1 | 3.1E-01 |
| IFNAR (family) |  | group | 3.1 | 9.8E-02 |
| IL1 (family) |  | group | 3.2 | 5.7E-05 |
| IL1R (family) |  | group | 3.9 | 7.1E-03 |
| INTERFERON ALPHA (family) |  | group | 4.4 | 3.9E-06 |
| MAPK (family) |  | group | 3.2 | 3.7E-02 |
| NFKB (complex) |  | complex | 5.0 | 6.8E-06 |
| P38 MAPK (family) |  | group | 3.8 | 1.6E-04 |
| PKC (family) |  | group | 3.2 | 2.6E-03 |
| RB (family) |  | group | 3.9 | 1.3E-06 |
| TGF BETA (family) |  | group | 4.6 | 2.5E-09 |

Supplementary Table 12 Filtered list of predicted upstream regulators for CCDSk, after incubation with lactic acid for 48 h.

| Upstream Regulator | Expr Log Ratio | Molecule Type | Activation z-score | p-value of overlap |
| --- | --- | --- | --- | --- |
| 1 Extracellular stimuli | | | | |
| TNF |  | cytokine | 10.2 | 1.0E-50 |
| IL6 | 7.7 | cytokine | 8.0 | 4.7E-12 |
| IFNG |  | cytokine | 7.3 | 1.3E-26 |
| OSM |  | cytokine | 4.4 | 1.7E-21 |
| IL2 |  | cytokine | 7.6 | 2.6E-21 |
| CMTM3 |  | cytokine | 4.7 | 9.5E-18 |
| IL4 |  | cytokine | 7.3 | 1.2E-16 |
| IL1B | 4.6 | cytokine | 7.9 | 1.1E-37 |
| IL3 |  | cytokine | 5.9 | 6.1E-16 |
| IL1A | 5.3 | cytokine | 7.1 | 2.1E-09 |
| TNFSF11 |  | cytokine | 6.9 | 5.4E-15 |
| CD40LG |  | cytokine | 4.8 | 3.1E-13 |
| IL15 |  | cytokine | 6.4 | 1.0E-12 |
| CSF2 | 12.0 | cytokine | 7.1 | 1.6E-15 |
| EDN1 | 2.8 | cytokine | 5.4 | 2.8E-16 |
| IL5 |  | cytokine | 7.1 | 3.2E-10 |
| IL33 |  | cytokine | 4.4 | 1.6E-09 |
| CSF1 | 2.7 | cytokine | 5.3 | 2.1E-12 |
| LIF | 6.6 | cytokine | 4.3 | 4.7E-06 |
| C5 |  | cytokine | 5.1 | 1.8E-08 |
| SPP1 |  | cytokine | 3.1 | 1.9E-08 |
| PF4 |  | cytokine | 3.3 | 2.0E-08 |
| IL17A |  | cytokine | 6.4 | 8.6E-07 |
| THPO |  | cytokine | 3.3 | 2.0E-06 |
| CNTF | 4.8 | cytokine | 3.8 | 1.1E-03 |
| CXCL12 | -1.2 | cytokine | 3.6 | 5.3E-09 |
| TNFSF13B |  | cytokine | 3.8 | 1.1E-04 |
| TNFSF14 |  | cytokine | 3.3 | 1.4E-04 |
| CCL5 |  | cytokine | 3.7 | 1.0E-03 |
| IL7 | 2.6 | cytokine | 3.1 | 1.1E-04 |
| IL36A |  | cytokine | 3.1 | 1.8E-03 |
| IL37 |  | cytokine | -3.1 | 1.8E-03 |
| LTA |  | cytokine | 3.0 | 2.5E-03 |
| IL22 |  | cytokine | 4.0 | 3.5E-03 |
| GPR15LG |  | cytokine | 3.2 | 3.8E-03 |
| IL18 |  | cytokine | 3.8 | 4.3E-03 |
| CCL2 | 2.9 | cytokine | 3.1 | 1.1E-02 |
| IL21 |  | cytokine | 3.5 | 2.9E-01 |
| TGFB1 | 2.9 | growth factor | 9.2 | 5.7E-47 |
| EGF |  | growth factor | 9.1 | 1.0E-33 |
| HGF |  | growth factor | 7.2 | 6.5E-31 |
| FGF2 |  | growth factor | 6.1 | 1.1E-23 |
| AGT |  | growth factor | 7.1 | 3.6E-23 |
| IGF1 |  | growth factor | 5.6 | 4.6E-21 |
| VEGFA | 3.2 | growth factor | 5.6 | 5.7E-14 |
| ANGPT2 |  | growth factor | 4.4 | 3.1E-13 |
| TGFB3 | -1.7 | growth factor | 5.1 | 1.8E-12 |
| NRG1 | 1.0 | growth factor | 4.3 | 1.8E-12 |
| NGF | 2.2 | growth factor | 5.5 | 6.0E-11 |
| BMP4 | -1.5 | growth factor | 4.6 | 1.5E-10 |
| GDF2 |  | growth factor | 4.4 | 2.1E-09 |
| BMP2 | 3.6 | growth factor | 4.4 | 6.9E-09 |
| KITLG | -1.3 | growth factor | 4.6 | 2.3E-07 |
| TGFA | 3.1 | growth factor | 4.4 | 8.4E-06 |
| HBEGF | 6.1 | growth factor | 4.8 | 6.8E-05 |
| PGF |  | growth factor | 3.2 | 1.4E-04 |
| 2 Membranebound receptors | | | | |
| SSTR2 |  | g-protein coupled receptor | -3.3 | 2.2E-12 |
| F2RL1 | 1.6 | g-protein coupled receptor | 3.6 | 1.9E-03 |
| F2R |  | g-protein coupled receptor | 3.7 | 4.2E-10 |
| GPER1 | -0.9 | g-protein coupled receptor | 3.5 | 3.4E-15 |
| GPR174 |  | g-protein coupled receptor | 3.3 | 2.6E-03 |
| PTGER3 |  | g-protein coupled receptor | 3.0 | 7.9E-03 |
| Mrgpra2a (includes others) |  | g-protein coupled receptor | 3.0 | 2.5E-01 |
| STING1 |  | ion channel | 4.5 | 4.3E-05 |
| Orai1 |  | ion channel | 3.2 | 7.5E-05 |
| TREM1 |  | transmembrane receptor | 5.7 | 6.0E-21 |
| TLR3 | -1.2 | transmembrane receptor | 5.1 | 3.3E-04 |
| TLR4 | -1.1 | transmembrane receptor | 5.1 | 4.6E-11 |
| CD28 |  | transmembrane receptor | 5.9 | 8.2E-12 |
| CD40 |  | transmembrane receptor | 6.0 | 3.0E-10 |
| CD36 |  | transmembrane receptor | 4.1 | 2.2E-07 |
| IL6R | 3.9 | transmembrane receptor | 3.2 | 3.5E-04 |
| TLR9 |  | transmembrane receptor | 4.6 | 4.3E-05 |
| CD200R1 |  | transmembrane receptor | -3.1 | 7.3E-05 |
| IGF1R | -1.0 | transmembrane receptor | 3.0 | 4.0E-06 |
| TLR7 |  | transmembrane receptor | 4.1 | 2.6E-03 |
| TLR2 |  | transmembrane receptor | 3.4 | 4.9E-03 |
| ICAM1 | 4.8 | transmembrane receptor | 3.2 | 1.9E-02 |
| CD5 |  | transmembrane receptor | 3.2 | 2.2E-02 |
| CD14 |  | transmembrane receptor | 3.3 | 3.0E-02 |
| TLR5 |  | transmembrane receptor | 3.1 | 3.8E-02 |
| SYVN1 |  | transporter | 3.5 | 3.6E-13 |
| GSDMD |  | transporter | 4.3 | 2.7E-11 |
| SLC15A4 | 1.6 | transporter | 4.0 | 3.0E-06 |
| STX18 |  | transporter | 3.4 | 1.5E-08 |
| ALB |  | transporter | 4.0 | 3.4E-06 |
| TFRC | 1.2 | transporter | -3.2 | 1.3E-04 |
| FDX1 | 0.6 | transporter | -3.2 | 1.2E-10 |
| APOE | 3.5 | transporter | -3.6 | 7.6E-13 |
| 3 Enzymes | | | | |
| ERBB2 | -1.9 | kinase | 6.0 | 2.6E-21 |
| F2 |  | peptidase | 7.6 | 1.7E-26 |
| EGFR |  | kinase | 6.4 | 9.1E-24 |
| PRKCD | 1.0 | kinase | 5.8 | 7.6E-15 |
| MAP3K8 | 2.3 | kinase | 5.6 | 1.8E-09 |
| IKBKB | -1.0 | kinase | 5.4 | 3.1E-16 |
| RAF1 |  | kinase | 5.9 | 3.0E-20 |
| MAP2K1 |  | kinase | 4.1 | 1.5E-18 |
| HRAS | 1.1 | enzyme | 5.0 | 1.6E-30 |
| JAK1 | 0.8 | kinase | 4.5 | 7.8E-08 |
| GNAQ |  | enzyme | 3.7 | 3.6E-15 |
| IKBKG | -1.0 | kinase | 4.5 | 9.6E-09 |
| DDX3X | 1.3 | enzyme | 4.2 | 1.7E-07 |
| RET |  | kinase | 3.2 | 9.2E-13 |
| EIF2AK2 | -0.8 | kinase | 3.9 | 9.3E-05 |
| AKT1 |  | kinase | 4.0 | 7.8E-12 |
| MAPK14 |  | kinase | 4.7 | 4.8E-11 |
| RNF138 | 1.6 | enzyme | 3.9 | 2.0E-07 |
| CHUK |  | kinase | 5.1 | 2.6E-10 |
| CHD1 | 2.2 | enzyme | 3.8 | 3.1E-10 |
| GNAS | 1.1 | enzyme | 3.8 | 8.0E-09 |
| PDHA1 |  | enzyme | 3.6 | 2.0E-09 |
| MAP2K3 | 3.8 | kinase | 3.8 | 1.7E-03 |
| EIF2AK3 | 2.1 | kinase | 3.7 | 1.6E-10 |
| RAC1 | 0.9 | enzyme | 3.6 | 9.0E-05 |
| PRKCE |  | kinase | 4.6 | 1.1E-08 |
| TRAF6 | 1.5 | enzyme | 3.6 | 6.9E-03 |
| ARL3 |  | enzyme | -3.0 | 4.7E-08 |
| MAPK3 |  | kinase | 4.7 | 5.2E-08 |
| SRC |  | kinase | 4.6 | 5.6E-08 |
| IPMK | 2.9 | kinase | 3.6 | 1.9E-04 |
| CDK6 |  | kinase | 3.3 | 8.8E-08 |
| RRAS2 | 0.8 | enzyme | 3.6 | 1.7E-04 |
| MET |  | kinase | 3.7 | 1.7E-07 |
| INSR | -0.9 | kinase | 3.5 | 1.3E-07 |
| BRD4 | 1.9 | kinase | 3.4 | 2.3E-13 |
| CD38 |  | enzyme | 3.9 | 2.4E-07 |
| MAPK7 |  | kinase | 3.5 | 3.2E-07 |
| DCLK1 | -1.9 | kinase | 3.3 | 7.1E-04 |
| GNA13 | 3.1 | enzyme | 3.3 | 3.7E-07 |
| ADAM17 |  | peptidase | 3.8 | 1.6E-06 |
| TGM2 | 1.7 | enzyme | 3.2 | 1.3E-08 |
| BTK |  | kinase | 3.9 | 4.4E-06 |
| CDK9 | 0.9 | kinase | 3.2 | 3.1E-09 |
| KAT2A |  | enzyme | 3.7 | 1.4E-05 |
| MAOA |  | enzyme | 3.5 | 2.0E-05 |
| LCK |  | kinase | 3.3 | 6.5E-05 |
| SKIC2 |  | enzyme | -3.3 | 7.9E-05 |
| RAD51 |  | enzyme | -3.7 | 8.9E-05 |
| MAP3K14 | 0.6 | kinase | 3.2 | 5.7E-04 |
| DDR2 | -1.1 | kinase | 3.1 | 1.7E-04 |
| LIPA |  | enzyme | -3.6 | 1.2E-04 |
| DCAF1 |  | kinase | -3.5 | 1.4E-04 |
| FGFR1 | 0.6 | kinase | 3.1 | 1.4E-15 |
| PIK3R1 | -2.1 | kinase | 3.1 | 1.3E-12 |
| NCF1 |  | enzyme | 3.4 | 1.8E-04 |
| GPAT4 | -0.7 | enzyme | 3.0 | 4.0E-07 |
| KAT2B |  | enzyme | 3.0 | 2.5E-04 |
| USP14 |  | peptidase | 3.0 | 3.7E-04 |
| MAP3K1 |  | kinase | 3.5 | 4.1E-04 |
| Usp17la (includes others) |  | peptidase | 3.1 | 4.9E-04 |
| Alox15 |  | enzyme | 3.1 | 5.0E-04 |
| RIGI |  | enzyme | 3.6 | 5.0E-04 |
| BACE1 |  | peptidase | -4.0 | 5.2E-04 |
| UBA1 | -0.6 | enzyme | -3.2 | 1.7E-06 |
| HIBCH | -1.4 | enzyme | -3.3 | 1.3E-05 |
| USP18 |  | peptidase | -3.0 | 8.4E-04 |
| TGFBR2 | -0.9 | kinase | -3.4 | 2.2E-20 |
| MKNK1 |  | kinase | 3.7 | 3.4E-03 |
| CSF1R |  | kinase | 3.1 | 4.7E-03 |
| MAP2K4 |  | kinase | 3.2 | 5.1E-03 |
| CDK19 | -1.1 | kinase | -3.8 | 4.3E-22 |
| ILK |  | kinase | 3.1 | 2.6E-02 |
| TRAF3IP2 |  | enzyme | 3.5 | 3.3E-02 |
| IFIH1 | -1.6 | enzyme | 3.1 | 5.4E-02 |
| CAB39L | 0.8 | kinase | 3.1 | 5.6E-02 |
| 4 Regulators of translation | | | | |
| LARP1 | 1.2 | translation regulator | -8.9 | 5.7E-59 |
| FMR1 |  | translation regulator | -5.5 | 2.4E-28 |
| EIF4E | 2.1 | translation regulator | 5.7 | 1.1E-08 |
| RPL22 | 1.2 | translation regulator | -3.1 | 3.9E-06 |
| RPSA | 1.8 | translation regulator | -3.3 | 1.5E-04 |
| 5 Regulators of Transcription | | | | |
| NUPR1 | 1.3 | transcription regulator | 10.9 | 7.8E-42 |
| MYC | 5.9 | transcription regulator | 7.8 | 8.3E-51 |
| MYCN |  | transcription regulator | 5.2 | 3.1E-45 |
| RELA | 0.9 | transcription regulator | 7.3 | 4.0E-17 |
| SPEN | 2.4 | transcription regulator | 7.2 | 1.4E-41 |
| CTNNB1 |  | transcription regulator | 5.0 | 2.0E-41 |
| MLXIPL |  | transcription regulator | 8.2 | 1.7E-35 |
| KLF6 | 2.1 | transcription regulator | 6.9 | 8.9E-28 |
| JUN | 4.6 | transcription regulator | 6.3 | 4.4E-16 |
| HIF1A | 2.6 | transcription regulator | 6.0 | 1.3E-19 |
| CREB1 |  | transcription regulator | 7.9 | 7.6E-23 |
| ESR2 |  | ligand-dependent nuclear receptor | 3.3 | 3.0E-21 |
| NPM1 | 1.2 | transcription regulator | 5.8 | 1.8E-20 |
| NFAT5 | 1.0 | transcription regulator | 5.6 | 5.1E-06 |
| PGR |  | ligand-dependent nuclear receptor | 5.1 | 2.7E-19 |
| CEBPB | 5.2 | transcription regulator | 5.4 | 1.8E-09 |
| STAT3 |  | transcription regulator | 6.3 | 1.2E-17 |
| SMAD3 | 0.6 | transcription regulator | 5.2 | 1.6E-13 |
| HMG20A | -0.9 | transcription regulator | 5.2 | 4.2E-16 |
| YAP1 | -0.7 | transcription regulator | 4.9 | 7.9E-50 |
| TFEB | 1.7 | transcription regulator | 4.8 | 1.1E-15 |
| JUNB | 7.6 | transcription regulator | 4.7 | 3.5E-06 |
| COPS5 |  | transcription regulator | -3.8 | 1.6E-14 |
| FOS | 7.5 | transcription regulator | 4.7 | 4.4E-24 |
| ARNT2 | -1.0 | transcription regulator | 4.7 | 1.1E-03 |
| ATF4 | 2.0 | transcription regulator | 4.7 | 3.4E-14 |
| SP1 | 0.9 | transcription regulator | 4.5 | 4.3E-19 |
| ECSIT | 1.1 | transcription regulator | 4.4 | 1.5E-11 |
| SS18 | -0.6 | transcription regulator | 4.1 | 1.0E-05 |
| SRF | 1.1 | transcription regulator | 4.1 | 1.8E-07 |
| POU5F1 |  | transcription regulator | 3.1 | 3.7E-11 |
| NFKB1 |  | transcription regulator | 5.4 | 4.7E-11 |
| AHR |  | ligand-dependent nuclear receptor | 4.0 | 2.7E-10 |
| CREM |  | transcription regulator | 3.7 | 2.7E-10 |
| BHLHE40 | 8.0 | transcription regulator | 4.1 | 2.3E-08 |
| STAT4 |  | transcription regulator | 6.6 | 1.0E-09 |
| ETS1 | 4.0 | transcription regulator | 4.1 | 1.3E-07 |
| GLI3 |  | transcription regulator | 3.4 | 3.4E-09 |
| FOXA1 |  | transcription regulator | -3.8 | 4.6E-09 |
| EGR1 | 3.0 | transcription regulator | 4.0 | 4.7E-16 |
| NFE2L2 | 1.3 | transcription regulator | 3.8 | 8.9E-14 |
| CREBBP |  | transcription regulator | 3.4 | 2.1E-08 |
| XBP1 | 1.5 | transcription regulator | 3.6 | 8.3E-09 |
| E2F3 | 1.5 | transcription regulator | 3.6 | 6.9E-04 |
| KDM3A |  | transcription regulator | 3.2 | 1.1E-07 |
| ARNT |  | transcription regulator | 3.1 | 1.3E-07 |
| PPRC1 | 1.9 | transcription regulator | 3.5 | 9.0E-04 |
| YBX1 |  | transcription regulator | 4.3 | 1.4E-07 |
| FOXO1 | 1.1 | transcription regulator | 3.4 | 5.2E-10 |
| MYCL |  | transcription regulator | 3.6 | 5.9E-07 |
| FOXL2 |  | transcription regulator | 3.7 | 8.0E-07 |
| ELK1 | 2.7 | transcription regulator | 3.3 | 3.1E-12 |
| GFI1 |  | transcription regulator | -3.6 | 1.3E-06 |
| NFKBIZ | 4.0 | transcription regulator | 3.2 | 3.8E-04 |
| ATF6 | -0.9 | transcription regulator | 3.2 | 1.1E-06 |
| E2F1 | 2.1 | transcription regulator | 3.1 | 5.8E-14 |
| ACTN4 |  | transcription regulator | 3.1 | 6.3E-06 |
| NFKBIA | 6.2 | transcription regulator | 3.1 | 5.3E-25 |
| POU2F2 | 1.2 | transcription regulator | 3.1 | 1.2E-05 |
| FLI1 |  | transcription regulator | 3.6 | 2.8E-05 |
| NCOA3 |  | transcription regulator | 4.0 | 6.8E-05 |
| SMAD2 |  | transcription regulator | 4.4 | 6.8E-05 |
| STAT5B |  | transcription regulator | 4.0 | 1.1E-04 |
| TFE3 | 1.9 | transcription regulator | 3.1 | 3.6E-04 |
| EPAS1 | 1.0 | transcription regulator | 3.1 | 2.0E-12 |
| ZBTB7A | 1.4 | transcription regulator | -3.3 | 2.7E-04 |
| MEF2C |  | transcription regulator | 3.2 | 6.4E-04 |
| CITED2 | 1.3 | transcription regulator | -3.6 | 5.9E-09 |
| HOXA10 | -1.3 | transcription regulator | -3.6 | 8.1E-04 |
| HIVEP1 | 1.1 | transcription regulator | -4.0 | 2.2E-06 |
| ZFP36 | 6.7 | transcription regulator | -4.7 | 9.3E-08 |
| CREBZF |  | transcription regulator | -3.4 | 3.4E-03 |
| SIM1 |  | transcription regulator | 4.4 | 1.2E-02 |
| PTTG1 | 2.0 | transcription regulator | 3.5 | 1.2E-02 |
| ASXL1 |  | transcription regulator | -3.2 | 2.1E-02 |
| HAND2 |  | transcription regulator | 3.1 | 2.6E-02 |
| MNT | 2.0 | transcription regulator | -3.5 | 2.6E-02 |
| IKZF2 |  | transcription regulator | -3.2 | 4.8E-02 |
| 6 Complex, groups | | | | |
| LH (complex) |  | complex | 8.5 | 3.0E-44 |
| PDGF-BB (complex) |  | complex | 8.7 | 4.1E-38 |
| CG (complex) |  | complex | 5.4 | 8.9E-32 |
| NFKB (complex) |  | complex | 9.2 | 4.6E-24 |
| TCR (complex) |  | complex | 6.3 | 2.0E-23 |
| MEK (family) |  | group | 3.5 | 3.3E-22 |
| CD3 (complex) |  | complex | 6.9 | 2.2E-19 |
| ERK (family) |  | group | 6.8 | 2.3E-18 |
| VEGF (family) |  | group | 7.4 | 1.8E-17 |
| IGE (complex) |  | complex | 6.3 | 2.7E-17 |
| PDGF (complex) |  | complex | 5.3 | 8.6E-17 |
| LDL (complex) |  | complex | 3.2 | 7.4E-16 |
| RAS (family) |  | group | 3.9 | 3.8E-15 |
| ERK1/2 (family) |  | group | 5.7 | 1.3E-14 |
| BCR (complex) |  | complex | 6.0 | 4.0E-14 |
| TGF BETA (family) |  | group | 4.1 | 5.8E-14 |
| P38 MAPK (family) |  | group | 7.1 | 1.0E-13 |
| INSULIN (family) |  | group | 6.0 | 1.8E-13 |
| PI3K (complex) |  | complex | 4.0 | 5.5E-13 |
| JNK (family) |  | group | 4.8 | 2.2E-11 |
| CDK (family) |  | group | 3.6 | 4.0E-11 |
| INTERFERON ALPHA (family) |  | group | 4.3 | 5.1E-11 |
| ALPHA CATENIN (family) |  | group | -3.2 | 1.1E-10 |
| PKC (family) |  | group | 4.2 | 1.8E-10 |
| CYTOKINE (family) |  | group | 5.0 | 5.1E-10 |
| IL1 (family) |  | group | 7.2 | 1.3E-09 |
| PKA (complex) |  | complex | 3.1 | 9.1E-09 |
| CREB (family) |  | group | 4.7 | 1.1E-08 |
| TNF (family) |  | group | 4.7 | 2.3E-08 |
| MAP2K1/2 (family) |  | group | 3.8 | 4.5E-08 |
| STAT1/3/5 DIMER (complex) |  | complex | 4.0 | 4.7E-08 |
| TSH (complex) |  | complex | 3.4 | 7.1E-08 |
| AP1 (complex) |  | complex | 5.6 | 1.1E-07 |
| EGLN (family) |  | group | -3.5 | 1.5E-06 |
| CSF (family) |  | group | 3.6 | 1.7E-06 |
| MAPK (family) |  | group | 4.0 | 2.4E-06 |
| G PROTEIN ALPHA I (family) |  | group | 4.9 | 2.7E-06 |
| STAT5A/B |  | group | 3.1 | 8.6E-06 |
| IL1R (family) |  | group | 4.7 | 9.2E-06 |
| TCF/LEF (family) |  | group | 3.2 | 2.2E-05 |
| COLLAGENASE (family) |  | group | 3.4 | 2.5E-05 |
| NFKB (family) |  | group | 4.3 | 4.4E-05 |
| COLLAGEN TYPE I (complex) |  | complex | 3.8 | 6.9E-05 |
| TLR7/8 (family) |  | group | 3.5 | 7.5E-05 |
| NFKB-RELA (complex) |  | complex | 3.7 | 2.6E-04 |
| HIF1 (complex) |  | complex | 3.4 | 2.6E-04 |
| NFKB1-RELA (complex) |  | complex | 4.0 | 4.7E-04 |
| STAT3/STAT3 (complex) |  | complex | 3.5 | 5.7E-04 |
| FCER1 (complex) |  | complex | 3.4 | 8.4E-04 |
| NFAT (family) |  | group | 3.6 | 8.9E-04 |
| STAT (family) |  | group | 3.7 | 9.5E-04 |
| E2F (family) |  | group | 3.2 | 1.1E-03 |
| SAA |  | group | 3.1 | 2.3E-03 |
| NGF (family) |  | group | 3.2 | 2.5E-03 |
| IL12 (family) |  | group | 3.6 | 1.2E-02 |
| TLR (family) |  | group | 3.3 | 2.0E-02 |
| IL12 (complex) |  | complex | 3.4 | 2.3E-02 |

Supplementary Table 13 Local network clusters of upstream regulators from HK-2 cells identified by STRING analysis

| **cluster** | **description** | **count in network** | **strength** | **signal** | **false discovery rate** |  |  |  |
| --- | --- | --- | --- | --- | --- | --- | --- | --- |
| CL:15940 | JAK-STAT signaling pathway, and Interleukin-1 family | 15 of 138 | 1.1 | 1.6 | 3.3E-08 |  |  | cell cycle/ proliferation |
| CL:6900 | G1 Phase, and Polo-like kinase mediated events | 9 of 44 | 1.4 | 1.5 | 1.1E-06 |  |  | Inflammatory response |
| CL:15942 | JAK-STAT signaling pathway | 12 of 103 | 1.1 | 1.4 | 7.5E-07 |  |  | MAPK |
| CL:6904 | Defective binding of RB1 mutants to E2F1,(E2F2, E2F3), and Cyclin-dependent protein serine/threonine kinase inhibitor activity | 6 of 21 | 1.5 | 1.1 | 7.0E-05 |  |  | Fatty acid metabolism |
| CL:17329 | Constitutive Signaling by Aberrant PI3K in Cancer, and VEGF ligand-receptor interactions | 9 of 71 | 1.1 | 1.1 | 3.2E-05 |  |  | Fibrotic response |
| CL:9989 | Regulation of cholesterol storage, and Cellular response to sterol depletion | 6 of 23 | 1.5 | 1.1 | 9.9E-05 |  |  |  |
| CL:15943 | JAK-STAT signaling pathway | 10 of 98 | 1.0 | 1.0 | 3.3E-05 |  |  |  |
| CL:17330 | Constitutive Signaling by Aberrant PI3K in Cancer, and VEGF ligand-receptor interactions | 8 of 61 | 1.2 | 1.0 | 9.9E-05 |  |  |  |
| CL:17423 | ERBB2-EGFR signaling pathway | 4 of 7 | 1.8 | 0.9 | 5.4E-04 |  |  |  |
| CL:20825 | Mixed, incl. Steroid-binding, and Steroid receptor coactivator | 5 of 19 | 1.5 | 0.9 | 5.4E-04 |  |  |  |
| CL:20824 | Mixed, incl. Loss of MECP2 binding ability to the NCoR/SMRT complex, and c4 zinc finger in nuclear hormone receptors | 6 of 36 | 1.3 | 0.8 | 5.4E-04 |  |  |  |
| CL:20713 | Mixed, incl. Transcription coregulator binding, and DNA-binding transcription repressor activity | 11 of 161 | 0.9 | 0.8 | 1.9E-04 |  |  |  |
| CL:20715 | Mixed, incl. Transcription coregulator binding, and Activation of the TFAP2 (AP-2) family of transcription factors | 9 of 104 | 1.0 | 0.8 | 3.0E-04 |  |  |  |
| CL:17328 | Mixed, incl. Constitutive Signaling by Aberrant PI3K in Cancer, and FCERI mediated Ca+2 mobilization | 10 of 133 | 0.9 | 0.8 | 2.6E-04 |  |  |  |
| CL:20827 | Mixed, incl. Steroid receptor coactivator, and Nuclear estrogen receptor activity | 4 of 11 | 1.6 | 0.8 | 1.6E-03 |  |  |  |
| CL:6936 | G2 Phase, and Familial retinoblastoma | 3 of 5 | 1.8 | 0.6 | 6.4E-03 |  |  |  |
| CL:20830 | Nuclear estrogen receptor activity, and PAS domain | 3 of 5 | 1.8 | 0.6 | 6.4E-03 |  |  |  |
| CL:16155 | Interleukin-1 conserved site, and Interleukin-1 receptor type 1 | 3 of 5 | 1.8 | 0.6 | 6.4E-03 |  |  |  |
| CL:10015 | NR1H2 & NR1H3 regulate gene expression to limit cholesterol uptake, and Cellular response to vitamin E | 3 of 6 | 1.7 | 0.6 | 8.0E-03 |  |  |  |
| CL:15945 | JAK-STAT signaling pathway | 6 of 63 | 1.0 | 0.6 | 6.4E-03 |  |  |  |
| CL:21275 | Signaling by BMP, and TGF-beta propeptide | 5 of 42 | 1.1 | 0.5 | 8.5E-03 |  |  |  |
| CL:9983 | Mixed, incl. Fatty acid metabolism, and Regulation of lipid storage | 7 of 98 | 0.9 | 0.5 | 7.1E-03 |  |  |  |
| CL:18397 | Mixed, incl. Basic-leucine zipper domain, and Host-pathogen interaction of human coronaviruses - MAPK signaling | 7 of 98 | 0.9 | 0.5 | 7.1E-03 |  |  |  |
| CL:6908 | Drug-mediated inhibition of CDK4/CDK6 activity, and Regulation of cardiac muscle tissue regeneration | 3 of 8 | 1.6 | 0.5 | 1.4E-02 |  |  |  |
| CL:19734 | Type III transforming growth factor beta receptor binding, and Transforming growth factor beta receptor complex assembly | 3 of 8 | 1.6 | 0.5 | 1.4E-02 |  |  |  |
| CL:16089 | IL-6-type cytokine receptor ligand interactions, and Positive regulation of NK T cell proliferation | 4 of 25 | 1.2 | 0.5 | 1.5E-02 |  |  |  |
| CL:17673 | Mixed, incl. Inhibition of exosome biogenesis and secretion by Manumycin A in CRPC cells, and Cardiofaciocutaneous syndrome | 4 of 26 | 1.2 | 0.5 | 1.7E-02 |  |  |  |
| CL:19697 | Elastic fibre formation, and Transforming growth factor beta receptor complex assembly | 4 of 27 | 1.2 | 0.5 | 1.9E-02 |  |  |  |
| CL:6967 | Transcription of E2F targets under negative control by p107 (RBL1) and p130 (RBL2) in complex with HDAC1 | 3 of 10 | 1.5 | 0.5 | 2.1E-02 |  |  |  |
| CL:9990 | Cellular response to sterol depletion, and Vitellogenin, N-terminal | 3 of 12 | 1.4 | 0.4 | 3.0E-02 |  |  |  |
| CL:3823 | Type 2 papillary renal cell carcinoma | 3 of 12 | 1.4 | 0.4 | 3.0E-02 |  |  |  |
| CL:19695 | Elastic fibre formation, and Matrix metalloproteinases | 5 of 61 | 1.0 | 0.4 | 3.2E-02 |  |  |  |
| CL:17676 | Negative feedback regulation of MAPK pathway, and RAS GTPase cycle mutants | 3 of 14 | 1.4 | 0.4 | 3.8E-02 |  |  |  |
| CL:19457 | Extracellular matrix organization | 8 of 180 | 0.7 | 0.4 | 3.2E-02 |  |  |  |
| CL:16584 | Toll-like Receptor Cascades, and Novel intracellular components of RIG-I-like receptor pathway | 6 of 105 | 0.8 | 0.3 | 4.4E-02 |  |  |  |
| CL:16582 | Toll-like Receptor Cascades, and Death-like domain superfamily | 7 of 150 | 0.7 | 0.3 | 4.6E-02 |  |  |  |
| CL:6897 | Mixed, incl. G1 Phase, and G0 and Early G1 | 12 of 67 | 1.3 | 1.8 | 3.3E-08 |  |  |  |

Supplementary Table 14 Local network clusters of upstream regulators from CCDSk cells identified by STRING analysis

| **cluster** | **description** | **count in network** | **strength** | **signal** | **false discovery rate** |  |  |  |
| --- | --- | --- | --- | --- | --- | --- | --- | --- |
| CL:18504 | Mixed, incl. Basic region leucin zipper, and Early growth response, N-terminal | 8 of 32 | 1.6 | 1.8 | 1.6E-07 |  |  | cell cycle/ proliferation |
| CL:16583 | Toll-like Receptor Cascades, and NF-kappa B signaling pathway | 12 of 115 | 1.3 | 1.7 | 5.1E-08 |  |  | Inflammatory response |
| CL:16585 | Mixed, incl. Novel intracellular components of RIG-I-like receptor pathway, and TNFR1-induced NFkappaB signaling pathway | 9 of 73 | 1.3 | 1.5 | 7.9E-07 |  |  | Early/ Integrated stress response |
| CL:16587 | Mixed, incl. TNFR1-induced NFkappaB signaling pathway, and NF-kappa-B/Dorsal | 7 of 43 | 1.4 | 1.4 | 5.6E-06 |  |  |  |
| CL:17327 | Mixed, incl. Constitutive Signaling by Aberrant PI3K in Cancer, and Tyrosine-protein kinase, catalytic domain | 11 of 154 | 1.1 | 1.3 | 2.0E-06 |  |  |  |
| CL:18397 | Mixed, incl. Basic-leucine zipper domain, and Host-pathogen interaction of human coronaviruses - MAPK signaling | 9 of 98 | 1.2 | 1.3 | 5.0E-06 |  |  |  |
| CL:17328 | Mixed, incl. Constitutive Signaling by Aberrant PI3K in Cancer, and FCERI mediated Ca+2 mobilization | 10 of 133 | 1.1 | 1.2 | 4.9E-06 |  |  |  |
| CL:1567 | Regulation of translation initiation in response to endoplasmic reticulum stress, and Regulation of ATF6-mediated unfolded protein response | 4 of 6 | 2.1 | 1.2 | 5.8E-05 |  |  |  |
| CL:18506 | Response of EIF2AK1 (HRI) to heme deficiency, and Transcription factor AP-1 complex | 5 of 18 | 1.7 | 1.2 | 5.6E-05 |  |  |  |
| CL:18395 | Mixed, incl. Basic-leucine zipper domain, and MAPK signaling pathway | 10 of 163 | 1.0 | 1.1 | 2.2E-05 |  |  |  |
| CL:17329 | Constitutive Signaling by Aberrant PI3K in Cancer, and VEGF ligand-receptor interactions | 7 of 71 | 1.2 | 1.0 | 8.0E-05 |  |  |  |
| CL:15940 | JAK-STAT signaling pathway, and Interleukin-1 family | 9 of 138 | 1.0 | 1.0 | 5.6E-05 |  |  |  |
| CL:16090 | IL-6-type cytokine receptor ligand interactions | 4 of 13 | 1.7 | 0.9 | 5.2E-04 |  |  |  |
| CL:15943 | JAK-STAT signaling pathway | 7 of 98 | 1.1 | 0.8 | 5.5E-04 |  |  |  |
| CL:19734 | Type III transforming growth factor beta receptor binding, and Transforming growth factor beta receptor complex assembly | 3 of 8 | 1.8 | 0.7 | 5.3E-03 |  |  |  |
| CL:17330 | Constitutive Signaling by Aberrant PI3K in Cancer, and VEGF ligand-receptor interactions | 5 of 61 | 1.1 | 0.6 | 6.7E-03 |  |  |  |
| CL:18544 | Mixed, incl. Early growth response, N-terminal, and NAB family | 3 of 12 | 1.6 | 0.5 | 1.3E-02 |  |  |  |
| CL:17422 | ERBB2 Activates PTK6 Signaling | 3 of 12 | 1.6 | 0.5 | 1.3E-02 |  |  |  |
| CL:17474 | Phosphatidylinositol 3-kinase complex, class I, and Insulin receptor complex | 3 of 18 | 1.5 | 0.4 | 3.2E-02 |  |  |  |
